# Supplementary material for: Stepwise polarisation of developing bilayered epidermis is mediated by aPKC and E-cadherin in zebrafish
Source: eLife. 2020 Jan 22;9:e49064. doi: 10.7554/eLife.49064 (PMC6975926; doi:10.7554/eLife.49064)
Supplement: Figure 2—source data 3. [file elife-49064-fig2-data3.docx]

Statistical comparisons between WT sibling and *has/apkc* mut periderm

**Mann-Whitney Rank Sum Test**

**For Height of cell as shown in Figure 2 A2**

**Normality Test (Shapiro-Wilk):**  Failed (P < 0.050)

**Group N Missing Median 25% 75%**

aPKC sib 124 0 2.520 1.960 3.080

aPKC mut 147 0 3.080 2.520 4.480

Mann-Whitney U Statistic= 5545.500

T = 13295.500 n(small)= 124 n(big)= 147 (P = <0.001)

The difference in the median values between the two groups is greater than would be expected by chance; there is a statistically significant difference (P = <0.001)

**Mann-Whitney Rank Sum Test**

**For Apical Perimeter as shown in Figure 2 A3**

**Normality Test (Shapiro-Wilk):**  Failed (P < 0.050)

**Group N Missing Median 25% 75%**

aPKC sib 124 0 80.198 74.165 87.685

aPKC mut 147 0 67.654 62.784 74.691

Mann-Whitney U Statistic= 3643.000

T = 22335.000 n(small)= 124 n(big)= 147 (P = <0.001)

The difference in the median values between the two groups is greater than would be expected by chance; there is a statistically significant difference (P = <0.001)

**For Percent cells showing abnormal distribution shown in Figure 2 A5 derived post linear regression fit as shown in Figure 2 - figure supplement 3 A**

| **Genotype** | **Distribution** | **Count** | **total** | **Percentage** |
| --- | --- | --- | --- | --- |
| **aPKC sib** | Abnormal | 10 | 124 | 8.064516 |
| **aPKC sib** | Normal | 114 | 124 | 91.93548 |
| **aPKC mut** | Abnormal | 35 | 147 | 23.80952 |
| **aPKC mut** | Normal | 112 | 147 | 76.19048 |
