# Supplementary material for: Stepwise polarisation of developing bilayered epidermis is mediated by aPKC and E-cadherin in zebrafish
Source: eLife. 2020 Jan 22;9:e49064. doi: 10.7554/eLife.49064 (PMC6975926; doi:10.7554/eLife.49064)
Supplement: Figure 2—source data 4. [file elife-49064-fig2-data4.docx]

Statistical comparisons between WT sibling and *has/apkc* mut basal epidermis

**Mann-Whitney Rank Sum Test**

**For Height of cell as shown in Figure 2 B2**

**Normality Test (Shapiro-Wilk):**  Failed (P < 0.050)

**Group N Missing Median 25% 75%**

aPKC sib 134 0 1.960 1.400 2.240

aPKC mut 129 0 1.680 1.400 2.240

Mann-Whitney U Statistic= 7370.000

T = 15755.000 n(small)= 129 n(big)= 134 (P = 0.037)

The difference in the median values between the two groups is greater than would be expected by chance; there is a statistically significant difference (P = 0.037)

**Mann-Whitney Rank Sum Test**

**For Apical Perimeter as shown in Figure 2 B3**

**Normality Test (Shapiro-Wilk):**  Failed (P < 0.050)

**Group N Missing Median 25% 75%**

aPKC sib 134 0 67.297 60.443 73.219

aPKC mut 129 0 65.395 57.345 73.628

Mann-Whitney U Statistic= 8041.500

T = 16426.500 n(small)= 129 n(big)= 134 (P = 0.330)

The difference in the median values between the two groups is not great enough to exclude the possibility that the difference is due to random sampling variability; there is not a statistically significant difference (P = 0.330)
