# Supplementary material for: Stepwise polarisation of developing bilayered epidermis is mediated by aPKC and E-cadherin in zebrafish
Source: eLife. 2020 Jan 22;9:e49064. doi: 10.7554/eLife.49064 (PMC6975926; doi:10.7554/eLife.49064)
Supplement: Figure 2—figure supplement 3—source data 3. [file elife-49064-fig2-figsupp3-data3.docx]

Statistical comparisons between WT sibling and *has/apkc* mut Tall cells

**Mann-Whitney Rank Sum Test**

**For Apical Perimeter as shown in Figure 2- figure supplement 3 C1**

**Normality Test (Shapiro-Wilk):**  Failed (P < 0.050)

**Group N Missing Median 25% 75%**

aPKC mut 63 0 66.042 58.781 72.267

aPKC sib 28 0 74.037 64.724 82.985

Mann-Whitney U Statistic= 518.000

T = 1652.000 n(small)= 28 n(big)= 63 (P = 0.002)

The difference in the median values between the two groups is greater than would be expected by chance; there is a statistically significant difference (P = 0.002)

**For Percent cells showing Abnormal Distribution as shown in Figure 2- figure supplement 3 C3**

| **Genotype** | **Distribution** | **Count** | **total** | **Percentage** |
| --- | --- | --- | --- | --- |
| **aPKC sib** | Abnormal | 4 | 28 | 14.28571 |
| **aPKC sib** | Normal | 24 | 28 | 85.71429 |
| **aPKC mut** | Abnormal | 13 | 63 | 20.63492 |
| **aPKC mut** | Normal | 50 | 63 | 79.36508 |
