# Supplementary material for: Stepwise polarisation of developing bilayered epidermis is mediated by aPKC and E-cadherin in zebrafish
Source: eLife. 2020 Jan 22;9:e49064. doi: 10.7554/eLife.49064 (PMC6975926; doi:10.7554/eLife.49064)
Supplement: Figure 2—figure supplement 3—source data 4. [file elife-49064-fig2-figsupp3-data4.docx]

Statistical comparisons between WT sibling and *has/apkc* mut Medium/Short cells

**Mann-Whitney Rank Sum Test**

**For Apical Perimeter as shown in Figure 2- figure supplement 3 D1**

**Normality Test (Shapiro-Wilk):**  Failed (P < 0.050)

**Group N Missing Median 25% 75%**

aPKC mut 82 0 70.707 64.437 75.452

aPKC sib 78 0 80.685 75.850 88.475

Mann-Whitney U Statistic= 1253.000

T = 8224.000 n(small)= 78 n(big)= 82 (P = <0.001)

The difference in the median values between the two groups is greater than would be expected by chance; there is a statistically significant difference (P = <0.001)

**For Percent cells showing abnormal distribution as shown in Figure 2- figure supplement 3 D3**

| **Genotype** | **Distribution** | **Count** | **total** | **Percentage** |
| --- | --- | --- | --- | --- |
| **aPKC sib** | Abnormal | 10 | 96 | 10.41667 |
| **aPKC sib** | Normal | 86 | 96 | 89.58333 |
| **aPKC mut** | Abnormal | 25 | 84 | 29.7619 |
| **aPKC mut** | Normal | 59 | 84 | 70.2381 |
