# Supplementary material for: Stepwise polarisation of developing bilayered epidermis is mediated by aPKC and E-cadherin in zebrafish
Source: eLife. 2020 Jan 22;9:e49064. doi: 10.7554/eLife.49064 (PMC6975926; doi:10.7554/eLife.49064)
Supplement: Figure 2—figure supplement 4—source data 2. [file elife-49064-fig2-figsupp4-data2.docx]

Statistical comparisons between WT sibling and *has/apkc* mut cells

**Kruskal-Wallis One Way Analysis of Variance on Ranks**

**For Height of the Cell as shown in Figure 2- figure supplement 4- B**

**Normality Test (Shapiro-Wilk):**  Failed (P < 0.050)

**Group N Missing Median 25% 75%**

apkc_sibling_CAAX 62 0 4.940 4.085 8.740

apkc_mutant_CAAX 65 0 6.080 4.940 10.830

apkc_sibling_Ecad-mCh 115 0 5.320 4.560 6.080

apkc_mutant_Ecad-mCh 125 0 5.320 4.560 6.080

H = 14.544 with 3 degrees of freedom. (P = 0.002)

The differences in the median values among the treatment groups are greater than would be expected by chance; there is a statistically significant difference (P = 0.002)

To isolate the group or groups that differ from the others use a multiple comparison procedure.

All Pairwise Multiple Comparison Procedures (Dunn's Method) :

**Comparison Diff of Ranks Q P P<0.050**

apkc_mutant vs apkc_sibling 60.039 3.188 0.009 Yes

apkc_mutant vs apkc_mutant_E 55.082 3.395 0.004 Yes

apkc_mutant vs apkc_sibling_ 50.927 3.093 0.012 Yes

apkc_sibling_ vs apkc_sibling 9.112 0.545 1.000 No

apkc_sibling_ vs apkc_mutant_E 4.155 0.303 1.000 Do Not Test

apkc_mutant_E vs apkc_sibling 4.957 0.301 1.000 Do Not Test

Note: The multiple comparisons on ranks do not include an adjustment for ties.

**For Apical Perimeter as shown in Figure 2- figure supplement 4 C**

**Mann-Whitney Rank Sum Test**

**Normality Test (Shapiro-Wilk):**  Failed (P < 0.050)

**Group N Missing Median 25% 75%**

aPKC sib 115 0 81.529 74.678 89.437

aPKC mut 125 0 70.459 64.044 80.207

Mann-Whitney U Statistic= 4131.000

T = 16914.000 n(small)= 115 n(big)= 125 (P = <0.001)

The difference in the median values between the two groups is greater than would be expected by chance; there is a statistically significant difference (P = <0.001)

**For Percent cells showing Abnormal Distribution as shown in Figure 2- figure supplement 4 E**

| **Genotype** | **Distribution** | **Count** | **total** | **Percentage** |
| --- | --- | --- | --- | --- |
| **aPKC sib** | Abnormal | 14 | 115 | 12.17391 |
| **aPKC sib** | Normal | 101 | 115 | 87.82609 |
| **aPKC mut** | Abnormal | 40 | 125 | 32 |
| **aPKC mut** | Normal | 85 | 125 | 68 |
