# Supplementary material for: Stepwise polarisation of developing bilayered epidermis is mediated by aPKC and E-cadherin in zebrafish
Source: eLife. 2020 Jan 22;9:e49064. doi: 10.7554/eLife.49064 (PMC6975926; doi:10.7554/eLife.49064)
Supplement: Figure 3—source data 3. [file elife-49064-fig3-data3.docx]

Statistical comparisons between WT sibling and *pen/lgl2* mut periderm

**Mann-Whitney Rank Sum Test**

**For Height of cell as shown in Figure 3 A2**

**Normality Test (Shapiro-Wilk):**  Passed (P = 0.180)

**Equal Variance Test:** Passed (P = 0.483)

**Group N Missing Median 25% 75%**

Penner sib 113 0 1.960 1.400 2.240

Penner mut 108 0 2.240 1.680 2.520

Mann-Whitney U Statistic= 5183.000

T = 12907.000 n(small)= 108 n(big)= 113 (P = 0.051)

The difference in the median values between the two groups is not great enough to exclude the possibility that the difference is due to random sampling variability; there is not a statistically significant difference (P = 0.051)

**Mann-Whitney Rank Sum Test**

**For Apical Perimeter as shown in Figure 3 A3**

**Normality Test (Shapiro-Wilk):**  Failed (P < 0.050)

**Group N Missing Median 25% 75%**

Penner sib 113 0 78.605 73.286 85.887

Penner mut 108 0 80.612 72.947 87.425

Mann-Whitney U Statistic= 5903.000

T = 12187.000 n(small)= 108 n(big)= 113 (P = 0.676)

The difference in the median values between the two groups is not great enough to exclude the possibility that the difference is due to random sampling variability; there is not a statistically significant difference (P = 0.676)
