# Supplementary material for: Stepwise polarisation of developing bilayered epidermis is mediated by aPKC and E-cadherin in zebrafish
Source: eLife. 2020 Jan 22;9:e49064. doi: 10.7554/eLife.49064 (PMC6975926; doi:10.7554/eLife.49064)
Supplement: Figure 3—source data 4. [file elife-49064-fig3-data4.docx]

Statistical comparisons between WT sibling and *pen/lgl2* mut basal epidermis

**Mann-Whitney Rank Sum Test**

**For Height of cell as shown in Figure 3 B2**

**Normality Test (Shapiro-Wilk):**  Failed (P < 0.050)

**Group N Missing Median 25% 75%**

Penner sib 127 0 2.240 1.680 2.520

Penner mut 106 0 2.240 1.680 2.800

Mann-Whitney U Statistic= 6412.500

T = 12720.500 n(small)= 106 n(big)= 127 (P = 0.532)

The difference in the median values between the two groups is not great enough to exclude the possibility that the difference is due to random sampling variability; there is not a statistically significant difference (P = 0.532)

**Mann-Whitney Rank Sum Test**

**For Apical Perimeter as shown in Figure 3 B3**

**Normality Test (Shapiro-Wilk):**  Passed (P = 0.051)

**Equal Variance Test:** Passed (P = 0.725)

**Group N Missing Median 25% 75%**

Penner sib 127 0 61.299 55.814 66.510

Penner mut 106 0 65.105 58.848 69.394

Mann-Whitney U Statistic= 5308.500

T = 13824.500 n(small)= 106 n(big)= 127 (P = 0.006)

The difference in the median values between the two groups is greater than would be expected by chance; there is a statistically significant difference (P = 0.006)
