# Supplementary material for: Stepwise polarisation of developing bilayered epidermis is mediated by aPKC and E-cadherin in zebrafish
Source: eLife. 2020 Jan 22;9:e49064. doi: 10.7554/eLife.49064 (PMC6975926; doi:10.7554/eLife.49064)
Supplement: Figure 4—source data 5. [file elife-49064-fig4-data5.docx]

Statistical comparisons for E-cadherin levels between Ctrl MO and Ecad MO at boundary Figure 4

**Color code:**

Comparisons showing high statisitcal significance

Comparisons showing weak statistical significance (p < 0.08), possibly arising due to large variations, may be biologically relevant

**Kruskal-Wallis One Way Analysis of Variance on Ranks**

**For Ecad at Normalized Cell Height at 0.1 as shown in Figure 4 B**

**Normality Test (Shapiro-Wilk):**  Passed (P = 0.050)

**Equal Variance Test (Brown-Forsythe):** Failed (P < 0.050)

**Group N Missing Median 25% 75%**

Ctrl MO C-C 48 0 1017.290 723.327 1247.242

Ctrl MO N-C 32 0 798.033 687.316 1006.871

Ctrl MO N-N 23 0 962.720 638.358 1165.590

Ecad MO C-C 28 0 336.710 241.709 481.344

Ecad MO N-C 44 0 512.076 385.874 668.145

Ecad MO N-N 22 0 658.635 550.385 805.809

H = 91.990 with 5 degrees of freedom. (P = <0.001)

The differences in the median values among the treatment groups are greater than would be expected by chance; there is a statistically significant difference (P = <0.001)

To isolate the group or groups that differ from the others use a multiple comparison procedure.

All Pairwise Multiple Comparison Procedures (Dunn's Method) :

**Comparison Diff of Ranks Q P P<0.050**

Ctrl MO C-C vs Ecad MO C-C 104.920 7.739 <0.001 Yes

Ctrl MO C-C vs Ecad MO N-C 78.017 6.556 <0.001 Yes

Ctrl MO C-C vs Ecad MO N-N 50.676 3.452 0.008 Yes

Ctrl MO C-C vs Ctrl MO N-C 16.500 1.268 1.000 No

Ctrl MO C-C vs Ctrl MO N-N 9.726 0.673 1.000 Do Not Test

Ctrl MO N-N vs Ecad MO C-C 95.194 5.933 <0.001 Yes

Ctrl MO N-N vs Ecad MO N-C 68.292 4.655 <0.001 Yes

Ctrl MO N-N vs Ecad MO N-N 40.951 2.409 0.240 No

Ctrl MO N-N vs Ctrl MO N-C 6.774 0.435 1.000 Do Not Test

Ctrl MO N-C vs Ecad MO C-C 88.420 5.993 <0.001 Yes

Ctrl MO N-C vs Ecad MO N-C 61.517 4.644 <0.001 Yes

Ctrl MO N-C vs Ecad MO N-N 34.176 2.164 0.457 Do Not Test

Ecad MO N-N vs Ecad MO C-C 54.244 3.339 0.013 Yes

Ecad MO N-N vs Ecad MO N-C 27.341 1.837 0.994 No

Ecad MO N-C vs Ecad MO C-C 26.903 1.952 0.764 No

Note: The multiple comparisons on ranks do not include an adjustment for ties.

**Kruskal-Wallis One Way Analysis of Variance on Ranks**

**For Ecad at Normalized Cell Height at 0.3 as shown in Figure 4 B**

**Normality Test (Shapiro-Wilk):**  Failed (P < 0.050)

**Group N Missing Median 25% 75%**

Ctrl MO C-C 48 0 1110.032 805.653 1482.525

Ctrl MO N-C 32 0 923.599 804.792 1190.711

Ctrl MO N-N 23 0 1099.045 935.623 1371.601

Ecad MO C-C 28 0 396.801 283.627 533.649

Ecad MO N-C 44 0 526.629 450.092 705.750

Ecad MO N-N 22 0 743.609 561.749 934.391

H = 98.866 with 5 degrees of freedom. (P = <0.001)

The differences in the median values among the treatment groups are greater than would be expected by chance; there is a statistically significant difference (P = <0.001)

To isolate the group or groups that differ from the others use a multiple comparison procedure.

All Pairwise Multiple Comparison Procedures (Dunn's Method) :

**Comparison Diff of Ranks Q P P<0.050**

Ctrl MO C-C vs Ecad MO C-C 104.994 7.744 <0.001 Yes

Ctrl MO C-C vs Ecad MO N-C 79.367 6.670 <0.001 Yes

Ctrl MO C-C vs Ecad MO N-N 52.004 3.543 0.006 Yes

Ctrl MO C-C vs Ctrl MO N-C 14.208 1.092 1.000 No

Ctrl MO C-C vs Ctrl MO N-N 1.654 0.114 1.000 Do Not Test

Ctrl MO N-N vs Ecad MO C-C 103.340 6.441 <0.001 Yes

Ctrl MO N-N vs Ecad MO N-C 77.713 5.298 <0.001 Yes

Ctrl MO N-N vs Ecad MO N-N 50.350 2.961 0.046 Yes

Ctrl MO N-N vs Ctrl MO N-C 12.554 0.806 1.000 Do Not Test

Ctrl MO N-C vs Ecad MO C-C 90.786 6.153 <0.001 Yes

Ctrl MO N-C vs Ecad MO N-C 65.159 4.919 <0.001 Yes

Ctrl MO N-C vs Ecad MO N-N 37.795 2.394 0.250 No

Ecad MO N-N vs Ecad MO C-C 52.990 3.262 0.017 Yes

Ecad MO N-N vs Ecad MO N-C 27.364 1.838 0.991 No

Ecad MO N-C vs Ecad MO C-C 25.627 1.859 0.945 No

Note: The multiple comparisons on ranks do not include an adjustment for ties.

**Kruskal-Wallis One Way Analysis of Variance on Ranks**

**For Ecad at Normalized Cell Height at 0.5 as shown in Figure 4 B**

**Normality Test (Shapiro-Wilk):**  Passed (P = 0.084)

**Equal Variance Test (Brown-Forsythe):** Failed (P < 0.050)

**Group N Missing Median 25% 75%**

Ctrl MO C-C 48 0 1317.582 927.478 1662.342

Ctrl MO N-C 32 0 1178.503 973.110 1385.054

Ctrl MO N-N 23 0 1309.562 1126.020 1529.699

Ecad MO C-C 28 0 461.294 322.535 664.526

Ecad MO N-C 44 0 629.546 487.380 775.891

Ecad MO N-N 22 0 842.848 651.071 1023.067

H = 104.314 with 5 degrees of freedom. (P = <0.001)

The differences in the median values among the treatment groups are greater than would be expected by chance; there is a statistically significant difference (P = <0.001)

To isolate the group or groups that differ from the others use a multiple comparison procedure.

All Pairwise Multiple Comparison Procedures (Dunn's Method) :

**Comparison Diff of Ranks Q P P<0.050**

Ctrl MO N-N vs Ecad MO C-C 105.489 6.575 <0.001 Yes

Ctrl MO N-N vs Ecad MO N-C 81.103 5.529 <0.001 Yes

Ctrl MO N-N vs Ecad MO N-N 53.421 3.142 0.025 Yes

Ctrl MO N-N vs Ctrl MO N-C 8.208 0.527 1.000 No

Ctrl MO N-N vs Ctrl MO C-C 1.364 0.0943 1.000 Do Not Test

Ctrl MO C-C vs Ecad MO C-C 104.125 7.680 <0.001 Yes

Ctrl MO C-C vs Ecad MO N-C 79.739 6.701 <0.001 Yes

Ctrl MO C-C vs Ecad MO N-N 52.057 3.546 0.006 Yes

Ctrl MO C-C vs Ctrl MO N-C 6.844 0.526 1.000 Do Not Test

Ctrl MO N-C vs Ecad MO C-C 97.281 6.594 <0.001 Yes

Ctrl MO N-C vs Ecad MO N-C 72.895 5.503 <0.001 Yes

Ctrl MO N-C vs Ecad MO N-N 45.213 2.863 0.063 No

Ecad MO N-N vs Ecad MO C-C 52.068 3.206 0.020 Yes

Ecad MO N-N vs Ecad MO N-C 27.682 1.859 0.944 No

Ecad MO N-C vs Ecad MO C-C 24.386 1.769 1.000 No

Note: The multiple comparisons on ranks do not include an adjustment for ties.

**Kruskal-Wallis One Way Analysis of Variance on Ranks**

**For Ecad at Normalized Cell Height at 0.7 as shown in Figure 4 B**

**Normality Test (Shapiro-Wilk):**  Passed (P = 0.258)

**Equal Variance Test (Brown-Forsythe):** Failed (P < 0.050)

**Group N Missing Median 25% 75%**

Ctrl MO C-C 48 0 1538.450 1082.953 1900.675

Ctrl MO N-C 32 0 1319.106 1091.728 1507.019

Ctrl MO N-N 23 0 1534.485 1247.447 1683.301

Ecad MO C-C 28 0 550.241 356.078 672.004

Ecad MO N-C 44 0 690.145 529.850 976.305

Ecad MO N-N 22 0 936.956 670.847 1059.482

H = 116.393 with 5 degrees of freedom. (P = <0.001)

The differences in the median values among the treatment groups are greater than would be expected by chance; there is a statistically significant difference (P = <0.001)

To isolate the group or groups that differ from the others use a multiple comparison procedure.

All Pairwise Multiple Comparison Procedures (Dunn's Method) :

**Comparison Diff of Ranks Q P P<0.050**

Ctrl MO N-N vs Ecad MO C-C 115.946 7.227 <0.001 Yes

Ctrl MO N-N vs Ecad MO N-C 87.650 5.975 <0.001 Yes

Ctrl MO N-N vs Ecad MO N-N 67.696 3.982 0.001 Yes

Ctrl MO N-N vs Ctrl MO N-C 17.727 1.137 1.000 No

Ctrl MO N-N vs Ctrl MO C-C 4.925 0.341 1.000 Do Not Test

Ctrl MO C-C vs Ecad MO C-C 111.021 8.189 <0.001 Yes

Ctrl MO C-C vs Ecad MO N-C 82.725 6.952 <0.001 Yes

Ctrl MO C-C vs Ecad MO N-N 62.771 4.276 <0.001 Yes

Ctrl MO C-C vs Ctrl MO N-C 12.802 0.984 1.000 Do Not Test

Ctrl MO N-C vs Ecad MO C-C 98.219 6.657 <0.001 Yes

Ctrl MO N-C vs Ecad MO N-C 69.923 5.279 <0.001 Yes

Ctrl MO N-C vs Ecad MO N-N 49.969 3.165 0.023 Yes

Ecad MO N-N vs Ecad MO C-C 48.250 2.970 0.045 Yes

Ecad MO N-N vs Ecad MO N-C 19.955 1.340 1.000 No

Ecad MO N-C vs Ecad MO C-C 28.295 2.053 0.601 No

Note: The multiple comparisons on ranks do not include an adjustment for ties.

**Kruskal-Wallis One Way Analysis of Variance on Ranks**

**For Ecad at Normalized Cell Height at 0.9 as shown in Figure 4 B**

**Normality Test (Shapiro-Wilk):**  Failed (P < 0.050)

**Group N Missing Median 25% 75%**

Ctrl MO C-C 48 0 1479.190 1119.413 1712.720

Ctrl MO N-C 32 0 1192.028 1001.555 1299.672

Ctrl MO N-N 23 0 1358.189 1166.497 1664.694

Ecad MO C-C 28 0 520.711 373.077 647.509

Ecad MO N-C 44 0 687.892 501.333 963.647

Ecad MO N-N 22 0 829.645 633.387 1052.692

H = 109.504 with 5 degrees of freedom. (P = <0.001)

The differences in the median values among the treatment groups are greater than would be expected by chance; there is a statistically significant difference (P = <0.001)

To isolate the group or groups that differ from the others use a multiple comparison procedure.

All Pairwise Multiple Comparison Procedures (Dunn's Method) :

**Comparison Diff of Ranks Q P P<0.050**

Ctrl MO C-C vs Ecad MO C-C 111.997 8.261 <0.001 Yes

Ctrl MO C-C vs Ecad MO N-C 83.127 6.986 <0.001 Yes

Ctrl MO C-C vs Ecad MO N-N 66.354 4.520 <0.001 Yes

Ctrl MO C-C vs Ctrl MO N-C 23.729 1.824 1.000 No

Ctrl MO C-C vs Ctrl MO N-N 0.898 0.0621 1.000 Do Not Test

Ctrl MO N-N vs Ecad MO C-C 111.099 6.925 <0.001 Yes

Ctrl MO N-N vs Ecad MO N-C 82.229 5.605 <0.001 Yes

Ctrl MO N-N vs Ecad MO N-N 65.457 3.850 0.002 Yes

Ctrl MO N-N vs Ctrl MO N-C 22.832 1.465 1.000 Do Not Test

Ctrl MO N-C vs Ecad MO C-C 88.268 5.983 <0.001 Yes

Ctrl MO N-C vs Ecad MO N-C 59.398 4.484 <0.001 Yes

Ctrl MO N-C vs Ecad MO N-N 42.625 2.699 0.104 No

Ecad MO N-N vs Ecad MO C-C 45.643 2.810 0.074 No

Ecad MO N-N vs Ecad MO N-C 16.773 1.127 1.000 Do Not Test

Ecad MO N-C vs Ecad MO C-C 28.870 2.095 0.543 Do Not Test

Note: The multiple comparisons on ranks do not include an adjustment for ties.

**Kruskal-Wallis One Way Analysis of Variance on Ranks**

**For Ecad at Normalized Cell Height at 0.1 as shown in Figure 4 C**

**Normality Test (Shapiro-Wilk):**  Failed (P < 0.050)

**Group N Missing Median 25% 75%**

Ctrl MO CO 48 0 1780.977 1450.368 2168.266

Ctrl MO PO 24 0 1773.100 1402.739 2098.379

Ctrl MO NO 17 0 1767.783 1533.939 2182.437

Ecad MO CO 37 0 781.192 503.487 1357.103

Ecad MO PO 12 0 920.682 620.794 1221.063

Ecad MO NO 13 0 1209.887 1044.032 1777.351

H = 64.564 with 5 degrees of freedom. (P = <0.001)

The differences in the median values among the treatment groups are greater than would be expected by chance; there is a statistically significant difference (P = <0.001)

To isolate the group or groups that differ from the others use a multiple comparison procedure.

All Pairwise Multiple Comparison Procedures (Dunn's Method) :

**Comparison Diff of Ranks Q P P<0.050**

Ctrl MO NO vs Ecad MO PO 67.132 4.071 <0.001 Yes

Ctrl MO NO vs Ecad MO CO 64.045 4.998 <0.001 Yes

Ctrl MO NO vs Ecad MO NO 36.959 2.294 0.327 No

Ctrl MO NO vs Ctrl MO PO 5.591 0.403 1.000 Do Not Test

Ctrl MO NO vs Ctrl MO CO 2.466 0.200 1.000 Do Not Test

Ctrl MO CO vs Ecad MO PO 64.667 4.581 <0.001 Yes

Ctrl MO CO vs Ecad MO CO 61.579 6.436 <0.001 Yes

Ctrl MO CO vs Ecad MO NO 34.494 2.523 0.175 Do Not Test

Ctrl MO CO vs Ctrl MO PO 3.125 0.286 1.000 Do Not Test

Ctrl MO PO vs Ecad MO PO 61.542 3.980 0.001 Yes

Ctrl MO PO vs Ecad MO CO 58.454 5.100 <0.001 Yes

Ctrl MO PO vs Ecad MO NO 31.369 2.083 0.559 Do Not Test

Ecad MO NO vs Ecad MO PO 30.173 1.723 1.000 No

Ecad MO NO vs Ecad MO CO 27.085 1.921 0.821 Do Not Test

Ecad MO CO vs Ecad MO PO 3.088 0.213 1.000 Do Not Test

Note: The multiple comparisons on ranks do not include an adjustment for ties.

**Kruskal-Wallis One Way Analysis of Variance on Ranks**

**For Ecad at Normalized Cell Height at 0.3 as shown in Figure 4 C**

**Normality Test (Shapiro-Wilk):**  Failed (P < 0.050)

**Group N Missing Median 25% 75%**

Ctrl MO CO 48 0 1606.859 1306.593 1889.562

Ctrl MO PO 24 0 1333.500 1264.613 1764.986

Ctrl MO NO 17 0 1546.397 1176.228 1994.708

Ecad MO CO 37 0 758.285 461.818 1261.195

Ecad MO PO 12 0 737.716 439.987 961.781

Ecad MO NO 13 0 980.149 735.848 1270.243

H = 53.112 with 5 degrees of freedom. (P = <0.001)

The differences in the median values among the treatment groups are greater than would be expected by chance; there is a statistically significant difference (P = <0.001)

To isolate the group or groups that differ from the others use a multiple comparison procedure.

All Pairwise Multiple Comparison Procedures (Dunn's Method) :

**Comparison Diff of Ranks Q P P<0.050**

Ctrl MO CO vs Ecad MO PO 65.375 4.632 <0.001 Yes

Ctrl MO CO vs Ecad MO CO 54.465 5.693 <0.001 Yes

Ctrl MO CO vs Ecad MO NO 42.324 3.095 0.030 Yes

Ctrl MO CO vs Ctrl MO PO 9.417 0.861 1.000 No

Ctrl MO CO vs Ctrl MO NO 0.238 0.0193 1.000 Do Not Test

Ctrl MO NO vs Ecad MO PO 65.137 3.950 0.001 Yes

Ctrl MO NO vs Ecad MO CO 54.227 4.232 <0.001 Yes

Ctrl MO NO vs Ecad MO NO 42.086 2.612 0.135 No

Ctrl MO NO vs Ctrl MO PO 9.179 0.662 1.000 Do Not Test

Ctrl MO PO vs Ecad MO PO 55.958 3.619 0.004 Yes

Ctrl MO PO vs Ecad MO CO 45.048 3.930 0.001 Yes

Ctrl MO PO vs Ecad MO NO 32.907 2.185 0.433 Do Not Test

Ecad MO NO vs Ecad MO PO 23.051 1.317 1.000 No

Ecad MO NO vs Ecad MO CO 12.141 0.861 1.000 Do Not Test

Ecad MO CO vs Ecad MO PO 10.910 0.751 1.000 Do Not Test

Note: The multiple comparisons on ranks do not include an adjustment for ties.

**Kruskal-Wallis One Way Analysis of Variance on Ranks**

**For Ecad at Normalized Cell Height at 0.5 as shown in Figure 4 C**

**Normality Test (Shapiro-Wilk):**  Failed (P < 0.050)

**Group N Missing Median 25% 75%**

Ctrl MO CO 48 0 1195.161 878.701 1459.021

Ctrl MO PO 24 0 1014.678 898.614 1415.422

Ctrl MO NO 17 0 1222.532 838.579 1472.173

Ecad MO CO 37 0 666.449 374.590 984.062

Ecad MO PO 12 0 565.086 335.736 800.158

Ecad MO NO 13 0 526.525 440.070 823.863

H = 41.605 with 5 degrees of freedom. (P = <0.001)

The differences in the median values among the treatment groups are greater than would be expected by chance; there is a statistically significant difference (P = <0.001)

To isolate the group or groups that differ from the others use a multiple comparison procedure.

All Pairwise Multiple Comparison Procedures (Dunn's Method) :

**Comparison Diff of Ranks Q P P<0.050**

Ctrl MO CO vs Ecad MO PO 56.063 3.972 0.001 Yes

Ctrl MO CO vs Ecad MO NO 51.082 3.736 0.003 Yes

Ctrl MO CO vs Ecad MO CO 43.421 4.538 <0.001 Yes

Ctrl MO CO vs Ctrl MO PO 4.396 0.402 1.000 No

Ctrl MO CO vs Ctrl MO NO 1.077 0.0873 1.000 Do Not Test

Ctrl MO NO vs Ecad MO PO 54.985 3.335 0.013 Yes

Ctrl MO NO vs Ecad MO NO 50.005 3.103 0.029 Yes

Ctrl MO NO vs Ecad MO CO 42.343 3.304 0.014 Yes

Ctrl MO NO vs Ctrl MO PO 3.319 0.239 1.000 Do Not Test

Ctrl MO PO vs Ecad MO PO 51.667 3.341 0.013 Yes

Ctrl MO PO vs Ecad MO NO 46.686 3.100 0.029 Yes

Ctrl MO PO vs Ecad MO CO 39.025 3.405 0.010 Yes

Ecad MO CO vs Ecad MO PO 12.642 0.870 1.000 No

Ecad MO CO vs Ecad MO NO 7.661 0.543 1.000 Do Not Test

Ecad MO NO vs Ecad MO PO 4.981 0.284 1.000 Do Not Test

Note: The multiple comparisons on ranks do not include an adjustment for ties.

**Kruskal-Wallis One Way Analysis of Variance on Ranks**

**For Ecad at Normalized Cell Height at 0.7 as shown in Figure 4 C**

**Normality Test (Shapiro-Wilk):**  Failed (P < 0.050)

**Group N Missing Median 25% 75%**

Ctrl MO CO 48 0 791.137 673.056 936.208

Ctrl MO PO 24 0 720.525 653.036 1069.395

Ctrl MO NO 17 0 776.867 611.724 1066.868

Ecad MO CO 37 0 494.629 320.224 789.568

Ecad MO PO 12 0 439.768 291.727 690.866

Ecad MO NO 13 0 295.176 257.036 588.023

H = 39.856 with 5 degrees of freedom. (P = <0.001)

The differences in the median values among the treatment groups are greater than would be expected by chance; there is a statistically significant difference (P = <0.001)

To isolate the group or groups that differ from the others use a multiple comparison procedure.

All Pairwise Multiple Comparison Procedures (Dunn's Method) :

**Comparison Diff of Ranks Q P P<0.050**

Ctrl MO CO vs Ecad MO NO 60.563 4.429 <0.001 Yes

Ctrl MO CO vs Ecad MO PO 50.229 3.559 0.006 Yes

Ctrl MO CO vs Ecad MO CO 38.779 4.053 <0.001 Yes

Ctrl MO CO vs Ctrl MO NO 3.857 0.312 1.000 No

Ctrl MO CO vs Ctrl MO PO 2.646 0.242 1.000 Do Not Test

Ctrl MO PO vs Ecad MO NO 57.917 3.846 0.002 Yes

Ctrl MO PO vs Ecad MO PO 47.583 3.077 0.031 Yes

Ctrl MO PO vs Ecad MO CO 36.133 3.152 0.024 Yes

Ctrl MO PO vs Ctrl MO NO 1.211 0.0873 1.000 Do Not Test

Ctrl MO NO vs Ecad MO NO 56.706 3.519 0.006 Yes

Ctrl MO NO vs Ecad MO PO 46.373 2.812 0.074 No

Ctrl MO NO vs Ecad MO CO 34.922 2.725 0.096 Do Not Test

Ecad MO CO vs Ecad MO NO 21.784 1.545 1.000 No

Ecad MO CO vs Ecad MO PO 11.450 0.788 1.000 Do Not Test

Ecad MO PO vs Ecad MO NO 10.333 0.590 1.000 Do Not Test

Note: The multiple comparisons on ranks do not include an adjustment for ties.

**Kruskal-Wallis One Way Analysis of Variance on Ranks**

**For Ecad at Normalized Cell Height at 0.9 as shown in Figure 4 C**

**Normality Test (Shapiro-Wilk):**  Failed (P < 0.050)

**Group N Missing Median 25% 75%**

Ctrl MO CO 48 0 569.368 473.826 668.602

Ctrl MO PO 24 0 582.605 421.589 770.635

Ctrl MO NO 17 0 491.349 309.927 715.671

Ecad MO CO 37 0 364.866 271.194 533.396

Ecad MO PO 12 0 329.442 237.535 550.421

Ecad MO NO 13 0 209.592 150.712 380.544

H = 39.975 with 5 degrees of freedom. (P = <0.001)

The differences in the median values among the treatment groups are greater than would be expected by chance; there is a statistically significant difference (P = <0.001)

To isolate the group or groups that differ from the others use a multiple comparison procedure.

All Pairwise Multiple Comparison Procedures (Dunn's Method) :

**Comparison Diff of Ranks Q P P<0.050**

Ctrl MO PO vs Ecad MO NO 66.904 4.442 <0.001 Yes

Ctrl MO PO vs Ecad MO PO 47.917 3.099 0.029 Yes

Ctrl MO PO vs Ecad MO CO 37.345 3.258 0.017 Yes

Ctrl MO PO vs Ctrl MO NO 15.515 1.119 1.000 No

Ctrl MO PO vs Ctrl MO CO 0.896 0.0819 1.000 Do Not Test

Ctrl MO CO vs Ecad MO NO 66.008 4.827 <0.001 Yes

Ctrl MO CO vs Ecad MO PO 47.021 3.331 0.013 Yes

Ctrl MO CO vs Ecad MO CO 36.449 3.810 0.002 Yes

Ctrl MO CO vs Ctrl MO NO 14.619 1.184 1.000 Do Not Test

Ctrl MO NO vs Ecad MO NO 51.389 3.189 0.021 Yes

Ctrl MO NO vs Ecad MO PO 32.402 1.965 0.741 No

Ctrl MO NO vs Ecad MO CO 21.830 1.704 1.000 Do Not Test

Ecad MO CO vs Ecad MO NO 29.559 2.096 0.541 No

Ecad MO CO vs Ecad MO PO 10.572 0.728 1.000 Do Not Test

Ecad MO PO vs Ecad MO NO 18.987 1.085 1.000 Do Not Test

Note: The multiple comparisons on ranks do not include an adjustment for ties.

**Kruskal-Wallis One Way Analysis of Variance on Ranks**

**For Ecad at Normalized Cell Height at 0.1 as shown in Figure 4 E**

**Normality Test (Shapiro-Wilk):**  Failed (P < 0.050)

**Group N Missing Median 25% 75%**

Ctrl MO CO 41 0 875.523 715.646 1120.216

Ctrl MO PO 21 0 1032.002 861.730 1198.734

Ctrl MO NO 40 0 864.044 672.997 1060.765

Ecad MO CO 69 0 952.541 749.348 1160.294

Ecad MO PO 20 0 762.616 615.022 974.912

Ecad MO NO 36 0 796.059 665.001 1106.692

H = 9.682 with 5 degrees of freedom. (P = 0.085)

The differences in the median values among the treatment groups are not great enough to exclude the possibility that the difference is due to random sampling variability; there is not a statistically significant difference (P = 0.085)

All Pairwise Multiple Comparison Procedures (Dunn's Method) :

**Comparison Diff of Ranks Q P P<0.050**

Ctrl MO PO vs Ecad MO PO 58.045 2.829 0.070 No

Ctrl MO PO vs Ecad MO NO 37.762 2.094 0.544 Do Not Test

Ctrl MO PO vs Ctrl MO NO 35.620 2.013 0.662 Do Not Test

Ctrl MO PO vs Ctrl MO CO 25.705 1.459 1.000 Do Not Test

Ctrl MO PO vs Ecad MO CO 23.269 1.422 1.000 Do Not Test

Ecad MO CO vs Ecad MO PO 34.776 2.085 0.556 Do Not Test

Ecad MO CO vs Ecad MO NO 14.493 1.073 1.000 Do Not Test

Ecad MO CO vs Ctrl MO NO 12.351 0.946 1.000 Do Not Test

Ecad MO CO vs Ctrl MO CO 2.436 0.188 1.000 Do Not Test

Ctrl MO CO vs Ecad MO PO 32.340 1.805 1.000 Do Not Test

Ctrl MO CO vs Ecad MO NO 12.057 0.804 1.000 Do Not Test

Ctrl MO CO vs Ctrl MO NO 9.915 0.679 1.000 Do Not Test

Ctrl MO NO vs Ecad MO PO 22.425 1.247 1.000 Do Not Test

Ctrl MO NO vs Ecad MO NO 2.142 0.142 1.000 Do Not Test

Ecad MO NO vs Ecad MO PO 20.283 1.107 1.000 Do Not Test

Note: The multiple comparisons on ranks do not include an adjustment for ties.

**Kruskal-Wallis One Way Analysis of Variance on Ranks**

**For Ecad at Normalized Cell Height at 0.3 as shown in Figure 4 E**

**Normality Test (Shapiro-Wilk):**  Failed (P < 0.050)

**Group N Missing Median 25% 75%**

Ctrl MO CO 41 0 1067.899 892.596 1252.812

Ctrl MO PO 21 0 1074.190 939.725 1231.022

Ctrl MO NO 40 0 1015.113 844.327 1274.064

Ecad MO CO 69 0 1000.826 794.565 1213.605

Ecad MO PO 20 0 846.862 719.340 1147.063

Ecad MO NO 36 0 905.966 743.146 1164.286

H = 9.651 with 5 degrees of freedom. (P = 0.086)

The differences in the median values among the treatment groups are not great enough to exclude the possibility that the difference is due to random sampling variability; there is not a statistically significant difference (P = 0.086)

All Pairwise Multiple Comparison Procedures (Dunn's Method) :

**Comparison Diff of Ranks Q P P<0.050**

Ctrl MO PO vs Ecad MO PO 50.083 2.441 0.220 No

Ctrl MO PO vs Ecad MO NO 37.333 2.070 0.576 Do Not Test

Ctrl MO PO vs Ecad MO CO 26.029 1.590 1.000 Do Not Test

Ctrl MO PO vs Ctrl MO NO 18.833 1.064 1.000 Do Not Test

Ctrl MO PO vs Ctrl MO CO 9.797 0.556 1.000 Do Not Test

Ctrl MO CO vs Ecad MO PO 40.287 2.249 0.368 Do Not Test

Ctrl MO CO vs Ecad MO NO 27.537 1.836 0.996 Do Not Test

Ctrl MO CO vs Ecad MO CO 16.232 1.253 1.000 Do Not Test

Ctrl MO CO vs Ctrl MO NO 9.037 0.619 1.000 Do Not Test

Ctrl MO NO vs Ecad MO PO 31.250 1.738 1.000 Do Not Test

Ctrl MO NO vs Ecad MO NO 18.500 1.226 1.000 Do Not Test

Ctrl MO NO vs Ecad MO CO 7.196 0.551 1.000 Do Not Test

Ecad MO CO vs Ecad MO PO 24.054 1.442 1.000 Do Not Test

Ecad MO CO vs Ecad MO NO 11.304 0.837 1.000 Do Not Test

Ecad MO NO vs Ecad MO PO 12.750 0.696 1.000 Do Not Test

Note: The multiple comparisons on ranks do not include an adjustment for ties.

**Kruskal-Wallis One Way Analysis of Variance on Ranks**

**For Ecad at Normalized Cell Height at 0.5 as shown in Figure 4 E**

**Normality Test (Shapiro-Wilk):**  Failed (P < 0.050)

**Group N Missing Median 25% 75%**

Ctrl MO CO 41 0 1283.853 1019.330 1607.867

Ctrl MO PO 21 0 1273.148 1073.980 1647.926

Ctrl MO NO 40 0 1161.503 974.792 1455.650

Ecad MO CO 69 0 1025.954 910.769 1317.489

Ecad MO PO 20 0 1006.052 717.507 1260.283

Ecad MO NO 36 0 1032.453 786.525 1226.816

H = 19.114 with 5 degrees of freedom. (P = 0.002)

The differences in the median values among the treatment groups are greater than would be expected by chance; there is a statistically significant difference (P = 0.002)

To isolate the group or groups that differ from the others use a multiple comparison procedure.

All Pairwise Multiple Comparison Procedures (Dunn's Method) :

**Comparison Diff of Ranks Q P P<0.050**

Ctrl MO PO vs Ecad MO NO 50.782 2.816 0.073 No

Ctrl MO PO vs Ecad MO PO 50.160 2.445 0.218 Do Not Test

Ctrl MO PO vs Ecad MO CO 42.867 2.619 0.132 Do Not Test

Ctrl MO PO vs Ctrl MO NO 17.585 0.994 1.000 Do Not Test

Ctrl MO PO vs Ctrl MO CO 6.688 0.379 1.000 Do Not Test

Ctrl MO CO vs Ecad MO NO 44.094 2.940 0.049 Do Not Test

Ctrl MO CO vs Ecad MO PO 43.472 2.427 0.228 Do Not Test

Ctrl MO CO vs Ecad MO CO 36.180 2.794 0.078 Do Not Test

Ctrl MO CO vs Ctrl MO NO 10.897 0.747 1.000 Do Not Test

Ctrl MO NO vs Ecad MO NO 33.197 2.200 0.417 Do Not Test

Ctrl MO NO vs Ecad MO PO 32.575 1.811 1.000 Do Not Test

Ctrl MO NO vs Ecad MO CO 25.283 1.937 0.791 Do Not Test

Ecad MO CO vs Ecad MO NO 7.914 0.586 1.000 Do Not Test

Ecad MO CO vs Ecad MO PO 7.292 0.437 1.000 Do Not Test

Ecad MO PO vs Ecad MO NO 0.622 0.0340 1.000 Do Not Test

Note: The multiple comparisons on ranks do not include an adjustment for ties.

**Kruskal-Wallis One Way Analysis of Variance on Ranks**

**For Ecad at Normalized Cell Height at 0.7 as shown in Figure 4 E**

**Normality Test (Shapiro-Wilk):**  Passed (P = 0.073)

**Equal Variance Test (Brown-Forsythe):** Failed (P < 0.050)

**Group N Missing Median 25% 75%**

Ctrl MO CO 41 0 1521.602 1155.424 2044.535

Ctrl MO PO 21 0 1544.731 1269.642 1817.866

Ctrl MO NO 40 0 1393.784 1155.019 1755.558

Ecad MO CO 69 0 963.982 824.375 1331.884

Ecad MO PO 20 0 983.562 720.891 1154.638

Ecad MO NO 36 0 1033.011 790.059 1214.520

H = 56.699 with 5 degrees of freedom. (P = <0.001)

The differences in the median values among the treatment groups are greater than would be expected by chance; there is a statistically significant difference (P = <0.001)

To isolate the group or groups that differ from the others use a multiple comparison procedure.

All Pairwise Multiple Comparison Procedures (Dunn's Method) :

**Comparison Diff of Ranks Q P P<0.050**

Ctrl MO PO vs Ecad MO PO 81.131 3.954 0.001 Yes

Ctrl MO PO vs Ecad MO NO 73.464 4.074 <0.001 Yes

Ctrl MO PO vs Ecad MO CO 71.685 4.380 <0.001 Yes

Ctrl MO PO vs Ctrl MO NO 15.656 0.885 1.000 No

Ctrl MO PO vs Ctrl MO CO 5.722 0.325 1.000 Do Not Test

Ctrl MO CO vs Ecad MO PO 75.409 4.210 <0.001 Yes

Ctrl MO CO vs Ecad MO NO 67.742 4.516 <0.001 Yes

Ctrl MO CO vs Ecad MO CO 65.963 5.094 <0.001 Yes

Ctrl MO CO vs Ctrl MO NO 9.934 0.681 1.000 Do Not Test

Ctrl MO NO vs Ecad MO PO 65.475 3.640 0.004 Yes

Ctrl MO NO vs Ecad MO NO 57.808 3.832 0.002 Yes

Ctrl MO NO vs Ecad MO CO 56.029 4.293 <0.001 Yes

Ecad MO CO vs Ecad MO PO 9.446 0.566 1.000 No

Ecad MO CO vs Ecad MO NO 1.779 0.132 1.000 Do Not Test

Ecad MO NO vs Ecad MO PO 7.667 0.419 1.000 Do Not Test

Note: The multiple comparisons on ranks do not include an adjustment for ties.

**Kruskal-Wallis One Way Analysis of Variance on Ranks**

**For Ecad at Normalized Cell Height at 0.9 as shown in Figure 4 E**

**Normality Test (Shapiro-Wilk):**  Failed (P < 0.050)

**Group N Missing Median 25% 75%**

Ctrl MO CO 41 0 1454.562 1125.162 1735.176

Ctrl MO PO 21 0 1447.788 1188.975 1703.739

Ctrl MO NO 40 0 1320.864 1113.317 1607.176

Ecad MO CO 69 0 806.625 540.414 1073.474

Ecad MO PO 20 0 735.661 603.963 976.862

Ecad MO NO 36 0 810.470 620.294 967.767

H = 93.826 with 5 degrees of freedom. (P = <0.001)

The differences in the median values among the treatment groups are greater than would be expected by chance; there is a statistically significant difference (P = <0.001)

To isolate the group or groups that differ from the others use a multiple comparison procedure.

All Pairwise Multiple Comparison Procedures (Dunn's Method) :

**Comparison Diff of Ranks Q P P<0.050**

Ctrl MO PO vs Ecad MO PO 100.164 4.882 <0.001 Yes

Ctrl MO PO vs Ecad MO NO 92.409 5.124 <0.001 Yes

Ctrl MO PO vs Ecad MO CO 92.337 5.642 <0.001 Yes

Ctrl MO PO vs Ctrl MO NO 16.539 0.935 1.000 No

Ctrl MO PO vs Ctrl MO CO 6.934 0.393 1.000 Do Not Test

Ctrl MO CO vs Ecad MO PO 93.230 5.205 <0.001 Yes

Ctrl MO CO vs Ecad MO NO 85.475 5.698 <0.001 Yes

Ctrl MO CO vs Ecad MO CO 85.404 6.595 <0.001 Yes

Ctrl MO CO vs Ctrl MO NO 9.605 0.658 1.000 Do Not Test

Ctrl MO NO vs Ecad MO PO 83.625 4.650 <0.001 Yes

Ctrl MO NO vs Ecad MO NO 75.869 5.029 <0.001 Yes

Ctrl MO NO vs Ecad MO CO 75.798 5.808 <0.001 Yes

Ecad MO CO vs Ecad MO PO 7.827 0.469 1.000 No

Ecad MO CO vs Ecad MO NO 0.0713 0.00528 1.000 Do Not Test

Ecad MO NO vs Ecad MO PO 7.756 0.423 1.000 Do Not Test

Note: The multiple comparisons on ranks do not include an adjustment for ties.

**Kruskal-Wallis One Way Analysis of Variance on Ranks**

**For Ecad at Normalized Cell Height at 0.1 as shown in Figure 4 F**

**Normality Test (Shapiro-Wilk):**  Failed (P < 0.050)

**Group N Missing Median 25% 75%**

Ctrl MO C-C 37 0 1666.623 1369.614 2101.492

Ctrl MO NC-C 78 0 1537.470 1234.013 1906.578

Ctrl MO NC-NC 64 0 1738.008 1298.523 2049.764

Ecad MO C-C 42 0 737.578 566.378 865.776

Ecad MO NC-C 89 0 905.444 671.038 1302.776

Ecad MO NC-NC 48 0 997.537 761.428 1442.843

H = 155.008 with 5 degrees of freedom. (P = <0.001)

The differences in the median values among the treatment groups are greater than would be expected by chance; there is a statistically significant difference (P = <0.001)

To isolate the group or groups that differ from the others use a multiple comparison procedure.

All Pairwise Multiple Comparison Procedures (Dunn's Method) :

**Comparison Diff of Ranks Q P P<0.050**

Ctrl MO C-C vs Ecad MO C-C 189.553 8.124 <0.001 Yes

Ctrl MO C-C vs Ecad MO NC-C 136.480 6.742 <0.001 Yes

Ctrl MO C-C vs Ecad MO NC-NC 114.274 5.047 <0.001 Yes

Ctrl MO C-C vs Ctrl MO NC-C 25.405 1.230 1.000 No

Ctrl MO C-C vs Ctrl MO NC-NC 6.289 0.294 1.000 Do Not Test

Ctrl MO NC-NC vs Ecad MO C-C 183.264 8.917 <0.001 Yes

Ctrl MO NC-NC vs Ecad MO NC-C 130.191 7.676 <0.001 Yes

Ctrl MO NC-NC vs Ecad MO NC-NC 107.984 5.465 <0.001 Yes

Ctrl MO NC-NC vs Ctrl MO NC-C 19.116 1.095 1.000 Do Not Test

Ctrl MO NC-C vs Ecad MO C-C 164.148 8.287 <0.001 Yes

Ctrl MO NC-C vs Ecad MO NC-C 111.075 6.920 <0.001 Yes

Ctrl MO NC-C vs Ecad MO NC-NC 88.869 4.681 <0.001 Yes

Ecad MO NC-NC vs Ecad MO C-C 75.280 3.443 0.009 Yes

Ecad MO NC-NC vs Ecad MO NC-C 22.206 1.198 1.000 No

Ecad MO NC-C vs Ecad MO C-C 53.073 2.739 0.092 No

Note: The multiple comparisons on ranks do not include an adjustment for ties.

**Kruskal-Wallis One Way Analysis of Variance on Ranks**

**For Ecad at Normalized Cell Height at 0.3 as shown in Figure 4 F**

**Normality Test (Shapiro-Wilk):**  Failed (P < 0.050)

**Group N Missing Median 25% 75%**

Ctrl MO C-C 37 0 1552.477 1232.865 1843.276

Ctrl MO NC-C 78 0 1465.116 1121.619 1828.459

Ctrl MO NC-NC 64 0 1478.501 1141.852 1836.639

Ecad MO C-C 42 0 643.458 554.859 794.618

Ecad MO NC-C 89 0 735.757 570.199 1165.436

Ecad MO NC-NC 48 0 857.062 560.642 1174.808

H = 147.691 with 5 degrees of freedom. (P = <0.001)

The differences in the median values among the treatment groups are greater than would be expected by chance; there is a statistically significant difference (P = <0.001)

To isolate the group or groups that differ from the others use a multiple comparison procedure.

All Pairwise Multiple Comparison Procedures (Dunn's Method) :

**Comparison Diff of Ranks Q P P<0.050**

Ctrl MO C-C vs Ecad MO C-C 176.185 7.551 <0.001 Yes

Ctrl MO C-C vs Ecad MO NC-C 132.802 6.560 <0.001 Yes

Ctrl MO C-C vs Ecad MO NC-NC 125.653 5.550 <0.001 Yes

Ctrl MO C-C vs Ctrl MO NC-C 18.462 0.894 1.000 No

Ctrl MO C-C vs Ctrl MO NC-NC 9.522 0.446 1.000 Do Not Test

Ctrl MO NC-NC vs Ecad MO C-C 166.663 8.110 <0.001 Yes

Ctrl MO NC-NC vs Ecad MO NC-C 123.279 7.268 <0.001 Yes

Ctrl MO NC-NC vs Ecad MO NC-NC 116.130 5.877 <0.001 Yes

Ctrl MO NC-NC vs Ctrl MO NC-C 8.940 0.512 1.000 Do Not Test

Ctrl MO NC-C vs Ecad MO C-C 157.723 7.963 <0.001 Yes

Ctrl MO NC-C vs Ecad MO NC-C 114.340 7.123 <0.001 Yes

Ctrl MO NC-C vs Ecad MO NC-NC 107.191 5.646 <0.001 Yes

Ecad MO NC-NC vs Ecad MO C-C 50.533 2.311 0.312 No

Ecad MO NC-NC vs Ecad MO NC-C 7.149 0.386 1.000 Do Not Test

Ecad MO NC-C vs Ecad MO C-C 43.384 2.239 0.377 Do Not Test

Note: The multiple comparisons on ranks do not include an adjustment for ties.

**Kruskal-Wallis One Way Analysis of Variance on Ranks**

**For Ecad at Normalized Cell Height at 0.5 as shown in Figure 4 F**

**Normality Test (Shapiro-Wilk):**  Failed (P < 0.050)

**Group N Missing Median 25% 75%**

Ctrl MO C-C 37 0 1136.387 972.085 1464.884

Ctrl MO NC-C 78 0 1115.320 831.338 1437.646

Ctrl MO NC-NC 64 0 1043.050 774.036 1435.663

Ecad MO C-C 42 0 517.940 403.115 628.925

Ecad MO NC-C 89 0 580.738 405.221 879.544

Ecad MO NC-NC 48 0 674.350 434.886 823.375

H = 129.649 with 5 degrees of freedom. (P = <0.001)

The differences in the median values among the treatment groups are greater than would be expected by chance; there is a statistically significant difference (P = <0.001)

To isolate the group or groups that differ from the others use a multiple comparison procedure.

All Pairwise Multiple Comparison Procedures (Dunn's Method) :

**Comparison Diff of Ranks Q P P<0.050**

Ctrl MO C-C vs Ecad MO C-C 163.506 7.007 <0.001 Yes

Ctrl MO C-C vs Ecad MO NC-NC 124.617 5.504 <0.001 Yes

Ctrl MO C-C vs Ecad MO NC-C 124.200 6.135 <0.001 Yes

Ctrl MO C-C vs Ctrl MO NC-NC 18.460 0.864 1.000 No

Ctrl MO C-C vs Ctrl MO NC-C 11.413 0.552 1.000 Do Not Test

Ctrl MO NC-C vs Ecad MO C-C 152.093 7.679 <0.001 Yes

Ctrl MO NC-C vs Ecad MO NC-NC 113.204 5.963 <0.001 Yes

Ctrl MO NC-C vs Ecad MO NC-C 112.787 7.027 <0.001 Yes

Ctrl MO NC-C vs Ctrl MO NC-NC 7.047 0.404 1.000 Do Not Test

Ctrl MO NC-NC vs Ecad MO C-C 145.046 7.058 <0.001 Yes

Ctrl MO NC-NC vs Ecad MO NC-NC 106.156 5.372 <0.001 Yes

Ctrl MO NC-NC vs Ecad MO NC-C 105.740 6.234 <0.001 Yes

Ecad MO NC-C vs Ecad MO C-C 39.306 2.029 0.637 No

Ecad MO NC-C vs Ecad MO NC-NC 0.416 0.0225 1.000 Do Not Test

Ecad MO NC-NC vs Ecad MO C-C 38.890 1.779 1.000 Do Not Test

Note: The multiple comparisons on ranks do not include an adjustment for ties.

**Kruskal-Wallis One Way Analysis of Variance on Ranks**

**For Ecad at Normalized Cell Height at 0.7 as shown in Figure 4 F**

**Normality Test (Shapiro-Wilk):**  Failed (P < 0.050)

**Group N Missing Median 25% 75%**

Ctrl MO C-C 37 0 852.123 613.461 1117.996

Ctrl MO NC-C 78 0 814.611 589.499 1136.848

Ctrl MO NC-NC 64 0 738.918 490.511 1013.779

Ecad MO C-C 42 0 360.806 297.555 471.410

Ecad MO NC-C 89 0 454.332 322.082 708.334

Ecad MO NC-NC 48 0 432.221 281.461 619.607

H = 112.530 with 5 degrees of freedom. (P = <0.001)

The differences in the median values among the treatment groups are greater than would be expected by chance; there is a statistically significant difference (P = <0.001)

To isolate the group or groups that differ from the others use a multiple comparison procedure.

All Pairwise Multiple Comparison Procedures (Dunn's Method) :

**Comparison Diff of Ranks Q P P<0.050**

Ctrl MO C-C vs Ecad MO C-C 155.652 6.671 <0.001 Yes

Ctrl MO C-C vs Ecad MO NC-NC 124.217 5.487 <0.001 Yes

Ctrl MO C-C vs Ecad MO NC-C 111.676 5.517 <0.001 Yes

Ctrl MO C-C vs Ctrl MO NC-NC 26.566 1.243 1.000 No

Ctrl MO C-C vs Ctrl MO NC-C 8.022 0.388 1.000 Do Not Test

Ctrl MO NC-C vs Ecad MO C-C 147.630 7.453 <0.001 Yes

Ctrl MO NC-C vs Ecad MO NC-NC 116.196 6.120 <0.001 Yes

Ctrl MO NC-C vs Ecad MO NC-C 103.654 6.458 <0.001 Yes

Ctrl MO NC-C vs Ctrl MO NC-NC 18.544 1.062 1.000 Do Not Test

Ctrl MO NC-NC vs Ecad MO C-C 129.086 6.281 <0.001 Yes

Ctrl MO NC-NC vs Ecad MO NC-NC 97.651 4.942 <0.001 Yes

Ctrl MO NC-NC vs Ecad MO NC-C 85.109 5.018 <0.001 Yes

Ecad MO NC-C vs Ecad MO C-C 43.976 2.270 0.348 No

Ecad MO NC-C vs Ecad MO NC-NC 12.542 0.677 1.000 Do Not Test

Ecad MO NC-NC vs Ecad MO C-C 31.435 1.438 1.000 Do Not Test

Note: The multiple comparisons on ranks do not include an adjustment for ties.

**Kruskal-Wallis One Way Analysis of Variance on Ranks**

**For Ecad at Normalized Cell Height at 0.9 as shown in Figure 4 F**

**Normality Test (Shapiro-Wilk):**  Failed (P < 0.050)

**Group N Missing Median 25% 75%**

Ctrl MO C-C 37 0 598.709 451.491 835.963

Ctrl MO NC-C 78 0 567.813 419.840 844.135

Ctrl MO NC-NC 64 0 484.315 356.585 699.088

Ecad MO C-C 42 0 285.683 230.144 444.441

Ecad MO NC-C 89 0 335.718 225.291 543.981

Ecad MO NC-NC 48 0 298.059 185.503 514.222

H = 80.872 with 5 degrees of freedom. (P = <0.001)

The differences in the median values among the treatment groups are greater than would be expected by chance; there is a statistically significant difference (P = <0.001)

To isolate the group or groups that differ from the others use a multiple comparison procedure.

All Pairwise Multiple Comparison Procedures (Dunn's Method) :

**Comparison Diff of Ranks Q P P<0.050**

Ctrl MO C-C vs Ecad MO C-C 133.644 5.727 <0.001 Yes

Ctrl MO C-C vs Ecad MO NC-NC 123.718 5.464 <0.001 Yes

Ctrl MO C-C vs Ecad MO NC-C 105.147 5.194 <0.001 Yes

Ctrl MO C-C vs Ctrl MO NC-NC 44.343 2.075 0.570 No

Ctrl MO C-C vs Ctrl MO NC-C 16.393 0.794 1.000 Do Not Test

Ctrl MO NC-C vs Ecad MO C-C 117.251 5.920 <0.001 Yes

Ctrl MO NC-C vs Ecad MO NC-NC 107.325 5.653 <0.001 Yes

Ctrl MO NC-C vs Ecad MO NC-C 88.754 5.529 <0.001 Yes

Ctrl MO NC-C vs Ctrl MO NC-NC 27.950 1.601 1.000 Do Not Test

Ctrl MO NC-NC vs Ecad MO C-C 89.301 4.345 <0.001 Yes

Ctrl MO NC-NC vs Ecad MO NC-NC 79.375 4.017 <0.001 Yes

Ctrl MO NC-NC vs Ecad MO NC-C 60.804 3.585 0.005 Yes

Ecad MO NC-C vs Ecad MO C-C 28.497 1.471 1.000 No

Ecad MO NC-C vs Ecad MO NC-NC 18.571 1.002 1.000 Do Not Test

Ecad MO NC-NC vs Ecad MO C-C 9.926 0.454 1.000 Do Not Test

Note: The multiple comparisons on ranks do not include an adjustment for ties.
