# Supplementary material for: Stepwise polarisation of developing bilayered epidermis is mediated by aPKC and E-cadherin in zebrafish
Source: eLife. 2020 Jan 22;9:e49064. doi: 10.7554/eLife.49064 (PMC6975926; doi:10.7554/eLife.49064)
Supplement: Figure 4—figure supplement 2—source data 9. [file elife-49064-fig4-figsupp2-data9.docx]

Statistical comparisons between Ctrl MO and Ecad MO at boundary Figure 4 figure supplement 2

**Color code:**

Comparisons showing high statisitcal significance

Comparisons showing weak statistical significance (p < 0.08), possibly arising due to large variations, may be biologically relevant

**Kruskal-Wallis One Way Analysis of Variance on Ranks**

**For Ecad at Normalized Cell Height at 0.1 as shown in Figure 4- figure supplement 2 A1**

**Normality Test (Shapiro-Wilk):**  Passed (P = 0.423)

**Equal Variance Test (Brown-Forsythe):** Failed (P < 0.050)

**Group N Missing Median 25% 75%**

Ctrl MO C-C 13 0 246.253 200.963 292.379

Ctrl MO N-C 37 0 241.669 198.905 298.961

Ctrl MO N-N 26 0 259.023 226.569 317.898

Ecad MO C-C 31 0 189.446 149.083 225.329

Ecad MO N-C 50 0 210.299 177.684 244.503

Ecad MO N-N 26 0 291.993 245.442 325.565

H = 52.122 with 5 degrees of freedom. (P = <0.001)

The differences in the median values among the treatment groups are greater than would be expected by chance; there is a statistically significant difference (P = <0.001)

To isolate the group or groups that differ from the others use a multiple comparison procedure.

All Pairwise Multiple Comparison Procedures (Dunn's Method) :

**Comparison Diff of Ranks Q P P<0.050**

Ecad MO N-N vs Ecad MO C-C 82.468 5.854 <0.001 Yes

Ecad MO N-N vs Ecad MO N-C 61.420 4.795 <0.001 Yes

Ecad MO N-N vs Ctrl MO N-C 30.365 2.240 0.376 No

Ecad MO N-N vs Ctrl MO C-C 26.192 1.456 1.000 Do Not Test

Ecad MO N-N vs Ctrl MO N-N 12.308 0.838 1.000 Do Not Test

Ctrl MO N-N vs Ecad MO C-C 70.160 4.981 <0.001 Yes

Ctrl MO N-N vs Ecad MO N-C 49.112 3.835 0.002 Yes

Ctrl MO N-N vs Ctrl MO N-C 18.057 1.332 1.000 Do Not Test

Ctrl MO N-N vs Ctrl MO C-C 13.885 0.772 1.000 Do Not Test

Ctrl MO C-C vs Ecad MO C-C 56.275 3.215 0.020 Yes

Ctrl MO C-C vs Ecad MO N-C 35.228 2.136 0.490 No

Ctrl MO C-C vs Ctrl MO N-C 4.173 0.244 1.000 Do Not Test

Ctrl MO N-C vs Ecad MO C-C 52.103 4.040 <0.001 Yes

Ctrl MO N-C vs Ecad MO N-C 31.055 2.703 0.103 Do Not Test

Ecad MO N-C vs Ecad MO C-C 21.048 1.738 1.000 No

**Kruskal-Wallis One Way Analysis of Variance on Ranks**

**For Ecad at Normalized Cell Height at 0.3 as shown in Figure 4- figure supplement 2 A1**

**Normality Test (Shapiro-Wilk):**  Passed (P = 0.679)

**Equal Variance Test (Brown-Forsythe):** Failed (P < 0.050)

**Group N Missing Median 25% 75%**

Ctrl MO C-C 13 0 310.533 248.669 350.646

Ctrl MO N-C 37 0 295.376 252.671 331.408

Ctrl MO N-N 26 0 305.795 275.451 371.189

Ecad MO C-C 31 0 220.193 171.519 260.266

Ecad MO N-C 50 0 249.642 208.395 282.790

Ecad MO N-N 26 0 318.609 266.463 421.816

H = 54.980 with 5 degrees of freedom. (P = <0.001)

The differences in the median values among the treatment groups are greater than would be expected by chance; there is a statistically significant difference (P = <0.001)

To isolate the group or groups that differ from the others use a multiple comparison procedure.

All Pairwise Multiple Comparison Procedures (Dunn's Method) :

**Comparison Diff of Ranks Q P P<0.050**

Ecad MO N-N vs Ecad MO C-C 77.233 5.483 <0.001 Yes

Ecad MO N-N vs Ecad MO N-C 53.526 4.179 <0.001 Yes

Ecad MO N-N vs Ctrl MO N-C 16.873 1.245 1.000 No

Ecad MO N-N vs Ctrl MO C-C 9.462 0.526 1.000 Do Not Test

Ecad MO N-N vs Ctrl MO N-N 0.385 0.0262 1.000 Do Not Test

Ctrl MO N-N vs Ecad MO C-C 76.849 5.455 <0.001 Yes

Ctrl MO N-N vs Ecad MO N-C 53.142 4.149 <0.001 Yes

Ctrl MO N-N vs Ctrl MO N-C 16.489 1.216 1.000 Do Not Test

Ctrl MO N-N vs Ctrl MO C-C 9.077 0.504 1.000 Do Not Test

Ctrl MO C-C vs Ecad MO C-C 67.772 3.872 0.002 Yes

Ctrl MO C-C vs Ecad MO N-C 44.065 2.672 0.113 No

Ctrl MO C-C vs Ctrl MO N-C 7.412 0.434 1.000 Do Not Test

Ctrl MO N-C vs Ecad MO C-C 60.360 4.680 <0.001 Yes

Ctrl MO N-C vs Ecad MO N-C 36.653 3.191 0.021 Do Not Test

Ecad MO N-C vs Ecad MO C-C 23.707 1.958 0.754 No

**Kruskal-Wallis One Way Analysis of Variance on Ranks**

**For Ecad at Normalized Cell Height at 0.5 as shown in Figure 4- figure supplement 2 A1**

**Normality Test (Shapiro-Wilk):**  Failed (P < 0.050)

**Group N Missing Median 25% 75%**

Ctrl MO C-C 13 0 368.094 286.484 400.855

Ctrl MO N-C 37 0 326.952 286.113 377.160

Ctrl MO N-N 26 0 362.306 300.268 412.507

Ecad MO C-C 31 0 235.478 172.517 296.291

Ecad MO N-C 50 0 261.001 223.462 333.898

Ecad MO N-N 26 0 326.681 265.957 552.886

H = 47.444 with 5 degrees of freedom. (P = <0.001)

The differences in the median values among the treatment groups are greater than would be expected by chance; there is a statistically significant difference (P = <0.001)

To isolate the group or groups that differ from the others use a multiple comparison procedure.

All Pairwise Multiple Comparison Procedures (Dunn's Method) :

**Comparison Diff of Ranks Q P P<0.050**

Ctrl MO N-N vs Ecad MO C-C 76.164 5.407 <0.001 Yes

Ctrl MO N-N vs Ecad MO N-C 53.415 4.170 <0.001 Yes

Ctrl MO N-N vs Ctrl MO N-C 18.183 1.341 1.000 No

Ctrl MO N-N vs Ecad MO N-N 12.423 0.846 1.000 Do Not Test

Ctrl MO N-N vs Ctrl MO C-C 9.538 0.530 1.000 Do Not Test

Ctrl MO C-C vs Ecad MO C-C 66.625 3.806 0.002 Yes

Ctrl MO C-C vs Ecad MO N-C 43.877 2.661 0.117 No

Ctrl MO C-C vs Ctrl MO N-C 8.644 0.506 1.000 Do Not Test

Ctrl MO C-C vs Ecad MO N-N 2.885 0.160 1.000 Do Not Test

Ecad MO N-N vs Ecad MO C-C 63.741 4.525 <0.001 Yes

Ecad MO N-N vs Ecad MO N-C 40.992 3.201 0.021 Do Not Test

Ecad MO N-N vs Ctrl MO N-C 5.760 0.425 1.000 Do Not Test

Ctrl MO N-C vs Ecad MO C-C 57.981 4.495 <0.001 Yes

Ctrl MO N-C vs Ecad MO N-C 35.232 3.067 0.032 Do Not Test

Ecad MO N-C vs Ecad MO C-C 22.748 1.879 0.905 No

**Kruskal-Wallis One Way Analysis of Variance on Ranks**

**For Ecad at Normalized Cell Height at 0.7 as shown in Figure 4- figure supplement 2 A1**

**Normality Test (Shapiro-Wilk):**  Failed (P < 0.050)

**Group N Missing Median 25% 75%**

Ctrl MO C-C 13 0 389.805 286.362 457.190

Ctrl MO N-C 37 0 344.210 291.752 416.139

Ctrl MO N-N 26 0 380.024 318.203 466.284

Ecad MO C-C 31 0 241.934 193.110 321.852

Ecad MO N-C 50 0 286.881 238.694 386.285

Ecad MO N-N 26 0 375.258 303.601 512.961

H = 38.898 with 5 degrees of freedom. (P = <0.001)

The differences in the median values among the treatment groups are greater than would be expected by chance; there is a statistically significant difference (P = <0.001)

To isolate the group or groups that differ from the others use a multiple comparison procedure.

All Pairwise Multiple Comparison Procedures (Dunn's Method) :

**Comparison Diff of Ranks Q P P<0.050**

Ctrl MO N-N vs Ecad MO C-C 69.249 4.916 <0.001 Yes

Ctrl MO N-N vs Ecad MO N-C 46.886 3.661 0.004 Yes

Ctrl MO N-N vs Ctrl MO N-C 21.643 1.597 1.000 No

Ctrl MO N-N vs Ecad MO N-N 7.077 0.482 1.000 Do Not Test

Ctrl MO N-N vs Ctrl MO C-C 5.962 0.331 1.000 Do Not Test

Ctrl MO C-C vs Ecad MO C-C 63.288 3.616 0.004 Yes

Ctrl MO C-C vs Ecad MO N-C 40.925 2.482 0.196 No

Ctrl MO C-C vs Ctrl MO N-C 15.682 0.918 1.000 Do Not Test

Ctrl MO C-C vs Ecad MO N-N 1.115 0.0620 1.000 Do Not Test

Ecad MO N-N vs Ecad MO C-C 62.172 4.414 <0.001 Yes

Ecad MO N-N vs Ecad MO N-C 39.809 3.108 0.028 Do Not Test

Ecad MO N-N vs Ctrl MO N-C 14.567 1.075 1.000 Do Not Test

Ctrl MO N-C vs Ecad MO C-C 47.606 3.691 0.003 Yes

Ctrl MO N-C vs Ecad MO N-C 25.243 2.197 0.420 Do Not Test

Ecad MO N-C vs Ecad MO C-C 22.363 1.847 0.972 No

Note: The multiple comparisons on ranks do not include an adjustment for ties.

**Kruskal-Wallis One Way Analysis of Variance on Ranks**

**For Ecad at Normalized Cell Height at 0.9 as shown in Figure 4- figure supplement 2 A1**

**Normality Test (Shapiro-Wilk):**  Failed (P < 0.050)

**Group N Missing Median 25% 75%**

Ctrl MO C-C 13 0 407.884 286.861 483.332

Ctrl MO N-C 37 0 363.798 296.838 423.813

Ctrl MO N-N 26 0 360.989 313.161 488.225

Ecad MO C-C 31 0 261.606 196.743 357.783

Ecad MO N-C 50 0 311.166 261.618 409.011

Ecad MO N-N 26 0 406.135 318.919 506.178

H = 25.142 with 5 degrees of freedom. (P = <0.001)

The differences in the median values among the treatment groups are greater than would be expected by chance; there is a statistically significant difference (P = <0.001)

To isolate the group or groups that differ from the others use a multiple comparison procedure.

All Pairwise Multiple Comparison Procedures (Dunn's Method) :

**Comparison Diff of Ranks Q P P<0.050**

Ecad MO N-N vs Ecad MO C-C 56.447 4.007 <0.001 Yes

Ecad MO N-N vs Ecad MO N-C 31.329 2.446 0.217 No

Ecad MO N-N vs Ctrl MO N-C 15.607 1.151 1.000 Do Not Test

Ecad MO N-N vs Ctrl MO C-C 5.538 0.308 1.000 Do Not Test

Ecad MO N-N vs Ctrl MO N-N 0.692 0.0471 1.000 Do Not Test

Ctrl MO N-N vs Ecad MO C-C 55.754 3.958 0.001 Yes

Ctrl MO N-N vs Ecad MO N-C 30.637 2.392 0.251 Do Not Test

Ctrl MO N-N vs Ctrl MO N-C 14.915 1.100 1.000 Do Not Test

Ctrl MO N-N vs Ctrl MO C-C 4.846 0.269 1.000 Do Not Test

Ctrl MO C-C vs Ecad MO C-C 50.908 2.909 0.054 No

Ctrl MO C-C vs Ecad MO N-C 25.791 1.564 1.000 Do Not Test

Ctrl MO C-C vs Ctrl MO N-C 10.069 0.590 1.000 Do Not Test

Ctrl MO N-C vs Ecad MO C-C 40.840 3.166 0.023 Do Not Test

Ctrl MO N-C vs Ecad MO N-C 15.722 1.369 1.000 Do Not Test

Ecad MO N-C vs Ecad MO C-C 25.117 2.074 0.571 Do Not Test

Note: The multiple comparisons on ranks do not include an adjustment for ties.

**Kruskal-Wallis One Way Analysis of Variance on Ranks**

**For Ecad at Normalized Cell Height at 0.1 as shown in Figure 4- figure supplement 2 A2**

**Normality Test (Shapiro-Wilk):**  Passed (P = 0.062)

**Equal Variance Test (Brown-Forsythe):** Failed (P < 0.050)

**Group N Missing Median 25% 75%**

Ctrl MO CO 30 0 497.553 422.836 609.899

Ctrl MO PO 16 0 508.412 403.993 583.313

Ctrl MO NO 23 0 508.309 433.113 566.771

Ecad MO CO 41 0 445.251 273.037 529.356

Ecad MO PO 22 0 379.780 263.126 660.500

Ecad MO NO 27 0 554.549 328.428 718.347

H = 8.871 with 5 degrees of freedom. (P = 0.114)

The differences in the median values among the treatment groups are not great enough to exclude the possibility that the difference is due to random sampling variability; there is not a statistically significant difference (P = 0.114)

All Pairwise Multiple Comparison Procedures (Dunn's Method) :

**Comparison Diff of Ranks Q P P<0.050**

Ctrl MO NO vs Ecad MO CO 24.273 2.024 0.645 No

Ctrl MO NO vs Ecad MO PO 23.020 1.676 1.000 Do Not Test

Ctrl MO NO vs Ctrl MO PO 5.565 0.371 1.000 Do Not Test

Ctrl MO NO vs Ctrl MO CO 2.865 0.225 1.000 Do Not Test

Ctrl MO NO vs Ecad MO NO 0.121 0.00924 1.000 Do Not Test

Ecad MO NO vs Ecad MO CO 24.152 2.116 0.515 Do Not Test

Ecad MO NO vs Ecad MO PO 22.899 1.732 1.000 Do Not Test

Ecad MO NO vs Ctrl MO PO 5.444 0.375 1.000 Do Not Test

Ecad MO NO vs Ctrl MO CO 2.744 0.225 1.000 Do Not Test

Ctrl MO CO vs Ecad MO CO 21.407 1.935 0.795 Do Not Test

Ctrl MO CO vs Ecad MO PO 20.155 1.559 1.000 Do Not Test

Ctrl MO CO vs Ctrl MO PO 2.700 0.189 1.000 Do Not Test

Ctrl MO PO vs Ecad MO CO 18.707 1.378 1.000 Do Not Test

Ctrl MO PO vs Ecad MO PO 17.455 1.154 1.000 Do Not Test

Ecad MO PO vs Ecad MO CO 1.253 0.103 1.000 Do Not Test

Note: The multiple comparisons on ranks do not include an adjustment for ties.

**Kruskal-Wallis One Way Analysis of Variance on Ranks**

**For Ecad at Normalized Cell Height at 0.3 as shown in Figure 4- figure supplement 2 A2**

**Normality Test (Shapiro-Wilk):**  Passed (P = 0.108)

**Equal Variance Test (Brown-Forsythe):** Failed (P < 0.050)

**Group N Missing Median 25% 75%**

Ctrl MO CO 30 0 466.873 414.460 547.258

Ctrl MO PO 16 0 490.596 377.771 555.943

Ctrl MO NO 23 0 447.680 362.018 548.241

Ecad MO CO 41 0 365.700 266.929 435.611

Ecad MO PO 22 0 342.115 253.294 499.451

Ecad MO NO 27 0 454.680 300.582 684.182

H = 20.942 with 5 degrees of freedom. (P = <0.001)

The differences in the median values among the treatment groups are greater than would be expected by chance; there is a statistically significant difference (P = <0.001)

To isolate the group or groups that differ from the others use a multiple comparison procedure.

All Pairwise Multiple Comparison Procedures (Dunn's Method) :

**Comparison Diff of Ranks Q P P<0.050**

Ctrl MO PO vs Ecad MO CO 38.180 2.813 0.074 No

Ctrl MO PO vs Ecad MO PO 31.193 2.062 0.588 Do Not Test

Ctrl MO PO vs Ctrl MO NO 5.592 0.373 1.000 Do Not Test

Ctrl MO PO vs Ecad MO NO 1.745 0.120 1.000 Do Not Test

Ctrl MO PO vs Ctrl MO CO 0.575 0.0403 1.000 Do Not Test

Ctrl MO CO vs Ecad MO CO 37.605 3.399 0.010 Do Not Test

Ctrl MO CO vs Ecad MO PO 30.618 2.369 0.267 Do Not Test

Ctrl MO CO vs Ctrl MO NO 5.017 0.393 1.000 Do Not Test

Ctrl MO CO vs Ecad MO NO 1.170 0.0958 1.000 Do Not Test

Ecad MO NO vs Ecad MO CO 36.435 3.193 0.021 Do Not Test

Ecad MO NO vs Ecad MO PO 29.448 2.227 0.389 Do Not Test

Ecad MO NO vs Ctrl MO NO 3.847 0.294 1.000 Do Not Test

Ctrl MO NO vs Ecad MO CO 32.587 2.717 0.099 Do Not Test

Ctrl MO NO vs Ecad MO PO 25.601 1.864 0.934 Do Not Test

Ecad MO PO vs Ecad MO CO 6.987 0.574 1.000 Do Not Test

Note: The multiple comparisons on ranks do not include an adjustment for ties.

**Kruskal-Wallis One Way Analysis of Variance on Ranks**

**For Ecad at Normalized Cell Height at 0.5 as shown in Figure 4- figure supplement 2 A2**

**Normality Test (Shapiro-Wilk):**  Passed (P = 0.324)

**Equal Variance Test (Brown-Forsythe):** Failed (P < 0.050)

**Group N Missing Median 25% 75%**

Ctrl MO CO 30 0 390.423 343.382 438.388

Ctrl MO PO 16 0 426.818 330.625 500.016

Ctrl MO NO 23 0 357.480 287.064 447.916

Ecad MO CO 41 0 282.909 228.267 337.536

Ecad MO PO 22 0 299.298 223.060 392.640

Ecad MO NO 27 0 398.884 259.675 510.655

H = 30.655 with 5 degrees of freedom. (P = <0.001)

The differences in the median values among the treatment groups are greater than would be expected by chance; there is a statistically significant difference (P = <0.001)

To isolate the group or groups that differ from the others use a multiple comparison procedure.

All Pairwise Multiple Comparison Procedures (Dunn's Method) :

**Comparison Diff of Ranks Q P P<0.050**

Ctrl MO PO vs Ecad MO CO 51.521 3.796 0.002 Yes

Ctrl MO PO vs Ecad MO PO 40.102 2.651 0.120 No

Ctrl MO PO vs Ctrl MO NO 14.245 0.950 1.000 Do Not Test

Ctrl MO PO vs Ecad MO NO 9.153 0.630 1.000 Do Not Test

Ctrl MO PO vs Ctrl MO CO 4.908 0.344 1.000 Do Not Test

Ctrl MO CO vs Ecad MO CO 46.613 4.214 <0.001 Yes

Ctrl MO CO vs Ecad MO PO 35.194 2.723 0.097 Do Not Test

Ctrl MO CO vs Ctrl MO NO 9.336 0.732 1.000 Do Not Test

Ctrl MO CO vs Ecad MO NO 4.244 0.348 1.000 Do Not Test

Ecad MO NO vs Ecad MO CO 42.369 3.713 0.003 Yes

Ecad MO NO vs Ecad MO PO 30.949 2.340 0.289 Do Not Test

Ecad MO NO vs Ctrl MO NO 5.092 0.390 1.000 Do Not Test

Ctrl MO NO vs Ecad MO CO 37.277 3.108 0.028 Yes

Ctrl MO NO vs Ecad MO PO 25.858 1.883 0.895 Do Not Test

Ecad MO PO vs Ecad MO CO 11.419 0.938 1.000 No

Note: The multiple comparisons on ranks do not include an adjustment for ties.

**Kruskal-Wallis One Way Analysis of Variance on Ranks**

**For Ecad at Normalized Cell Height at 0.7 as shown in Figure 4- figure supplement 2 A2**

**Normality Test (Shapiro-Wilk):**  Failed (P < 0.050)

**Group N Missing Median 25% 75%**

Ctrl MO CO 30 0 320.686 284.714 358.544

Ctrl MO PO 16 0 342.255 260.922 402.027

Ctrl MO NO 23 0 278.888 241.638 350.287

Ecad MO CO 41 0 228.981 196.427 280.098

Ecad MO PO 22 0 257.683 187.140 321.278

Ecad MO NO 27 0 333.821 226.184 420.533

H = 27.749 with 5 degrees of freedom. (P = <0.001)

The differences in the median values among the treatment groups are greater than would be expected by chance; there is a statistically significant difference (P = <0.001)

To isolate the group or groups that differ from the others use a multiple comparison procedure.

All Pairwise Multiple Comparison Procedures (Dunn's Method) :

**Comparison Diff of Ranks Q P P<0.050**

Ctrl MO PO vs Ecad MO CO 49.498 3.647 0.004 Yes

Ctrl MO PO vs Ecad MO PO 38.574 2.550 0.162 No

Ctrl MO PO vs Ctrl MO NO 17.024 1.136 1.000 Do Not Test

Ctrl MO PO vs Ecad MO NO 8.197 0.564 1.000 Do Not Test

Ctrl MO PO vs Ctrl MO CO 5.204 0.365 1.000 Do Not Test

Ctrl MO CO vs Ecad MO CO 44.294 4.004 <0.001 Yes

Ctrl MO CO vs Ecad MO PO 33.370 2.582 0.147 Do Not Test

Ctrl MO CO vs Ctrl MO NO 11.820 0.926 1.000 Do Not Test

Ctrl MO CO vs Ecad MO NO 2.993 0.245 1.000 Do Not Test

Ecad MO NO vs Ecad MO CO 41.302 3.619 0.004 Yes

Ecad MO NO vs Ecad MO PO 30.377 2.297 0.324 Do Not Test

Ecad MO NO vs Ctrl MO NO 8.828 0.676 1.000 Do Not Test

Ctrl MO NO vs Ecad MO CO 32.474 2.707 0.102 No

Ctrl MO NO vs Ecad MO PO 21.549 1.569 1.000 Do Not Test

Ecad MO PO vs Ecad MO CO 10.925 0.898 1.000 Do Not Test

Note: The multiple comparisons on ranks do not include an adjustment for ties.

**Kruskal-Wallis One Way Analysis of Variance on Ranks**

**For Ecad at Normalized Cell Height at 0.9 as shown in Figure 4- figure supplement 2 A2**

**Normality Test (Shapiro-Wilk):**  Failed (P < 0.050)

**Group N Missing Median 25% 75%**

Ctrl MO CO 30 0 246.128 224.183 282.604

Ctrl MO PO 16 0 270.588 231.109 332.613

Ctrl MO NO 23 0 218.249 193.498 266.918

Ecad MO CO 41 0 191.731 165.516 229.814

Ecad MO PO 22 0 202.967 165.761 255.905

Ecad MO NO 27 0 273.891 183.936 317.952

H = 29.532 with 5 degrees of freedom. (P = <0.001)

The differences in the median values among the treatment groups are greater than would be expected by chance; there is a statistically significant difference (P = <0.001)

To isolate the group or groups that differ from the others use a multiple comparison procedure.

All Pairwise Multiple Comparison Procedures (Dunn's Method) :

**Comparison Diff of Ranks Q P P<0.050**

Ctrl MO PO vs Ecad MO CO 50.977 3.756 0.003 Yes

Ctrl MO PO vs Ecad MO PO 40.290 2.663 0.116 No

Ctrl MO PO vs Ctrl MO NO 24.389 1.627 1.000 Do Not Test

Ctrl MO PO vs Ecad MO NO 8.525 0.587 1.000 Do Not Test

Ctrl MO PO vs Ctrl MO CO 4.596 0.322 1.000 Do Not Test

Ctrl MO CO vs Ecad MO CO 46.381 4.193 <0.001 Yes

Ctrl MO CO vs Ecad MO PO 35.694 2.762 0.086 Do Not Test

Ctrl MO CO vs Ctrl MO NO 19.793 1.551 1.000 Do Not Test

Ctrl MO CO vs Ecad MO NO 3.930 0.322 1.000 Do Not Test

Ecad MO NO vs Ecad MO CO 42.452 3.720 0.003 Yes

Ecad MO NO vs Ecad MO PO 31.764 2.402 0.245 Do Not Test

Ecad MO NO vs Ctrl MO NO 15.863 1.214 1.000 Do Not Test

Ctrl MO NO vs Ecad MO CO 26.589 2.217 0.400 No

Ctrl MO NO vs Ecad MO PO 15.901 1.158 1.000 Do Not Test

Ecad MO PO vs Ecad MO CO 10.687 0.878 1.000 Do Not Test

Note: The multiple comparisons on ranks do not include an adjustment for ties.

**Kruskal-Wallis One Way Analysis of Variance on Ranks**

**For Ecad at Normalized Cell Height at 0.1 as shown in Figure 4- figure supplement 2 B1**

**Normality Test (Shapiro-Wilk):**  Failed (P < 0.050)

**Group N Missing Median 25% 75%**

Ctrl MO CO 25 0 276.874 235.695 326.357

Ctrl MO PO 24 0 280.449 259.113 326.597

Ctrl MO NO 46 0 318.828 263.200 365.669

Ecad MO CO 29 0 216.017 182.859 262.572

Ecad MO PO 13 0 280.429 255.726 353.532

Ecad MO NO 44 0 303.995 240.810 364.192

H = 30.652 with 5 degrees of freedom. (P = <0.001)

The differences in the median values among the treatment groups are greater than would be expected by chance; there is a statistically significant difference (P = <0.001)

To isolate the group or groups that differ from the others use a multiple comparison procedure.

All Pairwise Multiple Comparison Procedures (Dunn's Method) :

**Comparison Diff of Ranks Q P P<0.050**

Ctrl MO NO vs Ecad MO CO 65.748 5.292 <0.001 Yes

Ctrl MO NO vs Ctrl MO CO 18.703 1.437 1.000 No

Ctrl MO NO vs Ctrl MO PO 16.991 1.288 1.000 Do Not Test

Ctrl MO NO vs Ecad MO PO 14.860 0.903 1.000 Do Not Test

Ctrl MO NO vs Ecad MO NO 9.646 0.873 1.000 Do Not Test

Ecad MO NO vs Ecad MO CO 56.102 4.477 <0.001 Yes

Ecad MO NO vs Ctrl MO CO 9.056 0.690 1.000 Do Not Test

Ecad MO NO vs Ctrl MO PO 7.345 0.552 1.000 Do Not Test

Ecad MO NO vs Ecad MO PO 5.213 0.315 1.000 Do Not Test

Ecad MO PO vs Ecad MO CO 50.889 2.910 0.054 No

Ecad MO PO vs Ctrl MO CO 3.843 0.215 1.000 Do Not Test

Ecad MO PO vs Ctrl MO PO 2.131 0.118 1.000 Do Not Test

Ctrl MO PO vs Ecad MO CO 48.757 3.372 0.011 Do Not Test

Ctrl MO PO vs Ctrl MO CO 1.712 0.114 1.000 Do Not Test

Ctrl MO CO vs Ecad MO CO 47.046 3.290 0.015 Do Not Test

Note: The multiple comparisons on ranks do not include an adjustment for ties.

**Kruskal-Wallis One Way Analysis of Variance on Ranks**

**For Ecad at Normalized Cell Height at 0.3 as shown in Figure 4- figure supplement 2 B1**

**Normality Test (Shapiro-Wilk):**  Failed (P < 0.050)

**Group N Missing Median 25% 75%**

Ctrl MO CO 25 0 316.701 270.440 379.092

Ctrl MO PO 24 0 309.229 286.744 347.899

Ctrl MO NO 46 0 357.084 303.298 410.157

Ecad MO CO 29 0 233.913 200.457 321.134

Ecad MO PO 13 0 328.027 283.848 402.568

Ecad MO NO 44 0 329.735 263.301 408.370

H = 25.735 with 5 degrees of freedom. (P = <0.001)

The differences in the median values among the treatment groups are greater than would be expected by chance; there is a statistically significant difference (P = <0.001)

To isolate the group or groups that differ from the others use a multiple comparison procedure.

All Pairwise Multiple Comparison Procedures (Dunn's Method) :

**Comparison Diff of Ranks Q P P<0.050**

Ctrl MO NO vs Ecad MO CO 60.999 4.910 <0.001 Yes

Ctrl MO NO vs Ctrl MO PO 23.330 1.768 1.000 No

Ctrl MO NO vs Ctrl MO CO 19.053 1.464 1.000 Do Not Test

Ctrl MO NO vs Ecad MO PO 13.105 0.796 1.000 Do Not Test

Ctrl MO NO vs Ecad MO NO 12.231 1.107 1.000 Do Not Test

Ecad MO NO vs Ecad MO CO 48.768 3.891 0.001 Yes

Ecad MO NO vs Ctrl MO PO 11.098 0.835 1.000 Do Not Test

Ecad MO NO vs Ctrl MO CO 6.822 0.520 1.000 Do Not Test

Ecad MO NO vs Ecad MO PO 0.874 0.0529 1.000 Do Not Test

Ecad MO PO vs Ecad MO CO 47.894 2.739 0.093 No

Ecad MO PO vs Ctrl MO PO 10.224 0.567 1.000 Do Not Test

Ecad MO PO vs Ctrl MO CO 5.948 0.332 1.000 Do Not Test

Ctrl MO CO vs Ecad MO CO 41.946 2.933 0.050 Do Not Test

Ctrl MO CO vs Ctrl MO PO 4.277 0.286 1.000 Do Not Test

Ctrl MO PO vs Ecad MO CO 37.670 2.605 0.138 Do Not Test

Note: The multiple comparisons on ranks do not include an adjustment for ties.

**Kruskal-Wallis One Way Analysis of Variance on Ranks**

**For Ecad at Normalized Cell Height at 0.5 as shown in Figure 4- figure supplement 2 B1**

**Normality Test (Shapiro-Wilk):**  Failed (P < 0.050)

**Group N Missing Median 25% 75%**

Ctrl MO CO 25 0 348.604 298.120 494.203

Ctrl MO PO 24 0 345.029 284.030 356.532

Ctrl MO NO 46 0 385.890 315.754 438.802

Ecad MO CO 29 0 281.753 211.935 355.316

Ecad MO PO 13 0 349.838 281.339 412.122

Ecad MO NO 44 0 345.151 302.174 460.972

H = 19.771 with 5 degrees of freedom. (P = 0.001)

The differences in the median values among the treatment groups are greater than would be expected by chance; there is a statistically significant difference (P = 0.001)

To isolate the group or groups that differ from the others use a multiple comparison procedure.

All Pairwise Multiple Comparison Procedures (Dunn's Method) :

**Comparison Diff of Ranks Q P P<0.050**

Ctrl MO NO vs Ecad MO CO 49.934 4.019 <0.001 Yes

Ctrl MO NO vs Ctrl MO PO 23.556 1.785 1.000 No

Ctrl MO NO vs Ecad MO PO 17.194 1.045 1.000 Do Not Test

Ctrl MO NO vs Ecad MO NO 6.461 0.585 1.000 Do Not Test

Ctrl MO NO vs Ctrl MO CO 3.028 0.233 1.000 Do Not Test

Ctrl MO CO vs Ecad MO CO 46.906 3.280 0.016 Yes

Ctrl MO CO vs Ctrl MO PO 20.528 1.371 1.000 Do Not Test

Ctrl MO CO vs Ecad MO PO 14.166 0.791 1.000 Do Not Test

Ctrl MO CO vs Ecad MO NO 3.434 0.262 1.000 Do Not Test

Ecad MO NO vs Ecad MO CO 43.473 3.469 0.008 Yes

Ecad MO NO vs Ctrl MO PO 17.095 1.286 1.000 Do Not Test

Ecad MO NO vs Ecad MO PO 10.733 0.649 1.000 Do Not Test

Ecad MO PO vs Ecad MO CO 32.740 1.872 0.918 No

Ecad MO PO vs Ctrl MO PO 6.362 0.353 1.000 Do Not Test

Ctrl MO PO vs Ecad MO CO 26.378 1.824 1.000 Do Not Test

Note: The multiple comparisons on ranks do not include an adjustment for ties.

**Kruskal-Wallis One Way Analysis of Variance on Ranks**

**For Ecad at Normalized Cell Height at 0.7 as shown in Figure 4- figure supplement 2 B1**

**Normality Test (Shapiro-Wilk):**  Failed (P < 0.050)

**Group N Missing Median 25% 75%**

Ctrl MO CO 25 0 367.741 310.025 611.624

Ctrl MO PO 24 0 385.469 318.036 455.254

Ctrl MO NO 46 0 391.237 329.432 454.065

Ecad MO CO 29 0 289.054 224.012 387.721

Ecad MO PO 13 0 353.495 307.906 438.125

Ecad MO NO 44 0 378.740 332.179 482.027

H = 18.377 with 5 degrees of freedom. (P = 0.003)

The differences in the median values among the treatment groups are greater than would be expected by chance; there is a statistically significant difference (P = 0.003)

To isolate the group or groups that differ from the others use a multiple comparison procedure.

All Pairwise Multiple Comparison Procedures (Dunn's Method) :

**Comparison Diff of Ranks Q P P<0.050**

Ecad MO NO vs Ecad MO CO 49.204 3.926 0.001 Yes

Ecad MO NO vs Ecad MO PO 22.965 1.388 1.000 No

Ecad MO NO vs Ctrl MO PO 10.356 0.779 1.000 Do Not Test

Ecad MO NO vs Ctrl MO NO 6.947 0.629 1.000 Do Not Test

Ecad MO NO vs Ctrl MO CO 4.353 0.332 1.000 Do Not Test

Ctrl MO CO vs Ecad MO CO 44.851 3.137 0.026 Yes

Ctrl MO CO vs Ecad MO PO 18.612 1.039 1.000 Do Not Test

Ctrl MO CO vs Ctrl MO PO 6.003 0.401 1.000 Do Not Test

Ctrl MO CO vs Ctrl MO NO 2.594 0.199 1.000 Do Not Test

Ctrl MO NO vs Ecad MO CO 42.257 3.401 0.010 Yes

Ctrl MO NO vs Ecad MO PO 16.018 0.973 1.000 Do Not Test

Ctrl MO NO vs Ctrl MO PO 3.409 0.258 1.000 Do Not Test

Ctrl MO PO vs Ecad MO CO 38.848 2.687 0.108 No

Ctrl MO PO vs Ecad MO PO 12.609 0.699 1.000 Do Not Test

Ecad MO PO vs Ecad MO CO 26.239 1.500 1.000 Do Not Test

Note: The multiple comparisons on ranks do not include an adjustment for ties.

**Kruskal-Wallis One Way Analysis of Variance on Ranks**

**For Ecad at Normalized Cell Height at 0.9 as shown in Figure 4- figure supplement 2 B1**

**Normality Test (Shapiro-Wilk):**  Failed (P < 0.050)

**Group N Missing Median 25% 75%**

Ctrl MO CO 25 0 406.515 332.117 629.384

Ctrl MO PO 24 0 397.794 333.044 466.494

Ctrl MO NO 46 0 378.632 337.493 437.399

Ecad MO CO 29 0 289.981 219.155 377.341

Ecad MO PO 13 0 345.641 299.487 428.394

Ecad MO NO 44 0 396.953 355.485 502.866

H = 24.448 with 5 degrees of freedom. (P = <0.001)

The differences in the median values among the treatment groups are greater than would be expected by chance; there is a statistically significant difference (P = <0.001)

To isolate the group or groups that differ from the others use a multiple comparison procedure.

All Pairwise Multiple Comparison Procedures (Dunn's Method) :

**Comparison Diff of Ranks Q P P<0.050**

Ctrl MO CO vs Ecad MO CO 55.434 3.877 0.002 Yes

Ctrl MO CO vs Ecad MO PO 30.015 1.675 1.000 No

Ctrl MO CO vs Ctrl MO NO 12.922 0.993 1.000 Do Not Test

Ctrl MO CO vs Ctrl MO PO 7.983 0.533 1.000 Do Not Test

Ctrl MO CO vs Ecad MO NO 0.0818 0.00624 1.000 Do Not Test

Ecad MO NO vs Ecad MO CO 55.353 4.417 <0.001 Yes

Ecad MO NO vs Ecad MO PO 29.934 1.810 1.000 Do Not Test

Ecad MO NO vs Ctrl MO NO 12.840 1.162 1.000 Do Not Test

Ecad MO NO vs Ctrl MO PO 7.902 0.594 1.000 Do Not Test

Ctrl MO PO vs Ecad MO CO 47.451 3.282 0.015 Yes

Ctrl MO PO vs Ecad MO PO 22.032 1.221 1.000 Do Not Test

Ctrl MO PO vs Ctrl MO NO 4.938 0.374 1.000 Do Not Test

Ctrl MO NO vs Ecad MO CO 42.513 3.422 0.009 Yes

Ctrl MO NO vs Ecad MO PO 17.094 1.039 1.000 Do Not Test

Ecad MO PO vs Ecad MO CO 25.419 1.454 1.000 No

Note: The multiple comparisons on ranks do not include an adjustment for ties.

**Kruskal-Wallis One Way Analysis of Variance on Ranks**

**For Ecad at Normalized Cell Height at 0.1 as shown in Figure 4- figure supplement 2 B2**

**Normality Test (Shapiro-Wilk):**  Failed (P < 0.050)

**Group N Missing Median 25% 75%**

Ctrl MO C-C 29 0 669.652 520.227 809.276

Ctrl MO N-C 60 0 607.649 523.470 765.339

Ctrl MO N-N 51 0 631.655 555.124 772.169

Ecad MO C-C 46 0 325.150 260.779 429.143

Ecad MO N-C 54 0 442.421 355.622 534.845

Ecad MO N-N 60 0 583.217 473.053 740.476

H = 117.060 with 5 degrees of freedom. (P = <0.001)

The differences in the median values among the treatment groups are greater than would be expected by chance; there is a statistically significant difference (P = <0.001)

To isolate the group or groups that differ from the others use a multiple comparison procedure.

All Pairwise Multiple Comparison Procedures (Dunn's Method) :

**Comparison Diff of Ranks Q P P<0.050**

Ctrl MO N-N vs Ecad MO C-C 144.587 8.197 <0.001 Yes

Ctrl MO N-N vs Ecad MO N-C 97.593 5.762 <0.001 Yes

Ctrl MO N-N vs Ecad MO N-N 18.250 1.105 1.000 No

Ctrl MO N-N vs Ctrl MO N-C 9.967 0.603 1.000 Do Not Test

Ctrl MO N-N vs Ctrl MO C-C 1.241 0.0615 1.000 Do Not Test

Ctrl MO C-C vs Ecad MO C-C 143.346 6.969 <0.001 Yes

Ctrl MO C-C vs Ecad MO N-C 96.351 4.825 <0.001 Yes

Ctrl MO C-C vs Ecad MO N-N 17.009 0.867 1.000 Do Not Test

Ctrl MO C-C vs Ctrl MO N-C 8.725 0.445 1.000 Do Not Test

Ctrl MO N-C vs Ecad MO C-C 134.620 7.919 <0.001 Yes

Ctrl MO N-C vs Ecad MO N-C 87.626 5.385 <0.001 Yes

Ctrl MO N-C vs Ecad MO N-N 8.283 0.523 1.000 Do Not Test

Ecad MO N-N vs Ecad MO C-C 126.337 7.432 <0.001 Yes

Ecad MO N-N vs Ecad MO N-C 79.343 4.876 <0.001 Yes

Ecad MO N-C vs Ecad MO C-C 46.994 2.700 0.104 No

Note: The multiple comparisons on ranks do not include an adjustment for ties.

**Kruskal-Wallis One Way Analysis of Variance on Ranks**

**For Ecad at Normalized Cell Height at 0.3 as shown in Figure 4- figure supplement 2 B2**

**Normality Test (Shapiro-Wilk):**  Failed (P < 0.050)

**Group N Missing Median 25% 75%**

Ctrl MO C-C 29 0 638.424 446.610 778.995

Ctrl MO N-C 60 0 555.192 481.601 676.072

Ctrl MO N-N 51 0 549.756 461.272 685.941

Ecad MO C-C 46 0 308.600 232.669 384.813

Ecad MO N-C 54 0 396.761 321.913 489.718

Ecad MO N-N 60 0 535.059 430.385 664.417

H = 111.907 with 5 degrees of freedom. (P = <0.001)

The differences in the median values among the treatment groups are greater than would be expected by chance; there is a statistically significant difference (P = <0.001)

To isolate the group or groups that differ from the others use a multiple comparison procedure.

All Pairwise Multiple Comparison Procedures (Dunn's Method) :

**Comparison Diff of Ranks Q P P<0.050**

Ctrl MO C-C vs Ecad MO C-C 149.150 7.251 <0.001 Yes

Ctrl MO C-C vs Ecad MO N-C 99.722 4.993 <0.001 Yes

Ctrl MO C-C vs Ecad MO N-N 29.992 1.529 1.000 No

Ctrl MO C-C vs Ctrl MO N-N 12.876 0.638 1.000 Do Not Test

Ctrl MO C-C vs Ctrl MO N-C 11.259 0.574 1.000 Do Not Test

Ctrl MO N-C vs Ecad MO C-C 137.891 8.111 <0.001 Yes

Ctrl MO N-C vs Ecad MO N-C 88.463 5.437 <0.001 Yes

Ctrl MO N-C vs Ecad MO N-N 18.733 1.183 1.000 Do Not Test

Ctrl MO N-C vs Ctrl MO N-N 1.618 0.0979 1.000 Do Not Test

Ctrl MO N-N vs Ecad MO C-C 136.274 7.726 <0.001 Yes

Ctrl MO N-N vs Ecad MO N-C 86.845 5.127 <0.001 Yes

Ctrl MO N-N vs Ecad MO N-N 17.116 1.036 1.000 Do Not Test

Ecad MO N-N vs Ecad MO C-C 119.158 7.009 <0.001 Yes

Ecad MO N-N vs Ecad MO N-C 69.730 4.285 <0.001 Yes

Ecad MO N-C vs Ecad MO C-C 49.428 2.840 0.068 No

Note: The multiple comparisons on ranks do not include an adjustment for ties.

**Kruskal-Wallis One Way Analysis of Variance on Ranks**

**For Ecad at Normalized Cell Height at 0.5 as shown in Figure 4- figure supplement 2 B2**

**Normality Test (Shapiro-Wilk):**  Failed (P < 0.050)

**Group N Missing Median 25% 75%**

Ctrl MO C-C 29 0 556.697 404.627 705.923

Ctrl MO N-C 60 0 455.366 374.616 567.246

Ctrl MO N-N 51 0 436.830 380.446 573.492

Ecad MO C-C 46 0 272.197 204.701 331.927

Ecad MO N-C 54 0 325.921 279.540 425.598

Ecad MO N-N 60 0 418.222 338.058 541.031

H = 96.891 with 5 degrees of freedom. (P = <0.001)

The differences in the median values among the treatment groups are greater than would be expected by chance; there is a statistically significant difference (P = <0.001)

To isolate the group or groups that differ from the others use a multiple comparison procedure.

All Pairwise Multiple Comparison Procedures (Dunn's Method) :

**Comparison Diff of Ranks Q P P<0.050**

Ctrl MO C-C vs Ecad MO C-C 149.748 7.280 <0.001 Yes

Ctrl MO C-C vs Ecad MO N-C 99.836 4.999 <0.001 Yes

Ctrl MO C-C vs Ecad MO N-N 45.232 2.306 0.317 No

Ctrl MO C-C vs Ctrl MO N-N 29.162 1.445 1.000 Do Not Test

Ctrl MO C-C vs Ctrl MO N-C 17.649 0.900 1.000 Do Not Test

Ctrl MO N-C vs Ecad MO C-C 132.099 7.770 <0.001 Yes

Ctrl MO N-C vs Ecad MO N-C 82.187 5.051 <0.001 Yes

Ctrl MO N-C vs Ecad MO N-N 27.583 1.742 1.000 Do Not Test

Ctrl MO N-C vs Ctrl MO N-N 11.513 0.697 1.000 Do Not Test

Ctrl MO N-N vs Ecad MO C-C 120.587 6.836 <0.001 Yes

Ctrl MO N-N vs Ecad MO N-C 70.674 4.172 <0.001 Yes

Ctrl MO N-N vs Ecad MO N-N 16.071 0.973 1.000 Do Not Test

Ecad MO N-N vs Ecad MO C-C 104.516 6.148 <0.001 Yes

Ecad MO N-N vs Ecad MO N-C 54.604 3.356 0.012 Yes

Ecad MO N-C vs Ecad MO C-C 49.912 2.868 0.062 No

Note: The multiple comparisons on ranks do not include an adjustment for ties.

**Kruskal-Wallis One Way Analysis of Variance on Ranks**

**For Ecad at Normalized Cell Height at 0.7 as shown in Figure 4- figure supplement 2 B2**

**Normality Test (Shapiro-Wilk):**  Failed (P < 0.050)

**Group N Missing Median 25% 75%**

Ctrl MO C-C 29 0 430.166 324.808 517.318

Ctrl MO N-C 60 0 380.110 320.348 460.920

Ctrl MO N-N 51 0 352.687 284.923 471.208

Ecad MO C-C 46 0 229.502 181.973 280.348

Ecad MO N-C 54 0 274.275 238.059 369.161

Ecad MO N-N 60 0 329.792 254.964 443.794

H = 80.159 with 5 degrees of freedom. (P = <0.001)

The differences in the median values among the treatment groups are greater than would be expected by chance; there is a statistically significant difference (P = <0.001)

To isolate the group or groups that differ from the others use a multiple comparison procedure.

All Pairwise Multiple Comparison Procedures (Dunn's Method) :

**Comparison Diff of Ranks Q P P<0.050**

Ctrl MO C-C vs Ecad MO C-C 143.071 6.956 <0.001 Yes

Ctrl MO C-C vs Ecad MO N-C 89.014 4.457 <0.001 Yes

Ctrl MO C-C vs Ecad MO N-N 55.077 2.807 0.075 No

Ctrl MO C-C vs Ctrl MO N-N 37.330 1.850 0.964 Do Not Test

Ctrl MO C-C vs Ctrl MO N-C 17.444 0.889 1.000 Do Not Test

Ctrl MO N-C vs Ecad MO C-C 125.628 7.390 <0.001 Yes

Ctrl MO N-C vs Ecad MO N-C 71.570 4.398 <0.001 Yes

Ctrl MO N-C vs Ecad MO N-N 37.633 2.376 0.262 Do Not Test

Ctrl MO N-C vs Ctrl MO N-N 19.886 1.204 1.000 Do Not Test

Ctrl MO N-N vs Ecad MO C-C 105.741 5.995 <0.001 Yes

Ctrl MO N-N vs Ecad MO N-C 51.684 3.051 0.034 Yes

Ctrl MO N-N vs Ecad MO N-N 17.747 1.074 1.000 Do Not Test

Ecad MO N-N vs Ecad MO C-C 87.994 5.176 <0.001 Yes

Ecad MO N-N vs Ecad MO N-C 33.937 2.086 0.555 No

Ecad MO N-C vs Ecad MO C-C 54.057 3.106 0.028 Yes

Note: The multiple comparisons on ranks do not include an adjustment for ties.

**Kruskal-Wallis One Way Analysis of Variance on Ranks**

**For Ecad at Normalized Cell Height at 0.9 as shown in Figure 4- figure supplement 2 B2**

**Normality Test (Shapiro-Wilk):**  Failed (P < 0.050)

**Group N Missing Median 25% 75%**

Ctrl MO C-C 29 0 314.284 254.960 435.788

Ctrl MO N-C 60 0 297.662 249.388 344.977

Ctrl MO N-N 51 0 279.440 219.726 344.478

Ecad MO C-C 46 0 198.834 164.318 242.224

Ecad MO N-C 54 0 244.221 209.214 301.455

Ecad MO N-N 60 0 248.210 211.650 335.111

H = 55.572 with 5 degrees of freedom. (P = <0.001)

The differences in the median values among the treatment groups are greater than would be expected by chance; there is a statistically significant difference (P = <0.001)

To isolate the group or groups that differ from the others use a multiple comparison procedure.

All Pairwise Multiple Comparison Procedures (Dunn's Method) :

**Comparison Diff of Ranks Q P P<0.050**

Ctrl MO C-C vs Ecad MO C-C 127.349 6.191 <0.001 Yes

Ctrl MO C-C vs Ecad MO N-C 65.210 3.265 0.016 Yes

Ctrl MO C-C vs Ecad MO N-N 54.080 2.757 0.088 No

Ctrl MO C-C vs Ctrl MO N-N 40.492 2.007 0.671 Do Not Test

Ctrl MO C-C vs Ctrl MO N-C 19.747 1.007 1.000 Do Not Test

Ctrl MO N-C vs Ecad MO C-C 107.601 6.329 <0.001 Yes

Ctrl MO N-C vs Ecad MO N-C 45.463 2.794 0.078 No

Ctrl MO N-C vs Ecad MO N-N 34.333 2.168 0.453 Do Not Test

Ctrl MO N-C vs Ctrl MO N-N 20.745 1.256 1.000 Do Not Test

Ctrl MO N-N vs Ecad MO C-C 86.856 4.924 <0.001 Yes

Ctrl MO N-N vs Ecad MO N-C 24.718 1.459 1.000 Do Not Test

Ctrl MO N-N vs Ecad MO N-N 13.588 0.822 1.000 Do Not Test

Ecad MO N-N vs Ecad MO C-C 73.268 4.310 <0.001 Yes

Ecad MO N-N vs Ecad MO N-C 11.130 0.684 1.000 Do Not Test

Ecad MO N-C vs Ecad MO C-C 62.138 3.570 0.005 Yes

Note: The multiple comparisons on ranks do not include an adjustment for ties.

**Kruskal-Wallis One Way Analysis of Variance on Ranks**

**For Ecad at Normalized Cell Height at 0.1 as shown in Figure 4- figure supplement 2 C1**

**Normality Test (Shapiro-Wilk):**  Passed (P = 0.176)

**Equal Variance Test (Brown-Forsythe):** Passed (P = 0.181)

**Group N Missing Median 25% 75%**

Ctrl MO C-C 20 0 298.653 247.678 349.711

Ctrl MO N-C 28 0 262.521 227.396 316.153

Ctrl MO N-N 10 0 251.340 224.003 295.212

Ecad MO C-C 6 0 124.605 112.050 130.021

Ecad MO N-C 6 0 188.749 151.728 281.341

Ecad MO N-N 5 0 274.379 173.700 326.226

H = 23.655 with 5 degrees of freedom. (P = <0.001)

The differences in the median values among the treatment groups are greater than would be expected by chance; there is a statistically significant difference (P = <0.001)

To isolate the group or groups that differ from the others use a multiple comparison procedure.

All Pairwise Multiple Comparison Procedures (Dunn's Method) :

**Comparison Diff of Ranks Q P P<0.050**

Ctrl MO C-C vs Ecad MO C-C 45.933 4.528 <0.001 Yes

Ctrl MO C-C vs Ecad MO N-C 25.600 2.523 0.174 No

Ctrl MO C-C vs Ctrl MO N-N 15.600 1.848 0.969 Do Not Test

Ctrl MO C-C vs Ecad MO N-N 12.500 1.147 1.000 Do Not Test

Ctrl MO C-C vs Ctrl MO N-C 9.279 1.454 1.000 Do Not Test

Ctrl MO N-C vs Ecad MO C-C 36.655 3.739 0.003 Yes

Ctrl MO N-C vs Ecad MO N-C 16.321 1.665 1.000 Do Not Test

Ctrl MO N-C vs Ctrl MO N-N 6.321 0.787 1.000 Do Not Test

Ctrl MO N-C vs Ecad MO N-N 3.221 0.304 1.000 Do Not Test

Ecad MO N-N vs Ecad MO C-C 33.433 2.533 0.169 No

Ecad MO N-N vs Ecad MO N-C 13.100 0.993 1.000 Do Not Test

Ecad MO N-N vs Ctrl MO N-N 3.100 0.260 1.000 Do Not Test

Ctrl MO N-N vs Ecad MO C-C 30.333 2.695 0.106 Do Not Test

Ctrl MO N-N vs Ecad MO N-C 10.000 0.889 1.000 Do Not Test

Ecad MO N-C vs Ecad MO C-C 20.333 1.616 1.000 Do Not Test

Note: The multiple comparisons on ranks do not include an adjustment for ties.

**Kruskal-Wallis One Way Analysis of Variance on Ranks**

**For Ecad at Normalized Cell Height at 0.3 as shown in Figure 4- figure supplement 2 C1**

**Normality Test (Shapiro-Wilk):**  Passed (P = 0.764)

**Equal Variance Test (Brown-Forsythe):** Passed (P = 0.199)

**Group N Missing Median 25% 75%**

Ctrl MO C-C 20 0 385.432 348.258 474.505

Ctrl MO N-C 28 0 381.102 336.848 433.016

Ctrl MO N-N 10 0 372.429 327.135 384.454

Ecad MO C-C 6 0 140.935 121.154 156.987

Ecad MO N-C 6 0 235.116 212.580 314.057

Ecad MO N-N 5 0 343.535 212.001 401.920

H = 29.408 with 5 degrees of freedom. (P = <0.001)

The differences in the median values among the treatment groups are greater than would be expected by chance; there is a statistically significant difference (P = <0.001)

To isolate the group or groups that differ from the others use a multiple comparison procedure.

All Pairwise Multiple Comparison Procedures (Dunn's Method) :

**Comparison Diff of Ranks Q P P<0.050**

Ctrl MO C-C vs Ecad MO C-C 45.517 4.487 <0.001 Yes

Ctrl MO C-C vs Ecad MO N-C 33.850 3.337 0.013 Yes

Ctrl MO C-C vs Ecad MO N-N 19.350 1.776 1.000 No

Ctrl MO C-C vs Ctrl MO N-N 12.850 1.522 1.000 Do Not Test

Ctrl MO C-C vs Ctrl MO N-C 5.350 0.838 1.000 Do Not Test

Ctrl MO N-C vs Ecad MO C-C 40.167 4.097 <0.001 Yes

Ctrl MO N-C vs Ecad MO N-C 28.500 2.907 0.055 No

Ctrl MO N-C vs Ecad MO N-N 14.000 1.323 1.000 Do Not Test

Ctrl MO N-C vs Ctrl MO N-N 7.500 0.934 1.000 Do Not Test

Ctrl MO N-N vs Ecad MO C-C 32.667 2.903 0.056 No

Ctrl MO N-N vs Ecad MO N-C 21.000 1.866 0.931 Do Not Test

Ctrl MO N-N vs Ecad MO N-N 6.500 0.545 1.000 Do Not Test

Ecad MO N-N vs Ecad MO C-C 26.167 1.983 0.711 Do Not Test

Ecad MO N-N vs Ecad MO N-C 14.500 1.099 1.000 Do Not Test

Ecad MO N-C vs Ecad MO C-C 11.667 0.927 1.000 Do Not Test

Note: The multiple comparisons on ranks do not include an adjustment for ties.

**Kruskal-Wallis One Way Analysis of Variance on Ranks**

**For Ecad at Normalized Cell Height at 0.5 as shown in Figure 4- figure supplement 2 C1**

**Normality Test (Shapiro-Wilk):**  Passed (P = 0.519)

**Equal Variance Test (Brown-Forsythe):** Passed (P = 0.071)

**Group N Missing Median 25% 75%**

Ctrl MO C-C 20 0 495.340 426.359 571.216

Ctrl MO N-C 28 0 456.398 375.358 511.024

Ctrl MO N-N 10 0 418.777 388.596 450.125

Ecad MO C-C 6 0 160.288 112.530 176.789

Ecad MO N-C 6 0 303.585 250.276 334.884

Ecad MO N-N 5 0 391.082 240.227 455.245

H = 33.208 with 5 degrees of freedom. (P = <0.001)

The differences in the median values among the treatment groups are greater than would be expected by chance; there is a statistically significant difference (P = <0.001)

To isolate the group or groups that differ from the others use a multiple comparison procedure.

All Pairwise Multiple Comparison Procedures (Dunn's Method) :

**Comparison Diff of Ranks Q P P<0.050**

Ctrl MO C-C vs Ecad MO C-C 48.500 4.781 <0.001 Yes

Ctrl MO C-C vs Ecad MO N-C 37.833 3.729 0.003 Yes

Ctrl MO C-C vs Ecad MO N-N 24.600 2.257 0.360 No

Ctrl MO C-C vs Ctrl MO N-N 15.800 1.872 0.918 Do Not Test

Ctrl MO C-C vs Ctrl MO N-C 8.964 1.405 1.000 Do Not Test

Ctrl MO N-C vs Ecad MO C-C 39.536 4.032 <0.001 Yes

Ctrl MO N-C vs Ecad MO N-C 28.869 2.944 0.049 Yes

Ctrl MO N-C vs Ecad MO N-N 15.636 1.478 1.000 Do Not Test

Ctrl MO N-C vs Ctrl MO N-N 6.836 0.851 1.000 Do Not Test

Ctrl MO N-N vs Ecad MO C-C 32.700 2.905 0.055 No

Ctrl MO N-N vs Ecad MO N-C 22.033 1.958 0.754 Do Not Test

Ctrl MO N-N vs Ecad MO N-N 8.800 0.737 1.000 Do Not Test

Ecad MO N-N vs Ecad MO C-C 23.900 1.811 1.000 Do Not Test

Ecad MO N-N vs Ecad MO N-C 13.233 1.003 1.000 Do Not Test

Ecad MO N-C vs Ecad MO C-C 10.667 0.848 1.000 Do Not Test

Note: The multiple comparisons on ranks do not include an adjustment for ties.

**Kruskal-Wallis One Way Analysis of Variance on Ranks**

**For Ecad at Normalized Cell Height at 0.7 as shown in Figure 4- figure supplement 2 C1**

**Normality Test (Shapiro-Wilk):**  Passed (P = 0.903)

**Equal Variance Test (Brown-Forsythe):** Passed (P = 0.094)

**Group N Missing Median 25% 75%**

Ctrl MO C-C 20 0 550.130 505.648 618.546

Ctrl MO N-C 28 0 459.544 418.543 577.225

Ctrl MO N-N 10 0 441.268 342.301 502.646

Ecad MO C-C 6 0 165.004 117.691 176.218

Ecad MO N-C 6 0 337.747 271.203 361.538

Ecad MO N-N 5 0 424.539 273.132 475.003

H = 36.563 with 5 degrees of freedom. (P = <0.001)

The differences in the median values among the treatment groups are greater than would be expected by chance; there is a statistically significant difference (P = <0.001)

To isolate the group or groups that differ from the others use a multiple comparison procedure.

All Pairwise Multiple Comparison Procedures (Dunn's Method) :

**Comparison Diff of Ranks Q P P<0.050**

Ctrl MO C-C vs Ecad MO C-C 50.750 5.003 <0.001 Yes

Ctrl MO C-C vs Ecad MO N-C 39.917 3.935 0.001 Yes

Ctrl MO C-C vs Ecad MO N-N 28.650 2.629 0.128 No

Ctrl MO C-C vs Ctrl MO N-N 20.750 2.458 0.209 Do Not Test

Ctrl MO C-C vs Ctrl MO N-C 11.571 1.813 1.000 Do Not Test

Ctrl MO N-C vs Ecad MO C-C 39.179 3.996 <0.001 Yes

Ctrl MO N-C vs Ecad MO N-C 28.345 2.891 0.058 No

Ctrl MO N-C vs Ecad MO N-N 17.079 1.614 1.000 Do Not Test

Ctrl MO N-C vs Ctrl MO N-N 9.179 1.143 1.000 Do Not Test

Ctrl MO N-N vs Ecad MO C-C 30.000 2.666 0.115 No

Ctrl MO N-N vs Ecad MO N-C 19.167 1.703 1.000 Do Not Test

Ctrl MO N-N vs Ecad MO N-N 7.900 0.662 1.000 Do Not Test

Ecad MO N-N vs Ecad MO C-C 22.100 1.675 1.000 Do Not Test

Ecad MO N-N vs Ecad MO N-C 11.267 0.854 1.000 Do Not Test

Ecad MO N-C vs Ecad MO C-C 10.833 0.861 1.000 Do Not Test

Note: The multiple comparisons on ranks do not include an adjustment for ties.

**Kruskal-Wallis One Way Analysis of Variance on Ranks**

**For Ecad at Normalized Cell Height at 0.9 as shown in Figure 4- figure supplement 2 C1**

**Normality Test (Shapiro-Wilk):**  Passed (P = 0.307)

**Equal Variance Test (Brown-Forsythe):** Failed (P < 0.050)

**Group N Missing Median 25% 75%**

Ctrl MO C-C 20 0 503.008 437.982 587.016

Ctrl MO N-C 28 0 419.239 372.754 563.814

Ctrl MO N-N 10 0 420.042 368.795 498.907

Ecad MO C-C 6 0 152.346 136.257 169.090

Ecad MO N-C 6 0 304.425 272.712 329.528

Ecad MO N-N 5 0 392.904 278.241 422.818

H = 32.631 with 5 degrees of freedom. (P = <0.001)

The differences in the median values among the treatment groups are greater than would be expected by chance; there is a statistically significant difference (P = <0.001)

To isolate the group or groups that differ from the others use a multiple comparison procedure.

All Pairwise Multiple Comparison Procedures (Dunn's Method) :

**Comparison Diff of Ranks Q P P<0.050**

Ctrl MO C-C vs Ecad MO C-C 48.000 4.731 <0.001 Yes

Ctrl MO C-C vs Ecad MO N-C 37.167 3.664 0.004 Yes

Ctrl MO C-C vs Ecad MO N-N 25.900 2.377 0.262 No

Ctrl MO C-C vs Ctrl MO N-N 11.900 1.410 1.000 Do Not Test

Ctrl MO C-C vs Ctrl MO N-C 9.036 1.416 1.000 Do Not Test

Ctrl MO N-C vs Ecad MO C-C 38.964 3.974 0.001 Yes

Ctrl MO N-C vs Ecad MO N-C 28.131 2.869 0.062 No

Ctrl MO N-C vs Ecad MO N-N 16.864 1.594 1.000 Do Not Test

Ctrl MO N-C vs Ctrl MO N-N 2.864 0.357 1.000 Do Not Test

Ctrl MO N-N vs Ecad MO C-C 36.100 3.208 0.020 Yes

Ctrl MO N-N vs Ecad MO N-C 25.267 2.245 0.372 Do Not Test

Ctrl MO N-N vs Ecad MO N-N 14.000 1.173 1.000 Do Not Test

Ecad MO N-N vs Ecad MO C-C 22.100 1.675 1.000 No

Ecad MO N-N vs Ecad MO N-C 11.267 0.854 1.000 Do Not Test

Ecad MO N-C vs Ecad MO C-C 10.833 0.861 1.000 Do Not Test

Note: The multiple comparisons on ranks do not include an adjustment for ties.

**Kruskal-Wallis One Way Analysis of Variance on Ranks**

**For Ecad at Normalized Cell Height at 0.1 as shown in Figure 4- figure supplement 2 C2**

**Normality Test (Shapiro-Wilk):**  Passed (P = 0.588)

**Equal Variance Test (Brown-Forsythe):** Failed (P < 0.050)

**Group N Missing Median 25% 75%**

Ctrl MO CO 20 0 473.469 420.911 523.845

Ctrl MO PO 2 0 435.806 301.127 570.485

Ctrl MO NO 3 0 409.195 405.231 422.781

Ecad MO CO 8 0 161.079 147.124 172.022

Ecad MO PO 8 0 394.675 332.358 412.976

Ecad MO NO 4 0 477.290 393.174 586.200

H = 24.580 with 5 degrees of freedom. (P = <0.001)

The differences in the median values among the treatment groups are greater than would be expected by chance; there is a statistically significant difference (P = <0.001)

To isolate the group or groups that differ from the others use a multiple comparison procedure.

All Pairwise Multiple Comparison Procedures (Dunn's Method) :

**Comparison Diff of Ranks Q P P<0.050**

Ctrl MO CO vs Ecad MO CO 26.050 4.741 <0.001 Yes

Ctrl MO CO vs Ecad MO PO 11.925 2.170 0.450 No

Ctrl MO CO vs Ctrl MO NO 7.883 0.969 1.000 Do Not Test

Ctrl MO CO vs Ctrl MO PO 5.050 0.518 1.000 Do Not Test

Ctrl MO CO vs Ecad MO NO 0.550 0.0765 1.000 Do Not Test

Ecad MO NO vs Ecad MO CO 25.500 3.171 0.023 Yes

Ecad MO NO vs Ecad MO PO 11.375 1.414 1.000 Do Not Test

Ecad MO NO vs Ctrl MO NO 7.333 0.731 1.000 Do Not Test

Ecad MO NO vs Ctrl MO PO 4.500 0.396 1.000 Do Not Test

Ctrl MO PO vs Ecad MO CO 21.000 2.022 0.647 No

Ctrl MO PO vs Ecad MO PO 6.875 0.662 1.000 Do Not Test

Ctrl MO PO vs Ctrl MO NO 2.833 0.236 1.000 Do Not Test

Ctrl MO NO vs Ecad MO CO 18.167 2.043 0.616 Do Not Test

Ctrl MO NO vs Ecad MO PO 4.042 0.455 1.000 Do Not Test

Ecad MO PO vs Ecad MO CO 14.125 2.151 0.472 Do Not Test

Note: The multiple comparisons on ranks do not include an adjustment for ties.

**Kruskal-Wallis One Way Analysis of Variance on Ranks**

**For Ecad at Normalized Cell Height at 0.3 as shown in Figure 4- figure supplement 2 C2**

**Normality Test (Shapiro-Wilk):**  Passed (P = 0.564)

**Equal Variance Test (Brown-Forsythe):** Failed (P < 0.050)

**Group N Missing Median 25% 75%**

Ctrl MO CO 20 0 387.682 328.591 443.676

Ctrl MO PO 2 0 394.137 208.018 580.256

Ctrl MO NO 3 0 377.055 322.536 377.763

Ecad MO CO 8 0 143.165 121.238 172.238

Ecad MO PO 8 0 363.912 306.270 393.514

Ecad MO NO 4 0 445.555 320.364 524.362

H = 21.060 with 5 degrees of freedom. (P = <0.001)

The differences in the median values among the treatment groups are greater than would be expected by chance; there is a statistically significant difference (P = <0.001)

To isolate the group or groups that differ from the others use a multiple comparison procedure.

All Pairwise Multiple Comparison Procedures (Dunn's Method) :

**Comparison Diff of Ranks Q P P<0.050**

Ecad MO NO vs Ecad MO CO 28.250 3.512 0.007 Yes

Ecad MO NO vs Ctrl MO NO 10.083 1.005 1.000 No

Ecad MO NO vs Ecad MO PO 9.250 1.150 1.000 Do Not Test

Ecad MO NO vs Ctrl MO PO 5.750 0.506 1.000 Do Not Test

Ecad MO NO vs Ctrl MO CO 4.850 0.674 1.000 Do Not Test

Ctrl MO CO vs Ecad MO CO 23.400 4.259 <0.001 Yes

Ctrl MO CO vs Ctrl MO NO 5.233 0.644 1.000 Do Not Test

Ctrl MO CO vs Ecad MO PO 4.400 0.801 1.000 Do Not Test

Ctrl MO CO vs Ctrl MO PO 0.900 0.0924 1.000 Do Not Test

Ctrl MO PO vs Ecad MO CO 22.500 2.167 0.454 No

Ctrl MO PO vs Ctrl MO NO 4.333 0.361 1.000 Do Not Test

Ctrl MO PO vs Ecad MO PO 3.500 0.337 1.000 Do Not Test

Ecad MO PO vs Ecad MO CO 19.000 2.893 0.057 Do Not Test

Ecad MO PO vs Ctrl MO NO 0.833 0.0937 1.000 Do Not Test

Ctrl MO NO vs Ecad MO CO 18.167 2.043 0.616 Do Not Test

Note: The multiple comparisons on ranks do not include an adjustment for ties.

**Kruskal-Wallis One Way Analysis of Variance on Ranks**

**For Ecad at Normalized Cell Height at 0.5 as shown in Figure 4- figure supplement 2 C2**

**Normality Test (Shapiro-Wilk):**  Passed (P = 0.912)

**Equal Variance Test (Brown-Forsythe):** Failed (P < 0.050)

**Group N Missing Median 25% 75%**

Ctrl MO CO 20 0 273.911 205.578 350.208

Ctrl MO PO 2 0 267.110 163.468 370.752

Ctrl MO NO 3 0 317.092 246.127 327.865

Ecad MO CO 8 0 121.329 80.232 164.690

Ecad MO PO 8 0 279.418 240.780 295.764

Ecad MO NO 4 0 359.736 262.775 440.882

H = 18.626 with 5 degrees of freedom. (P = 0.002)

The differences in the median values among the treatment groups are greater than would be expected by chance; there is a statistically significant difference (P = 0.002)

To isolate the group or groups that differ from the others use a multiple comparison procedure.

All Pairwise Multiple Comparison Procedures (Dunn's Method) :

**Comparison Diff of Ranks Q P P<0.050**

Ecad MO NO vs Ecad MO CO 29.000 3.606 0.005 Yes

Ecad MO NO vs Ctrl MO PO 10.750 0.945 1.000 No

Ecad MO NO vs Ecad MO PO 9.625 1.197 1.000 Do Not Test

Ecad MO NO vs Ctrl MO CO 8.950 1.244 1.000 Do Not Test

Ecad MO NO vs Ctrl MO NO 6.417 0.640 1.000 Do Not Test

Ctrl MO NO vs Ecad MO CO 22.583 2.540 0.166 No

Ctrl MO NO vs Ctrl MO PO 4.333 0.361 1.000 Do Not Test

Ctrl MO NO vs Ecad MO PO 3.208 0.361 1.000 Do Not Test

Ctrl MO NO vs Ctrl MO CO 2.533 0.312 1.000 Do Not Test

Ctrl MO CO vs Ecad MO CO 20.050 3.649 0.004 Do Not Test

Ctrl MO CO vs Ctrl MO PO 1.800 0.185 1.000 Do Not Test

Ctrl MO CO vs Ecad MO PO 0.675 0.123 1.000 Do Not Test

Ecad MO PO vs Ecad MO CO 19.375 2.950 0.048 Do Not Test

Ecad MO PO vs Ctrl MO PO 1.125 0.108 1.000 Do Not Test

Ctrl MO PO vs Ecad MO CO 18.250 1.758 1.000 Do Not Test

Note: The multiple comparisons on ranks do not include an adjustment for ties.

**Kruskal-Wallis One Way Analysis of Variance on Ranks**

**For Ecad at Normalized Cell Height at 0.7 as shown in Figure 4- figure supplement 2 C2**

**Normality Test (Shapiro-Wilk):**  Passed (P = 0.649)

**Equal Variance Test (Brown-Forsythe):** Passed (P = 0.130)

**Group N Missing Median 25% 75%**

Ctrl MO CO 20 0 199.614 150.752 312.070

Ctrl MO PO 2 0 183.021 154.686 211.356

Ctrl MO NO 3 0 235.218 133.684 266.573

Ecad MO CO 8 0 104.259 67.625 120.639

Ecad MO PO 8 0 214.815 195.589 261.645

Ecad MO NO 4 0 287.933 220.472 348.126

H = 17.923 with 5 degrees of freedom. (P = 0.003)

The differences in the median values among the treatment groups are greater than would be expected by chance; there is a statistically significant difference (P = 0.003)

To isolate the group or groups that differ from the others use a multiple comparison procedure.

All Pairwise Multiple Comparison Procedures (Dunn's Method) :

**Comparison Diff of Ranks Q P P<0.050**

Ecad MO NO vs Ecad MO CO 29.000 3.606 0.005 Yes

Ecad MO NO vs Ctrl MO PO 15.000 1.319 1.000 No

Ecad MO NO vs Ctrl MO NO 10.833 1.080 1.000 Do Not Test

Ecad MO NO vs Ctrl MO CO 10.200 1.418 1.000 Do Not Test

Ecad MO NO vs Ecad MO PO 8.000 0.995 1.000 Do Not Test

Ecad MO PO vs Ecad MO CO 21.000 3.198 0.021 Yes

Ecad MO PO vs Ctrl MO PO 7.000 0.674 1.000 Do Not Test

Ecad MO PO vs Ctrl MO NO 2.833 0.319 1.000 Do Not Test

Ecad MO PO vs Ctrl MO CO 2.200 0.400 1.000 Do Not Test

Ctrl MO CO vs Ecad MO CO 18.800 3.422 0.009 Yes

Ctrl MO CO vs Ctrl MO PO 4.800 0.493 1.000 Do Not Test

Ctrl MO CO vs Ctrl MO NO 0.633 0.0779 1.000 Do Not Test

Ctrl MO NO vs Ecad MO CO 18.167 2.043 0.616 No

Ctrl MO NO vs Ctrl MO PO 4.167 0.348 1.000 Do Not Test

Ctrl MO PO vs Ecad MO CO 14.000 1.348 1.000 Do Not Test

Note: The multiple comparisons on ranks do not include an adjustment for ties.

**Kruskal-Wallis One Way Analysis of Variance on Ranks**

**For Ecad at Normalized Cell Height at 0.9 as shown in Figure 4- figure supplement 2 C2**

**Normality Test (Shapiro-Wilk):**  Passed (P = 0.115)

**Equal Variance Test (Brown-Forsythe):** Failed (P < 0.050)

**Group N Missing Median 25% 75%**

Ctrl MO CO 20 0 161.369 98.436 255.802

Ctrl MO PO 2 0 149.625 139.879 159.372

Ctrl MO NO 3 0 164.626 76.325 171.533

Ecad MO CO 8 0 74.696 58.010 101.293

Ecad MO PO 8 0 169.709 122.920 220.560

Ecad MO NO 4 0 233.843 203.123 261.265

H = 15.325 with 5 degrees of freedom. (P = 0.009)

The differences in the median values among the treatment groups are greater than would be expected by chance; there is a statistically significant difference (P = 0.009)

To isolate the group or groups that differ from the others use a multiple comparison procedure.

All Pairwise Multiple Comparison Procedures (Dunn's Method) :

**Comparison Diff of Ranks Q P P<0.050**

Ecad MO NO vs Ecad MO CO 27.750 3.450 0.008 Yes

Ecad MO NO vs Ctrl MO NO 16.917 1.686 1.000 No

Ecad MO NO vs Ctrl MO PO 14.750 1.297 1.000 Do Not Test

Ecad MO NO vs Ctrl MO CO 10.700 1.487 1.000 Do Not Test

Ecad MO NO vs Ecad MO PO 10.000 1.243 1.000 Do Not Test

Ecad MO PO vs Ecad MO CO 17.750 2.703 0.103 No

Ecad MO PO vs Ctrl MO NO 6.917 0.778 1.000 Do Not Test

Ecad MO PO vs Ctrl MO PO 4.750 0.457 1.000 Do Not Test

Ecad MO PO vs Ctrl MO CO 0.700 0.127 1.000 Do Not Test

Ctrl MO CO vs Ecad MO CO 17.050 3.103 0.029 Do Not Test

Ctrl MO CO vs Ctrl MO NO 6.217 0.764 1.000 Do Not Test

Ctrl MO CO vs Ctrl MO PO 4.050 0.416 1.000 Do Not Test

Ctrl MO PO vs Ecad MO CO 13.000 1.252 1.000 Do Not Test

Ctrl MO PO vs Ctrl MO NO 2.167 0.181 1.000 Do Not Test

Ctrl MO NO vs Ecad MO CO 10.833 1.218 1.000 Do Not Test

Note: The multiple comparisons on ranks do not include an adjustment for ties.

**Kruskal-Wallis One Way Analysis of Variance on Ranks**

**For Ecad at Normalized Cell Height at 0.1 as shown in Figure 4- figure supplement 2 D1**

**Normality Test (Shapiro-Wilk):**  Passed (P = 0.068)

**Equal Variance Test (Brown-Forsythe):** Passed (P = 0.078)

**Group N Missing Median 25% 75%**

Ctrl MO CO 55 0 316.853 252.299 426.299

Ctrl MO PO 10 0 311.228 253.598 399.778

Ctrl MO NO 26 0 300.300 203.795 342.880

Ecad MO CO 44 0 314.653 209.815 370.682

Ecad MO PO 33 0 369.320 320.293 410.944

Ecad MO NO 40 0 367.870 315.745 438.951

H = 22.270 with 5 degrees of freedom. (P = <0.001)

The differences in the median values among the treatment groups are greater than would be expected by chance; there is a statistically significant difference (P = <0.001)

To isolate the group or groups that differ from the others use a multiple comparison procedure.

All Pairwise Multiple Comparison Procedures (Dunn's Method) :

**Comparison Diff of Ranks Q P P<0.050**

Ecad MO NO vs Ctrl MO NO 55.919 3.688 0.003 Yes

Ecad MO NO vs Ecad MO CO 40.150 3.053 0.034 Yes

Ecad MO NO vs Ctrl MO PO 32.550 1.530 1.000 No

Ecad MO NO vs Ctrl MO CO 25.818 2.064 0.585 Do Not Test

Ecad MO NO vs Ecad MO PO 0.158 0.0111 1.000 Do Not Test

Ecad MO PO vs Ctrl MO NO 55.762 3.533 0.006 Yes

Ecad MO PO vs Ecad MO CO 39.992 2.885 0.059 No

Ecad MO PO vs Ctrl MO PO 32.392 1.491 1.000 Do Not Test

Ecad MO PO vs Ctrl MO CO 25.661 1.936 0.793 Do Not Test

Ctrl MO CO vs Ctrl MO NO 30.101 2.101 0.534 No

Ctrl MO CO vs Ecad MO CO 14.332 1.177 1.000 Do Not Test

Ctrl MO CO vs Ctrl MO PO 6.732 0.325 1.000 Do Not Test

Ctrl MO PO vs Ctrl MO NO 23.369 1.043 1.000 Do Not Test

Ctrl MO PO vs Ecad MO CO 7.600 0.360 1.000 Do Not Test

Ecad MO CO vs Ctrl MO NO 15.769 1.059 1.000 Do Not Test

Note: The multiple comparisons on ranks do not include an adjustment for ties.

**Kruskal-Wallis One Way Analysis of Variance on Ranks**

**For Ecad at Normalized Cell Height at 0.3 as shown in Figure 4- figure supplement 2 D1**

**Normality Test (Shapiro-Wilk):**  Passed (P = 0.260)

**Equal Variance Test (Brown-Forsythe):** Passed (P = 0.151)

**Group N Missing Median 25% 75%**

Ctrl MO CO 55 0 410.137 342.431 536.147

Ctrl MO PO 10 0 428.366 311.578 527.519

Ctrl MO NO 26 0 380.019 306.299 464.915

Ecad MO CO 44 0 368.395 233.221 446.011

Ecad MO PO 33 0 385.128 351.393 468.859

Ecad MO NO 40 0 461.294 391.431 535.313

H = 16.557 with 5 degrees of freedom. (P = 0.005)

The differences in the median values among the treatment groups are greater than would be expected by chance; there is a statistically significant difference (P = 0.005)

To isolate the group or groups that differ from the others use a multiple comparison procedure.

All Pairwise Multiple Comparison Procedures (Dunn's Method) :

**Comparison Diff of Ranks Q P P<0.050**

Ecad MO NO vs Ecad MO CO 49.141 3.737 0.003 Yes

Ecad MO NO vs Ctrl MO NO 34.204 2.256 0.361 No

Ecad MO NO vs Ecad MO PO 25.800 1.823 1.000 Do Not Test

Ecad MO NO vs Ctrl MO PO 21.450 1.008 1.000 Do Not Test

Ecad MO NO vs Ctrl MO CO 13.255 1.060 1.000 Do Not Test

Ctrl MO CO vs Ecad MO CO 35.886 2.948 0.048 Yes

Ctrl MO CO vs Ctrl MO NO 20.949 1.462 1.000 Do Not Test

Ctrl MO CO vs Ecad MO PO 12.545 0.947 1.000 Do Not Test

Ctrl MO CO vs Ctrl MO PO 8.195 0.396 1.000 Do Not Test

Ctrl MO PO vs Ecad MO CO 27.691 1.313 1.000 No

Ctrl MO PO vs Ctrl MO NO 12.754 0.569 1.000 Do Not Test

Ctrl MO PO vs Ecad MO PO 4.350 0.200 1.000 Do Not Test

Ecad MO PO vs Ecad MO CO 23.341 1.684 1.000 Do Not Test

Ecad MO PO vs Ctrl MO NO 8.404 0.532 1.000 Do Not Test

Ctrl MO NO vs Ecad MO CO 14.937 1.003 1.000 Do Not Test

Note: The multiple comparisons on ranks do not include an adjustment for ties.

**Kruskal-Wallis One Way Analysis of Variance on Ranks**

**For Ecad at Normalized Cell Height at 0.5 as shown in Figure 4- figure supplement 2 D1**

**Normality Test (Shapiro-Wilk):**  Passed (P = 0.307)

**Equal Variance Test (Brown-Forsythe):** Passed (P = 0.781)

**Group N Missing Median 25% 75%**

Ctrl MO CO 55 0 512.021 373.209 613.190

Ctrl MO PO 10 0 459.882 331.454 555.644

Ctrl MO NO 26 0 451.912 380.557 548.720

Ecad MO CO 44 0 352.647 256.204 465.201

Ecad MO PO 33 0 376.907 315.949 539.494

Ecad MO NO 40 0 507.451 403.688 619.726

H = 28.155 with 5 degrees of freedom. (P = <0.001)

The differences in the median values among the treatment groups are greater than would be expected by chance; there is a statistically significant difference (P = <0.001)

To isolate the group or groups that differ from the others use a multiple comparison procedure.

All Pairwise Multiple Comparison Procedures (Dunn's Method) :

**Comparison Diff of Ranks Q P P<0.050**

Ecad MO NO vs Ecad MO CO 58.530 4.451 <0.001 Yes

Ecad MO NO vs Ecad MO PO 37.969 2.683 0.110 No

Ecad MO NO vs Ctrl MO PO 20.525 0.965 1.000 Do Not Test

Ecad MO NO vs Ctrl MO NO 18.287 1.206 1.000 Do Not Test

Ecad MO NO vs Ctrl MO CO 5.284 0.422 1.000 Do Not Test

Ctrl MO CO vs Ecad MO CO 53.245 4.374 <0.001 Yes

Ctrl MO CO vs Ecad MO PO 32.685 2.466 0.205 Do Not Test

Ctrl MO CO vs Ctrl MO PO 15.241 0.737 1.000 Do Not Test

Ctrl MO CO vs Ctrl MO NO 13.002 0.908 1.000 Do Not Test

Ctrl MO NO vs Ecad MO CO 40.243 2.703 0.103 No

Ctrl MO NO vs Ecad MO PO 19.682 1.247 1.000 Do Not Test

Ctrl MO NO vs Ctrl MO PO 2.238 0.0999 1.000 Do Not Test

Ctrl MO PO vs Ecad MO CO 38.005 1.802 1.000 Do Not Test

Ctrl MO PO vs Ecad MO PO 17.444 0.803 1.000 Do Not Test

Ecad MO PO vs Ecad MO CO 20.561 1.483 1.000 Do Not Test

Note: The multiple comparisons on ranks do not include an adjustment for ties.

**Kruskal-Wallis One Way Analysis of Variance on Ranks**

**For Ecad at Normalized Cell Height at 0.7 as shown in Figure 4- figure supplement 2 D1**

**Normality Test (Shapiro-Wilk):**  Passed (P = 0.887)

**Equal Variance Test (Brown-Forsythe):** Passed (P = 0.381)

**Group N Missing Median 25% 75%**

Ctrl MO CO 55 0 527.113 429.962 614.322

Ctrl MO PO 10 0 429.421 343.278 567.054

Ctrl MO NO 26 0 498.150 411.179 590.363

Ecad MO CO 44 0 350.123 243.632 448.442

Ecad MO PO 33 0 399.668 295.091 535.131

Ecad MO NO 40 0 520.551 419.942 654.495

H = 42.480 with 5 degrees of freedom. (P = <0.001)

The differences in the median values among the treatment groups are greater than would be expected by chance; there is a statistically significant difference (P = <0.001)

To isolate the group or groups that differ from the others use a multiple comparison procedure.

All Pairwise Multiple Comparison Procedures (Dunn's Method) :

**Comparison Diff of Ranks Q P P<0.050**

Ctrl MO CO vs Ecad MO CO 67.750 5.565 <0.001 Yes

Ctrl MO CO vs Ecad MO PO 39.818 3.004 0.040 Yes

Ctrl MO CO vs Ctrl MO PO 32.077 1.550 1.000 No

Ctrl MO CO vs Ctrl MO NO 7.670 0.535 1.000 Do Not Test

Ctrl MO CO vs Ecad MO NO 0.402 0.0322 1.000 Do Not Test

Ecad MO NO vs Ecad MO CO 67.348 5.122 <0.001 Yes

Ecad MO NO vs Ecad MO PO 39.416 2.785 0.080 No

Ecad MO NO vs Ctrl MO PO 31.675 1.488 1.000 Do Not Test

Ecad MO NO vs Ctrl MO NO 7.267 0.479 1.000 Do Not Test

Ctrl MO NO vs Ecad MO CO 60.080 4.035 <0.001 Yes

Ctrl MO NO vs Ecad MO PO 32.149 2.037 0.625 Do Not Test

Ctrl MO NO vs Ctrl MO PO 24.408 1.090 1.000 Do Not Test

Ctrl MO PO vs Ecad MO CO 35.673 1.692 1.000 No

Ctrl MO PO vs Ecad MO PO 7.741 0.356 1.000 Do Not Test

Ecad MO PO vs Ecad MO CO 27.932 2.015 0.658 Do Not Test

Note: The multiple comparisons on ranks do not include an adjustment for ties.

**Kruskal-Wallis One Way Analysis of Variance on Ranks**

**For Ecad at Normalized Cell Height at 0.9 as shown in Figure 4- figure supplement 2 D1**

**Normality Test (Shapiro-Wilk):**  Passed (P = 0.651)

**Equal Variance Test (Brown-Forsythe):** Passed (P = 0.231)

**Group N Missing Median 25% 75%**

Ctrl MO CO 55 0 479.929 414.473 563.571

Ctrl MO PO 10 0 352.698 272.200 521.306

Ctrl MO NO 26 0 442.342 376.634 542.151

Ecad MO CO 44 0 296.559 216.222 387.316

Ecad MO PO 33 0 358.195 266.264 506.262

Ecad MO NO 40 0 476.898 389.261 580.052

H = 46.519 with 5 degrees of freedom. (P = <0.001)

The differences in the median values among the treatment groups are greater than would be expected by chance; there is a statistically significant difference (P = <0.001)

To isolate the group or groups that differ from the others use a multiple comparison procedure.

All Pairwise Multiple Comparison Procedures (Dunn's Method) :

**Comparison Diff of Ranks Q P P<0.050**

Ecad MO NO vs Ecad MO CO 70.595 5.369 <0.001 Yes

Ecad MO NO vs Ctrl MO PO 41.100 1.931 0.802 No

Ecad MO NO vs Ecad MO PO 39.580 2.796 0.078 Do Not Test

Ecad MO NO vs Ctrl MO NO 6.031 0.398 1.000 Do Not Test

Ecad MO NO vs Ctrl MO CO 0.405 0.0323 1.000 Do Not Test

Ctrl MO CO vs Ecad MO CO 70.191 5.766 <0.001 Yes

Ctrl MO CO vs Ctrl MO PO 40.695 1.967 0.738 Do Not Test

Ctrl MO CO vs Ecad MO PO 39.176 2.956 0.047 Do Not Test

Ctrl MO CO vs Ctrl MO NO 5.626 0.393 1.000 Do Not Test

Ctrl MO NO vs Ecad MO CO 64.565 4.337 <0.001 Yes

Ctrl MO NO vs Ctrl MO PO 35.069 1.566 1.000 Do Not Test

Ctrl MO NO vs Ecad MO PO 33.550 2.126 0.503 Do Not Test

Ecad MO PO vs Ecad MO CO 31.015 2.238 0.379 No

Ecad MO PO vs Ctrl MO PO 1.520 0.0699 1.000 Do Not Test

Ctrl MO PO vs Ecad MO CO 29.495 1.399 1.000 Do Not Test

Note: The multiple comparisons on ranks do not include an adjustment for ties.

**Kruskal-Wallis One Way Analysis of Variance on Ranks**

**For Ecad at Normalized Cell Height at 0.1 as shown in Figure 4- figure supplement 2 D2**

**Normality Test (Shapiro-Wilk):**  Passed (P = 0.612)

**Equal Variance Test (Brown-Forsythe):** Failed (P < 0.050)

**Group N Missing Median 25% 75%**

Ctrl MO C-C 54 0 521.564 458.696 594.392

Ctrl MO N-C 50 0 440.193 324.203 543.946

Ctrl MO N-N 37 0 426.512 333.773 585.828

Ecad MO C-C 35 0 271.447 183.774 321.005

Ecad MO N-C 57 0 323.379 247.372 395.603

Ecad MO N-N 40 0 454.634 355.760 542.449

H = 83.767 with 5 degrees of freedom. (P = <0.001)

The differences in the median values among the treatment groups are greater than would be expected by chance; there is a statistically significant difference (P = <0.001)

To isolate the group or groups that differ from the others use a multiple comparison procedure.

All Pairwise Multiple Comparison Procedures (Dunn's Method) :

**Comparison Diff of Ranks Q P P<0.050**

Ctrl MO C-C vs Ecad MO C-C 134.138 7.829 <0.001 Yes

Ctrl MO C-C vs Ecad MO N-C 97.712 6.517 <0.001 Yes

Ctrl MO C-C vs Ctrl MO N-C 46.012 2.969 0.045 Yes

Ctrl MO C-C vs Ctrl MO N-N 37.473 2.224 0.392 No

Ctrl MO C-C vs Ecad MO N-N 32.402 1.967 0.737 Do Not Test

Ecad MO N-N vs Ecad MO C-C 101.736 5.567 <0.001 Yes

Ecad MO N-N vs Ecad MO N-C 65.310 4.010 <0.001 Yes

Ecad MO N-N vs Ctrl MO N-C 13.610 0.813 1.000 No

Ecad MO N-N vs Ctrl MO N-N 5.072 0.282 1.000 Do Not Test

Ctrl MO N-N vs Ecad MO C-C 96.664 5.192 <0.001 Yes

Ctrl MO N-N vs Ecad MO N-C 60.238 3.614 0.005 Yes

Ctrl MO N-N vs Ctrl MO N-C 8.538 0.499 1.000 Do Not Test

Ctrl MO N-C vs Ecad MO C-C 88.126 5.065 <0.001 Yes

Ctrl MO N-C vs Ecad MO N-C 51.700 3.379 0.011 Yes

Ecad MO N-C vs Ecad MO C-C 36.426 2.148 0.475 No

Note: The multiple comparisons on ranks do not include an adjustment for ties.

**Kruskal-Wallis One Way Analysis of Variance on Ranks**

**For Ecad at Normalized Cell Height at 0.3 as shown in Figure 4- figure supplement 2 D2**

**Normality Test (Shapiro-Wilk):**  Passed (P = 0.104)

**Equal Variance Test (Brown-Forsythe):** Failed (P < 0.050)

**Group N Missing Median 25% 75%**

Ctrl MO C-C 54 0 418.848 334.481 522.648

Ctrl MO N-C 50 0 360.053 261.253 454.431

Ctrl MO N-N 37 0 367.250 270.583 519.922

Ecad MO C-C 35 0 210.299 162.330 290.494

Ecad MO N-C 57 0 284.571 213.642 362.109

Ecad MO N-N 40 0 388.684 290.975 469.960

H = 67.487 with 5 degrees of freedom. (P = <0.001)

The differences in the median values among the treatment groups are greater than would be expected by chance; there is a statistically significant difference (P = <0.001)

To isolate the group or groups that differ from the others use a multiple comparison procedure.

All Pairwise Multiple Comparison Procedures (Dunn's Method) :

**Comparison Diff of Ranks Q P P<0.050**

Ctrl MO C-C vs Ecad MO C-C 122.345 7.141 <0.001 Yes

Ctrl MO C-C vs Ecad MO N-C 76.908 5.130 <0.001 Yes

Ctrl MO C-C vs Ctrl MO N-C 32.159 2.075 0.569 No

Ctrl MO C-C vs Ecad MO N-N 25.059 1.521 1.000 Do Not Test

Ctrl MO C-C vs Ctrl MO N-N 21.800 1.294 1.000 Do Not Test

Ctrl MO N-N vs Ecad MO C-C 100.545 5.401 <0.001 Yes

Ctrl MO N-N vs Ecad MO N-C 55.109 3.306 0.014 Yes

Ctrl MO N-N vs Ctrl MO N-C 10.359 0.605 1.000 Do Not Test

Ctrl MO N-N vs Ecad MO N-N 3.259 0.181 1.000 Do Not Test

Ecad MO N-N vs Ecad MO C-C 97.286 5.324 <0.001 Yes

Ecad MO N-N vs Ecad MO N-C 51.849 3.184 0.022 Yes

Ecad MO N-N vs Ctrl MO N-C 7.100 0.424 1.000 Do Not Test

Ctrl MO N-C vs Ecad MO C-C 90.186 5.183 <0.001 Yes

Ctrl MO N-C vs Ecad MO N-C 44.749 2.925 0.052 No

Ecad MO N-C vs Ecad MO C-C 45.437 2.680 0.110 No

Note: The multiple comparisons on ranks do not include an adjustment for ties.

**Kruskal-Wallis One Way Analysis of Variance on Ranks**

**For Ecad at Normalized Cell Height at 0.5 as shown in Figure 4- figure supplement 2 D2**

**Normality Test (Shapiro-Wilk):**  Failed (P < 0.050)

**Group N Missing Median 25% 75%**

Ctrl MO C-C 54 0 334.549 277.584 448.540

Ctrl MO N-C 50 0 311.873 221.060 368.401

Ctrl MO N-N 37 0 289.108 236.327 424.401

Ecad MO C-C 35 0 170.152 148.610 234.579

Ecad MO N-C 57 0 239.153 185.941 296.182

Ecad MO N-N 40 0 263.129 191.443 373.564

H = 59.940 with 5 degrees of freedom. (P = <0.001)

The differences in the median values among the treatment groups are greater than would be expected by chance; there is a statistically significant difference (P = <0.001)

To isolate the group or groups that differ from the others use a multiple comparison procedure.

All Pairwise Multiple Comparison Procedures (Dunn's Method) :

**Comparison Diff of Ranks Q P P<0.050**

Ctrl MO C-C vs Ecad MO C-C 119.214 6.958 <0.001 Yes

Ctrl MO C-C vs Ecad MO N-C 71.940 4.798 <0.001 Yes

Ctrl MO C-C vs Ecad MO N-N 47.735 2.898 0.056 No

Ctrl MO C-C vs Ctrl MO N-C 34.245 2.210 0.407 Do Not Test

Ctrl MO C-C vs Ctrl MO N-N 19.293 1.145 1.000 Do Not Test

Ctrl MO N-N vs Ecad MO C-C 99.920 5.367 <0.001 Yes

Ctrl MO N-N vs Ecad MO N-C 52.646 3.158 0.024 Yes

Ctrl MO N-N vs Ecad MO N-N 28.442 1.579 1.000 Do Not Test

Ctrl MO N-N vs Ctrl MO N-C 14.952 0.873 1.000 Do Not Test

Ctrl MO N-C vs Ecad MO C-C 84.969 4.883 <0.001 Yes

Ctrl MO N-C vs Ecad MO N-C 37.694 2.464 0.206 No

Ctrl MO N-C vs Ecad MO N-N 13.490 0.805 1.000 Do Not Test

Ecad MO N-N vs Ecad MO C-C 71.479 3.911 0.001 Yes

Ecad MO N-N vs Ecad MO N-C 24.204 1.486 1.000 Do Not Test

Ecad MO N-C vs Ecad MO C-C 47.274 2.788 0.079 No

Note: The multiple comparisons on ranks do not include an adjustment for ties.

**Kruskal-Wallis One Way Analysis of Variance on Ranks**

**For Ecad at Normalized Cell Height at 0.7 as shown in Figure 4- figure supplement 2 D2**

**Normality Test (Shapiro-Wilk):**  Failed (P < 0.050)

**Group N Missing Median 25% 75%**

Ctrl MO C-C 54 0 282.062 217.817 375.222

Ctrl MO N-C 50 0 250.252 173.570 336.134

Ctrl MO N-N 37 0 278.885 194.555 305.639

Ecad MO C-C 35 0 151.110 120.240 229.963

Ecad MO N-C 57 0 211.229 148.645 272.548

Ecad MO N-N 40 0 208.481 135.181 280.216

H = 38.804 with 5 degrees of freedom. (P = <0.001)

The differences in the median values among the treatment groups are greater than would be expected by chance; there is a statistically significant difference (P = <0.001)

To isolate the group or groups that differ from the others use a multiple comparison procedure.

All Pairwise Multiple Comparison Procedures (Dunn's Method) :

**Comparison Diff of Ranks Q P P<0.050**

Ctrl MO C-C vs Ecad MO C-C 94.629 5.523 <0.001 Yes

Ctrl MO C-C vs Ecad MO N-C 58.684 3.914 0.001 Yes

Ctrl MO C-C vs Ecad MO N-N 58.000 3.521 0.006 Yes

Ctrl MO C-C vs Ctrl MO N-C 26.320 1.699 1.000 No

Ctrl MO C-C vs Ctrl MO N-N 24.324 1.444 1.000 Do Not Test

Ctrl MO N-N vs Ecad MO C-C 70.304 3.776 0.002 Yes

Ctrl MO N-N vs Ecad MO N-C 34.360 2.061 0.589 No

Ctrl MO N-N vs Ecad MO N-N 33.676 1.870 0.922 Do Not Test

Ctrl MO N-N vs Ctrl MO N-C 1.996 0.117 1.000 Do Not Test

Ctrl MO N-C vs Ecad MO C-C 68.309 3.926 0.001 Yes

Ctrl MO N-C vs Ecad MO N-C 32.364 2.116 0.516 Do Not Test

Ctrl MO N-C vs Ecad MO N-N 31.680 1.892 0.878 Do Not Test

Ecad MO N-N vs Ecad MO C-C 36.629 2.004 0.675 No

Ecad MO N-N vs Ecad MO N-C 0.684 0.0420 1.000 Do Not Test

Ecad MO N-C vs Ecad MO C-C 35.944 2.120 0.510 Do Not Test

Note: The multiple comparisons on ranks do not include an adjustment for ties.

**Kruskal-Wallis One Way Analysis of Variance on Ranks**

**For Ecad at Normalized Cell Height at 0.9 as shown in Figure 4- figure supplement 2 D2**

**Normality Test (Shapiro-Wilk):**  Failed (P < 0.050)

**Group N Missing Median 25% 75%**

Ctrl MO C-C 54 0 202.863 145.111 255.489

Ctrl MO N-C 50 0 207.986 128.790 280.270

Ctrl MO N-N 37 0 197.006 153.008 246.502

Ecad MO C-C 35 0 142.299 100.302 186.333

Ecad MO N-C 57 0 160.483 122.739 223.807

Ecad MO N-N 40 0 144.612 106.546 218.511

H = 23.231 with 5 degrees of freedom. (P = <0.001)

The differences in the median values among the treatment groups are greater than would be expected by chance; there is a statistically significant difference (P = <0.001)

To isolate the group or groups that differ from the others use a multiple comparison procedure.

All Pairwise Multiple Comparison Procedures (Dunn's Method) :

**Comparison Diff of Ranks Q P P<0.050**

Ctrl MO C-C vs Ecad MO C-C 64.321 3.754 0.003 Yes

Ctrl MO C-C vs Ecad MO N-N 45.828 2.782 0.081 No

Ctrl MO C-C vs Ecad MO N-C 31.409 2.095 0.543 Do Not Test

Ctrl MO C-C vs Ctrl MO N-C 4.598 0.297 1.000 Do Not Test

Ctrl MO C-C vs Ctrl MO N-N 3.075 0.183 1.000 Do Not Test

Ctrl MO N-N vs Ecad MO C-C 61.246 3.290 0.015 Yes

Ctrl MO N-N vs Ecad MO N-N 42.753 2.374 0.264 Do Not Test

Ctrl MO N-N vs Ecad MO N-C 28.334 1.700 1.000 Do Not Test

Ctrl MO N-N vs Ctrl MO N-C 1.523 0.0889 1.000 Do Not Test

Ctrl MO N-C vs Ecad MO C-C 59.723 3.432 0.009 Yes

Ctrl MO N-C vs Ecad MO N-N 41.230 2.462 0.207 Do Not Test

Ctrl MO N-C vs Ecad MO N-C 26.812 1.753 1.000 Do Not Test

Ecad MO N-C vs Ecad MO C-C 32.911 1.941 0.784 No

Ecad MO N-C vs Ecad MO N-N 14.418 0.885 1.000 Do Not Test

Ecad MO N-N vs Ecad MO C-C 18.493 1.012 1.000 Do Not Test

Note: The multiple comparisons on ranks do not include an adjustment for ties.
