# Supplementary material for: Stepwise polarisation of developing bilayered epidermis is mediated by aPKC and E-cadherin in zebrafish
Source: eLife. 2020 Jan 22;9:e49064. doi: 10.7554/eLife.49064 (PMC6975926; doi:10.7554/eLife.49064)
Supplement: Figure 5—source data 5. [file elife-49064-fig5-data5.docx]

Statistical comparisons for Lgl levels between Ctrl MO and Ecad MO at boundary Figure 5

**Color code:**

Comparisons showing high statisitcal significance

Comparisons showing weak statistical significance (p < 0.08), possibly arising due to large variations, may be biologically relevant

**Kruskal-Wallis One Way Analysis of Variance on Ranks**

**For Lgl at Normalized Cell Height at 0.1 as shown in Figure 5 B5**

**Normality Test (Shapiro-Wilk):**  Failed (P < 0.050)

**Group N Missing Median 25% 75%**

Ctrl MO C-C 47 0 456.097 309.995 611.710

Ctrl MO N-C 87 0 477.078 319.198 623.218

Ctrl MO N-N 47 0 495.646 361.836 665.920

Ecad MO C-C 59 0 606.498 469.053 833.754

Ecad MO N-C 94 0 697.157 554.303 852.933

Ecad MO N-N 45 0 654.913 558.678 888.688

H = 63.827 with 5 degrees of freedom. (P = <0.001)

The differences in the median values among the treatment groups are greater than would be expected by chance; there is a statistically significant difference (P = <0.001)

To isolate the group or groups that differ from the others use a multiple comparison procedure.

All Pairwise Multiple Comparison Procedures (Dunn's Method) :

**Comparison Diff of Ranks Q P P<0.050**

Ecad MO N-C vs Ctrl MO C-C 105.649 5.398 <0.001 Yes

Ecad MO N-C vs Ctrl MO N-C 98.847 6.065 <0.001 Yes

Ecad MO N-C vs Ctrl MO N-N 84.032 4.294 <0.001 Yes

Ecad MO N-C vs Ecad MO C-C 32.886 1.807 1.000 No

Ecad MO N-C vs Ecad MO N-N 1.234 0.0622 1.000 Do Not Test

Ecad MO N-N vs Ctrl MO C-C 104.415 4.570 <0.001 Yes

Ecad MO N-N vs Ctrl MO N-C 97.612 4.852 <0.001 Yes

Ecad MO N-N vs Ctrl MO N-N 82.798 3.624 0.004 Yes

Ecad MO N-N vs Ecad MO C-C 31.651 1.460 1.000 Do Not Test

Ecad MO C-C vs Ctrl MO C-C 72.763 3.397 0.010 Yes

Ecad MO C-C vs Ctrl MO N-C 65.961 3.570 0.005 Yes

Ecad MO C-C vs Ctrl MO N-N 51.146 2.388 0.254 No

Ctrl MO N-N vs Ctrl MO C-C 21.617 0.957 1.000 No

Ctrl MO N-N vs Ctrl MO N-C 14.815 0.747 1.000 Do Not Test

Ctrl MO N-C vs Ctrl MO C-C 6.802 0.343 1.000 Do Not Test

Note: The multiple comparisons on ranks do not include an adjustment for ties.

**Kruskal-Wallis One Way Analysis of Variance on Ranks**

**For Lgl at Normalized Cell Height at 0.3 as shown in Figure 5 B5**

**Normality Test (Shapiro-Wilk):**  Failed (P < 0.050)

**Group N Missing Median 25% 75%**

Ctrl MO C-C 47 0 605.313 408.396 823.907

Ctrl MO N-C 87 0 541.651 363.910 833.371

Ctrl MO N-N 47 0 675.285 404.258 801.602

Ecad MO C-C 59 0 826.820 634.196 1024.806

Ecad MO N-C 94 0 857.364 703.967 1048.762

Ecad MO N-N 45 0 853.951 622.114 1081.590

H = 62.439 with 5 degrees of freedom. (P = <0.001)

The differences in the median values among the treatment groups are greater than would be expected by chance; there is a statistically significant difference (P = <0.001)

To isolate the group or groups that differ from the others use a multiple comparison procedure.

All Pairwise Multiple Comparison Procedures (Dunn's Method) :

**Comparison Diff of Ranks Q P P<0.050**

Ecad MO N-C vs Ctrl MO N-C 97.804 6.001 <0.001 Yes

Ecad MO N-C vs Ctrl MO C-C 96.819 4.947 <0.001 Yes

Ecad MO N-C vs Ctrl MO N-N 89.926 4.595 <0.001 Yes

Ecad MO N-C vs Ecad MO C-C 18.267 1.004 1.000 No

Ecad MO N-C vs Ecad MO N-N 8.281 0.417 1.000 Do Not Test

Ecad MO N-N vs Ctrl MO N-C 89.523 4.450 <0.001 Yes

Ecad MO N-N vs Ctrl MO C-C 88.539 3.875 0.002 Yes

Ecad MO N-N vs Ctrl MO N-N 81.645 3.573 0.005 Yes

Ecad MO N-N vs Ecad MO C-C 9.986 0.461 1.000 Do Not Test

Ecad MO C-C vs Ctrl MO N-C 79.537 4.305 <0.001 Yes

Ecad MO C-C vs Ctrl MO C-C 78.552 3.667 0.004 Yes

Ecad MO C-C vs Ctrl MO N-N 71.659 3.346 0.012 Yes

Ctrl MO N-N vs Ctrl MO N-C 7.878 0.397 1.000 No

Ctrl MO N-N vs Ctrl MO C-C 6.894 0.305 1.000 Do Not Test

Ctrl MO C-C vs Ctrl MO N-C 0.985 0.0497 1.000 Do Not Test

Note: The multiple comparisons on ranks do not include an adjustment for ties.

**Kruskal-Wallis One Way Analysis of Variance on Ranks**

**For Lgl at Normalized Cell Height at 0.5 as shown in Figure 5 B5**

**Normality Test (Shapiro-Wilk):**  Failed (P < 0.050)

**Group N Missing Median 25% 75%**

Ctrl MO C-C 47 0 718.554 483.309 929.027

Ctrl MO N-C 87 0 616.337 400.720 890.944

Ctrl MO N-N 47 0 741.506 406.189 876.530

Ecad MO C-C 59 0 930.715 748.961 1213.047

Ecad MO N-C 94 0 974.821 758.218 1269.708

Ecad MO N-N 45 0 916.910 685.436 1227.012

H = 61.767 with 5 degrees of freedom. (P = <0.001)

The differences in the median values among the treatment groups are greater than would be expected by chance; there is a statistically significant difference (P = <0.001)

To isolate the group or groups that differ from the others use a multiple comparison procedure.

All Pairwise Multiple Comparison Procedures (Dunn's Method) :

**Comparison Diff of Ranks Q P P<0.050**

Ecad MO N-C vs Ctrl MO N-C 100.427 6.162 <0.001 Yes

Ecad MO N-C vs Ctrl MO N-N 90.394 4.619 <0.001 Yes

Ecad MO N-C vs Ctrl MO C-C 89.181 4.557 <0.001 Yes

Ecad MO N-C vs Ecad MO N-N 16.228 0.817 1.000 No

Ecad MO N-C vs Ecad MO C-C 11.998 0.659 1.000 Do Not Test

Ecad MO C-C vs Ctrl MO N-C 88.429 4.786 <0.001 Yes

Ecad MO C-C vs Ctrl MO N-N 78.395 3.660 0.004 Yes

Ecad MO C-C vs Ctrl MO C-C 77.182 3.603 0.005 Yes

Ecad MO C-C vs Ecad MO N-N 4.230 0.195 1.000 Do Not Test

Ecad MO N-N vs Ctrl MO N-C 84.199 4.186 <0.001 Yes

Ecad MO N-N vs Ctrl MO N-N 74.165 3.246 0.018 Yes

Ecad MO N-N vs Ctrl MO C-C 72.953 3.193 0.021 Yes

Ctrl MO C-C vs Ctrl MO N-C 11.247 0.567 1.000 No

Ctrl MO C-C vs Ctrl MO N-N 1.213 0.0537 1.000 Do Not Test

Ctrl MO N-N vs Ctrl MO N-C 10.034 0.506 1.000 Do Not Test

Note: The multiple comparisons on ranks do not include an adjustment for ties.

**Kruskal-Wallis One Way Analysis of Variance on Ranks**

**For Lgl at Normalized Cell Height at 0.7 as shown in Figure 5 B5**

**Normality Test (Shapiro-Wilk):**  Failed (P < 0.050)

**Group N Missing Median 25% 75%**

Ctrl MO C-C 47 0 692.886 480.451 845.832

Ctrl MO N-C 87 0 609.497 403.433 931.659

Ctrl MO N-N 47 0 691.234 384.829 848.740

Ecad MO C-C 59 0 932.192 714.482 1135.981

Ecad MO N-C 94 0 974.348 724.217 1273.475

Ecad MO N-N 45 0 903.486 694.269 1127.815

H = 56.611 with 5 degrees of freedom. (P = <0.001)

The differences in the median values among the treatment groups are greater than would be expected by chance; there is a statistically significant difference (P = <0.001)

To isolate the group or groups that differ from the others use a multiple comparison procedure.

All Pairwise Multiple Comparison Procedures (Dunn's Method) :

**Comparison Diff of Ranks Q P P<0.050**

Ecad MO N-C vs Ctrl MO C-C 93.202 4.762 <0.001 Yes

Ecad MO N-C vs Ctrl MO N-C 91.482 5.613 <0.001 Yes

Ecad MO N-C vs Ctrl MO N-N 89.926 4.595 <0.001 Yes

Ecad MO N-C vs Ecad MO N-N 16.452 0.828 1.000 No

Ecad MO N-C vs Ecad MO C-C 12.348 0.679 1.000 Do Not Test

Ecad MO C-C vs Ctrl MO C-C 80.854 3.775 0.002 Yes

Ecad MO C-C vs Ctrl MO N-C 79.134 4.283 <0.001 Yes

Ecad MO C-C vs Ctrl MO N-N 77.578 3.622 0.004 Yes

Ecad MO C-C vs Ecad MO N-N 4.104 0.189 1.000 Do Not Test

Ecad MO N-N vs Ctrl MO C-C 76.750 3.359 0.012 Yes

Ecad MO N-N vs Ctrl MO N-C 75.030 3.730 0.003 Yes

Ecad MO N-N vs Ctrl MO N-N 73.474 3.216 0.020 Yes

Ctrl MO N-N vs Ctrl MO C-C 3.277 0.145 1.000 No

Ctrl MO N-N vs Ctrl MO N-C 1.556 0.0785 1.000 Do Not Test

Ctrl MO N-C vs Ctrl MO C-C 1.720 0.0868 1.000 Do Not Test

Note: The multiple comparisons on ranks do not include an adjustment for ties.

**Kruskal-Wallis One Way Analysis of Variance on Ranks**

**For Lgl at Normalized Cell Height at 0.9 as shown in Figure 5 B5**

**Normality Test (Shapiro-Wilk):**  Failed (P < 0.050)

**Group N Missing Median 25% 75%**

Ctrl MO C-C 47 0 527.773 414.606 708.268

Ctrl MO N-C 87 0 525.480 362.477 843.975

Ctrl MO N-N 47 0 540.097 360.559 780.168

Ecad MO C-C 59 0 824.504 596.901 1011.097

Ecad MO N-C 94 0 830.036 608.098 1082.180

Ecad MO N-N 45 0 743.120 624.565 968.661

H = 62.187 with 5 degrees of freedom. (P = <0.001)

The differences in the median values among the treatment groups are greater than would be expected by chance; there is a statistically significant difference (P = <0.001)

To isolate the group or groups that differ from the others use a multiple comparison procedure.

All Pairwise Multiple Comparison Procedures (Dunn's Method) :

**Comparison Diff of Ranks Q P P<0.050**

Ecad MO N-C vs Ctrl MO C-C 104.819 5.356 <0.001 Yes

Ecad MO N-C vs Ctrl MO N-N 93.989 4.802 <0.001 Yes

Ecad MO N-C vs Ctrl MO N-C 89.502 5.492 <0.001 Yes

Ecad MO N-C vs Ecad MO N-N 16.978 0.855 1.000 No

Ecad MO N-C vs Ecad MO C-C 9.891 0.544 1.000 Do Not Test

Ecad MO C-C vs Ctrl MO C-C 94.928 4.432 <0.001 Yes

Ecad MO C-C vs Ctrl MO N-N 84.098 3.926 0.001 Yes

Ecad MO C-C vs Ctrl MO N-C 79.612 4.309 <0.001 Yes

Ecad MO C-C vs Ecad MO N-N 7.087 0.327 1.000 Do Not Test

Ecad MO N-N vs Ctrl MO C-C 87.842 3.845 0.002 Yes

Ecad MO N-N vs Ctrl MO N-N 77.012 3.371 0.011 Yes

Ecad MO N-N vs Ctrl MO N-C 72.525 3.605 0.005 Yes

Ctrl MO N-C vs Ctrl MO C-C 15.317 0.772 1.000 No

Ctrl MO N-C vs Ctrl MO N-N 4.487 0.226 1.000 Do Not Test

Ctrl MO N-N vs Ctrl MO C-C 10.830 0.479 1.000 Do Not Test

Note: The multiple comparisons on ranks do not include an adjustment for ties.

**Kruskal-Wallis One Way Analysis of Variance on Ranks**

**For Lgl at Normalized Cell Height at 0.1 as shown in Figure 5 B6**

**Normality Test (Shapiro-Wilk):**  Failed (P < 0.050)

**Group N Missing Median 25% 75%**

Ctrl MO CO 79 0 589.220 442.101 754.234

Ctrl MO PO 38 0 532.751 427.797 687.395

Ctrl MO NO 39 0 521.447 322.227 604.831

Ecad MO CO 78 0 828.715 589.531 1051.531

Ecad MO PO 37 0 693.662 345.498 866.086

Ecad MO NO 34 0 717.112 408.903 1003.674

H = 48.102 with 5 degrees of freedom. (P = <0.001)

The differences in the median values among the treatment groups are greater than would be expected by chance; there is a statistically significant difference (P = <0.001)

To isolate the group or groups that differ from the others use a multiple comparison procedure.

All Pairwise Multiple Comparison Procedures (Dunn's Method) :

**Comparison Diff of Ranks Q P P<0.050**

Ecad MO CO vs Ctrl MO NO 100.397 5.805 <0.001 Yes

Ecad MO CO vs Ctrl MO PO 83.157 4.766 <0.001 Yes

Ecad MO CO vs Ctrl MO CO 64.830 4.605 <0.001 Yes

Ecad MO CO vs Ecad MO PO 35.984 2.044 0.614 No

Ecad MO CO vs Ecad MO NO 28.669 1.582 1.000 Do Not Test

Ecad MO NO vs Ctrl MO NO 71.729 3.466 0.008 Yes

Ecad MO NO vs Ctrl MO PO 54.488 2.617 0.133 No

Ecad MO NO vs Ctrl MO CO 36.161 1.999 0.684 Do Not Test

Ecad MO NO vs Ecad MO PO 7.315 0.349 1.000 Do Not Test

Ecad MO PO vs Ctrl MO NO 64.414 3.183 0.022 Yes

Ecad MO PO vs Ctrl MO PO 47.173 2.316 0.308 Do Not Test

Ecad MO PO vs Ctrl MO CO 28.846 1.642 1.000 Do Not Test

Ctrl MO CO vs Ctrl MO NO 35.568 2.061 0.590 No

Ctrl MO CO vs Ctrl MO PO 18.327 1.053 1.000 Do Not Test

Ctrl MO PO vs Ctrl MO NO 17.241 0.858 1.000 Do Not Test

Note: The multiple comparisons on ranks do not include an adjustment for ties.

**Kruskal-Wallis One Way Analysis of Variance on Ranks**

**For Lgl at Normalized Cell Height at 0.3 as shown in Figure 5 B6**

**Normality Test (Shapiro-Wilk):**  Failed (P < 0.050)

**Group N Missing Median 25% 75%**

Ctrl MO CO 79 0 483.233 254.650 608.130

Ctrl MO PO 38 0 460.754 353.276 539.857

Ctrl MO NO 39 0 446.367 279.537 569.850

Ecad MO CO 78 0 698.262 532.156 921.498

Ecad MO PO 37 0 588.898 323.692 746.053

Ecad MO NO 34 0 519.480 361.304 868.601

H = 52.859 with 5 degrees of freedom. (P = <0.001)

The differences in the median values among the treatment groups are greater than would be expected by chance; there is a statistically significant difference (P = <0.001)

To isolate the group or groups that differ from the others use a multiple comparison procedure.

All Pairwise Multiple Comparison Procedures (Dunn's Method) :

**Comparison Diff of Ranks Q P P<0.050**

Ecad MO CO vs Ctrl MO NO 98.474 5.694 <0.001 Yes

Ecad MO CO vs Ctrl MO PO 92.094 5.279 <0.001 Yes

Ecad MO CO vs Ctrl MO CO 78.214 5.556 <0.001 Yes

Ecad MO CO vs Ecad MO PO 46.856 2.662 0.117 No

Ecad MO CO vs Ecad MO NO 43.969 2.426 0.229 Do Not Test

Ecad MO NO vs Ctrl MO NO 54.505 2.634 0.127 No

Ecad MO NO vs Ctrl MO PO 48.125 2.312 0.312 Do Not Test

Ecad MO NO vs Ctrl MO CO 34.245 1.893 0.875 Do Not Test

Ecad MO NO vs Ecad MO PO 2.887 0.138 1.000 Do Not Test

Ecad MO PO vs Ctrl MO NO 51.618 2.550 0.161 Do Not Test

Ecad MO PO vs Ctrl MO PO 45.238 2.221 0.395 Do Not Test

Ecad MO PO vs Ctrl MO CO 31.358 1.785 1.000 Do Not Test

Ctrl MO CO vs Ctrl MO NO 20.260 1.174 1.000 Do Not Test

Ctrl MO CO vs Ctrl MO PO 13.880 0.797 1.000 Do Not Test

Ctrl MO PO vs Ctrl MO NO 6.380 0.317 1.000 Do Not Test

Note: The multiple comparisons on ranks do not include an adjustment for ties.

**Kruskal-Wallis One Way Analysis of Variance on Ranks**

**For Lgl at Normalized Cell Height at 0.5 as shown in Figure 5 B6**

**Normality Test (Shapiro-Wilk):**  Failed (P < 0.050)

**Group N Missing Median 25% 75%**

Ctrl MO CO 79 0 390.244 199.066 532.872

Ctrl MO PO 38 0 371.836 286.605 470.022

Ctrl MO NO 39 0 405.267 229.424 487.201

Ecad MO CO 78 0 547.187 434.563 714.124

Ecad MO PO 37 0 474.825 250.644 572.185

Ecad MO NO 34 0 415.673 294.871 594.374

H = 42.848 with 5 degrees of freedom. (P = <0.001)

The differences in the median values among the treatment groups are greater than would be expected by chance; there is a statistically significant difference (P = <0.001)

To isolate the group or groups that differ from the others use a multiple comparison procedure.

All Pairwise Multiple Comparison Procedures (Dunn's Method) :

**Comparison Diff of Ranks Q P P<0.050**

Ecad MO CO vs Ctrl MO PO 82.735 4.742 <0.001 Yes

Ecad MO CO vs Ctrl MO NO 82.654 4.779 <0.001 Yes

Ecad MO CO vs Ctrl MO CO 76.804 5.456 <0.001 Yes

Ecad MO CO vs Ecad MO NO 59.404 3.278 0.016 Yes

Ecad MO CO vs Ecad MO PO 50.767 2.884 0.059 No

Ecad MO PO vs Ctrl MO PO 31.968 1.569 1.000 No

Ecad MO PO vs Ctrl MO NO 31.886 1.575 1.000 Do Not Test

Ecad MO PO vs Ctrl MO CO 26.037 1.482 1.000 Do Not Test

Ecad MO PO vs Ecad MO NO 8.637 0.412 1.000 Do Not Test

Ecad MO NO vs Ctrl MO PO 23.331 1.121 1.000 Do Not Test

Ecad MO NO vs Ctrl MO NO 23.250 1.124 1.000 Do Not Test

Ecad MO NO vs Ctrl MO CO 17.400 0.962 1.000 Do Not Test

Ctrl MO CO vs Ctrl MO PO 5.931 0.341 1.000 Do Not Test

Ctrl MO CO vs Ctrl MO NO 5.849 0.339 1.000 Do Not Test

Ctrl MO NO vs Ctrl MO PO 0.0816 0.00406 1.000 Do Not Test

Note: The multiple comparisons on ranks do not include an adjustment for ties.

**Kruskal-Wallis One Way Analysis of Variance on Ranks**

**For Lgl at Normalized Cell Height at 0.7 as shown in Figure 5 B6**

**Normality Test (Shapiro-Wilk):**  Failed (P < 0.050)

**Group N Missing Median 25% 75%**

Ctrl MO CO 79 0 352.960 196.367 457.906

Ctrl MO PO 38 0 338.644 268.711 451.959

Ctrl MO NO 39 0 348.993 222.101 445.441

Ecad MO CO 78 0 453.013 360.147 537.453

Ecad MO PO 37 0 368.194 237.243 449.820

Ecad MO NO 34 0 329.657 216.473 430.348

H = 26.409 with 5 degrees of freedom. (P = <0.001)

The differences in the median values among the treatment groups are greater than would be expected by chance; there is a statistically significant difference (P = <0.001)

To isolate the group or groups that differ from the others use a multiple comparison procedure.

All Pairwise Multiple Comparison Procedures (Dunn's Method) :

**Comparison Diff of Ranks Q P P<0.050**

Ecad MO CO vs Ecad MO NO 62.568 3.452 0.008 Yes

Ecad MO CO vs Ctrl MO PO 61.170 3.506 0.007 Yes

Ecad MO CO vs Ctrl MO NO 59.679 3.451 0.008 Yes

Ecad MO CO vs Ctrl MO CO 59.557 4.231 <0.001 Yes

Ecad MO CO vs Ecad MO PO 52.633 2.990 0.042 Yes

Ecad MO PO vs Ecad MO NO 9.935 0.474 1.000 No

Ecad MO PO vs Ctrl MO PO 8.537 0.419 1.000 Do Not Test

Ecad MO PO vs Ctrl MO NO 7.046 0.348 1.000 Do Not Test

Ecad MO PO vs Ctrl MO CO 6.924 0.394 1.000 Do Not Test

Ctrl MO CO vs Ecad MO NO 3.010 0.166 1.000 Do Not Test

Ctrl MO CO vs Ctrl MO PO 1.613 0.0926 1.000 Do Not Test

Ctrl MO CO vs Ctrl MO NO 0.122 0.00707 1.000 Do Not Test

Ctrl MO NO vs Ecad MO NO 2.888 0.140 1.000 Do Not Test

Ctrl MO NO vs Ctrl MO PO 1.491 0.0741 1.000 Do Not Test

Ctrl MO PO vs Ecad MO NO 1.398 0.0671 1.000 Do Not Test

Note: The multiple comparisons on ranks do not include an adjustment for ties.

**Kruskal-Wallis One Way Analysis of Variance on Ranks**

**For Lgl at Normalized Cell Height at 0.9 as shown in Figure 5 B6**

**Normality Test (Shapiro-Wilk):**  Failed (P < 0.050)

**Group N Missing Median 25% 75%**

Ctrl MO CO 79 0 287.425 168.976 408.609

Ctrl MO PO 38 0 286.238 208.528 401.033

Ctrl MO NO 39 0 326.818 182.079 414.148

Ecad MO CO 78 0 350.935 282.678 410.980

Ecad MO PO 37 0 286.228 207.473 379.454

Ecad MO NO 34 0 269.576 181.439 342.666

H = 13.403 with 5 degrees of freedom. (P = 0.020)

The differences in the median values among the treatment groups are greater than would be expected by chance; there is a statistically significant difference (P = 0.020)

To isolate the group or groups that differ from the others use a multiple comparison procedure.

All Pairwise Multiple Comparison Procedures (Dunn's Method) :

**Comparison Diff of Ranks Q P P<0.050**

Ecad MO CO vs Ecad MO NO 55.186 3.045 0.035 Yes

Ecad MO CO vs Ctrl MO CO 39.605 2.813 0.074 No

Ecad MO CO vs Ctrl MO PO 38.623 2.214 0.403 Do Not Test

Ecad MO CO vs Ecad MO PO 37.860 2.151 0.473 Do Not Test

Ecad MO CO vs Ctrl MO NO 31.423 1.817 1.000 Do Not Test

Ctrl MO NO vs Ecad MO NO 23.763 1.148 1.000 No

Ctrl MO NO vs Ctrl MO CO 8.182 0.474 1.000 Do Not Test

Ctrl MO NO vs Ctrl MO PO 7.200 0.358 1.000 Do Not Test

Ctrl MO NO vs Ecad MO PO 6.437 0.318 1.000 Do Not Test

Ecad MO PO vs Ecad MO NO 17.326 0.827 1.000 Do Not Test

Ecad MO PO vs Ctrl MO CO 1.745 0.0993 1.000 Do Not Test

Ecad MO PO vs Ctrl MO PO 0.762 0.0374 1.000 Do Not Test

Ctrl MO PO vs Ecad MO NO 16.563 0.796 1.000 Do Not Test

Ctrl MO PO vs Ctrl MO CO 0.983 0.0564 1.000 Do Not Test

Ctrl MO CO vs Ecad MO NO 15.581 0.861 1.000 Do Not Test

Note: The multiple comparisons on ranks do not include an adjustment for ties.

**Kruskal-Wallis One Way Analysis of Variance on Ranks**

**For Lgl at Normalized Cell Height at 0.1 as shown in Figure 5 C5**

**Normality Test (Shapiro-Wilk):**  Failed (P < 0.050)

**Group N Missing Median 25% 75%**

Ctrl MO C-C 68 0 551.018 420.507 764.191

Ctrl MO N-C 138 0 483.843 373.297 616.511

Ctrl MO N-N 120 0 495.763 398.459 650.624

Ecad MO C-C 95 0 829.297 663.349 1132.921

Ecad MO N-C 140 0 854.640 578.084 1128.590

Ecad MO N-N 104 0 627.910 455.131 920.587

H = 161.892 with 5 degrees of freedom. (P = <0.001)

The differences in the median values among the treatment groups are greater than would be expected by chance; there is a statistically significant difference (P = <0.001)

To isolate the group or groups that differ from the others use a multiple comparison procedure.

All Pairwise Multiple Comparison Procedures (Dunn's Method) :

**Comparison Diff of Ranks Q P P<0.050**

Ecad MO C-C vs Ctrl MO N-C 223.685 8.734 <0.001 Yes

Ecad MO C-C vs Ctrl MO N-N 223.140 8.458 <0.001 Yes

Ecad MO C-C vs Ctrl MO C-C 173.549 5.687 <0.001 Yes

Ecad MO C-C vs Ecad MO N-N 112.922 4.142 <0.001 Yes

Ecad MO C-C vs Ecad MO N-C 11.117 0.435 1.000 No

Ecad MO N-C vs Ctrl MO N-C 212.568 9.224 <0.001 Yes

Ecad MO N-C vs Ctrl MO N-N 212.023 8.871 <0.001 Yes

Ecad MO N-C vs Ctrl MO C-C 162.432 5.720 <0.001 Yes

Ecad MO N-C vs Ecad MO N-N 101.805 4.094 <0.001 Yes

Ecad MO N-N vs Ctrl MO N-C 110.763 4.440 <0.001 Yes

Ecad MO N-N vs Ctrl MO N-N 110.218 4.282 <0.001 Yes

Ecad MO N-N vs Ctrl MO C-C 60.627 2.024 0.645 No

Ctrl MO C-C vs Ctrl MO N-C 50.136 1.761 1.000 No

Ctrl MO C-C vs Ctrl MO N-N 49.591 1.701 1.000 Do Not Test

Ctrl MO N-N vs Ctrl MO N-C 0.545 0.0227 1.000 Do Not Test

Note: The multiple comparisons on ranks do not include an adjustment for ties.

**Kruskal-Wallis One Way Analysis of Variance on Ranks**

**For Lgl at Normalized Cell Height at 0.3 as shown in Figure 5 C5**

**Normality Test (Shapiro-Wilk):**  Failed (P < 0.050)

**Group N Missing Median 25% 75%**

Ctrl MO C-C 68 0 455.224 348.723 581.280

Ctrl MO N-C 138 0 411.650 304.562 523.084

Ctrl MO N-N 120 0 430.774 319.068 549.321

Ecad MO C-C 95 0 735.345 583.403 965.313

Ecad MO N-C 140 0 683.850 510.586 935.912

Ecad MO N-N 104 0 519.971 356.125 748.656

H = 175.324 with 5 degrees of freedom. (P = <0.001)

The differences in the median values among the treatment groups are greater than would be expected by chance; there is a statistically significant difference (P = <0.001)

To isolate the group or groups that differ from the others use a multiple comparison procedure.

All Pairwise Multiple Comparison Procedures (Dunn's Method) :

**Comparison Diff of Ranks Q P P<0.050**

Ecad MO C-C vs Ctrl MO N-C 244.085 9.530 <0.001 Yes

Ecad MO C-C vs Ctrl MO N-N 241.720 9.162 <0.001 Yes

Ecad MO C-C vs Ctrl MO C-C 204.912 6.715 <0.001 Yes

Ecad MO C-C vs Ecad MO N-N 141.227 5.180 <0.001 Yes

Ecad MO C-C vs Ecad MO N-C 33.173 1.299 1.000 No

Ecad MO N-C vs Ctrl MO N-C 210.911 9.152 <0.001 Yes

Ecad MO N-C vs Ctrl MO N-N 208.546 8.726 <0.001 Yes

Ecad MO N-C vs Ctrl MO C-C 171.739 6.048 <0.001 Yes

Ecad MO N-C vs Ecad MO N-N 108.054 4.345 <0.001 Yes

Ecad MO N-N vs Ctrl MO N-C 102.857 4.123 <0.001 Yes

Ecad MO N-N vs Ctrl MO N-N 100.492 3.904 0.001 Yes

Ecad MO N-N vs Ctrl MO C-C 63.685 2.126 0.503 No

Ctrl MO C-C vs Ctrl MO N-C 39.172 1.376 1.000 No

Ctrl MO C-C vs Ctrl MO N-N 36.807 1.262 1.000 Do Not Test

Ctrl MO N-N vs Ctrl MO N-C 2.365 0.0986 1.000 Do Not Test

Note: The multiple comparisons on ranks do not include an adjustment for ties.

**Kruskal-Wallis One Way Analysis of Variance on Ranks**

**For Lgl at Normalized Cell Height at 0.5 as shown in Figure 5 C5**

**Normality Test (Shapiro-Wilk):**  Failed (P < 0.050)

**Group N Missing Median 25% 75%**

Ctrl MO C-C 68 0 370.494 280.883 475.298

Ctrl MO N-C 138 0 336.381 254.854 422.615

Ctrl MO N-N 120 0 346.354 252.068 429.645

Ecad MO C-C 95 0 600.214 468.219 754.994

Ecad MO N-C 140 0 519.587 394.473 717.652

Ecad MO N-N 104 0 409.455 300.706 540.699

H = 165.345 with 5 degrees of freedom. (P = <0.001)

The differences in the median values among the treatment groups are greater than would be expected by chance; there is a statistically significant difference (P = <0.001)

To isolate the group or groups that differ from the others use a multiple comparison procedure.

All Pairwise Multiple Comparison Procedures (Dunn's Method) :

**Comparison Diff of Ranks Q P P<0.050**

Ecad MO C-C vs Ctrl MO N-N 245.616 9.310 <0.001 Yes

Ecad MO C-C vs Ctrl MO N-C 243.090 9.491 <0.001 Yes

Ecad MO C-C vs Ctrl MO C-C 206.930 6.781 <0.001 Yes

Ecad MO C-C vs Ecad MO N-N 152.454 5.592 <0.001 Yes

Ecad MO C-C vs Ecad MO N-C 48.194 1.887 0.887 No

Ecad MO N-C vs Ctrl MO N-N 197.421 8.260 <0.001 Yes

Ecad MO N-C vs Ctrl MO N-C 194.895 8.457 <0.001 Yes

Ecad MO N-C vs Ctrl MO C-C 158.736 5.590 <0.001 Yes

Ecad MO N-C vs Ecad MO N-N 104.260 4.192 <0.001 Yes

Ecad MO N-N vs Ctrl MO N-N 93.162 3.620 0.004 Yes

Ecad MO N-N vs Ctrl MO N-C 90.635 3.633 0.004 Yes

Ecad MO N-N vs Ctrl MO C-C 54.476 1.818 1.000 No

Ctrl MO C-C vs Ctrl MO N-N 38.685 1.327 1.000 No

Ctrl MO C-C vs Ctrl MO N-C 36.159 1.270 1.000 Do Not Test

Ctrl MO N-C vs Ctrl MO N-N 2.526 0.105 1.000 Do Not Test

Note: The multiple comparisons on ranks do not include an adjustment for ties.

**Kruskal-Wallis One Way Analysis of Variance on Ranks**

**For Lgl at Normalized Cell Height at 0.7 as shown in Figure 5 C5**

**Normality Test (Shapiro-Wilk):**  Failed (P < 0.050)

**Group N Missing Median 25% 75%**

Ctrl MO C-C 68 0 326.715 240.381 388.782

Ctrl MO N-C 138 0 277.278 222.543 360.185

Ctrl MO N-N 120 0 283.206 206.751 364.799

Ecad MO C-C 95 0 438.890 361.274 569.808

Ecad MO N-C 140 0 400.832 312.382 522.441

Ecad MO N-N 104 0 318.656 260.889 445.400

H = 131.148 with 5 degrees of freedom. (P = <0.001)

The differences in the median values among the treatment groups are greater than would be expected by chance; there is a statistically significant difference (P = <0.001)

To isolate the group or groups that differ from the others use a multiple comparison procedure.

All Pairwise Multiple Comparison Procedures (Dunn's Method) :

**Comparison Diff of Ranks Q P P<0.050**

Ecad MO C-C vs Ctrl MO N-N 224.270 8.501 <0.001 Yes

Ecad MO C-C vs Ctrl MO N-C 214.830 8.388 <0.001 Yes

Ecad MO C-C vs Ctrl MO C-C 169.881 5.567 <0.001 Yes

Ecad MO C-C vs Ecad MO N-N 133.923 4.912 <0.001 Yes

Ecad MO C-C vs Ecad MO N-C 42.608 1.669 1.000 No

Ecad MO N-C vs Ctrl MO N-N 181.662 7.601 <0.001 Yes

Ecad MO N-C vs Ctrl MO N-C 172.221 7.473 <0.001 Yes

Ecad MO N-C vs Ctrl MO C-C 127.273 4.482 <0.001 Yes

Ecad MO N-C vs Ecad MO N-N 91.315 3.672 0.004 Yes

Ecad MO N-N vs Ctrl MO N-N 90.347 3.510 0.007 Yes

Ecad MO N-N vs Ctrl MO N-C 80.906 3.243 0.018 Yes

Ecad MO N-N vs Ctrl MO C-C 35.958 1.200 1.000 No

Ctrl MO C-C vs Ctrl MO N-N 54.389 1.865 0.932 No

Ctrl MO C-C vs Ctrl MO N-C 44.949 1.579 1.000 Do Not Test

Ctrl MO N-C vs Ctrl MO N-N 9.441 0.394 1.000 Do Not Test

Note: The multiple comparisons on ranks do not include an adjustment for ties.

**Kruskal-Wallis One Way Analysis of Variance on Ranks**

**For Lgl at Normalized Cell Height at 0.9 as shown in Figure 5 C5**

**Normality Test (Shapiro-Wilk):**  Failed (P < 0.050)

**Group N Missing Median 25% 75%**

Ctrl MO C-C 68 0 275.220 208.209 320.932

Ctrl MO N-C 138 0 231.037 181.157 294.037

Ctrl MO N-N 120 0 224.837 170.277 291.581

Ecad MO C-C 95 0 339.502 268.025 412.711

Ecad MO N-C 140 0 327.203 254.521 414.443

Ecad MO N-N 104 0 269.400 227.269 357.485

H = 102.525 with 5 degrees of freedom. (P = <0.001)

The differences in the median values among the treatment groups are greater than would be expected by chance; there is a statistically significant difference (P = <0.001)

To isolate the group or groups that differ from the others use a multiple comparison procedure.

All Pairwise Multiple Comparison Procedures (Dunn's Method) :

**Comparison Diff of Ranks Q P P<0.050**

Ecad MO C-C vs Ctrl MO N-N 193.309 7.327 <0.001 Yes

Ecad MO C-C vs Ctrl MO N-C 173.796 6.786 <0.001 Yes

Ecad MO C-C vs Ctrl MO C-C 120.219 3.939 0.001 Yes

Ecad MO C-C vs Ecad MO N-N 85.975 3.153 0.024 Yes

Ecad MO C-C vs Ecad MO N-C 15.935 0.624 1.000 No

Ecad MO N-C vs Ctrl MO N-N 177.374 7.422 <0.001 Yes

Ecad MO N-C vs Ctrl MO N-C 157.861 6.850 <0.001 Yes

Ecad MO N-C vs Ctrl MO C-C 104.284 3.672 0.004 Yes

Ecad MO N-C vs Ecad MO N-N 70.040 2.816 0.073 No

Ecad MO N-N vs Ctrl MO N-N 107.334 4.170 <0.001 Yes

Ecad MO N-N vs Ctrl MO N-C 87.821 3.520 0.006 Yes

Ecad MO N-N vs Ctrl MO C-C 34.244 1.143 1.000 No

Ctrl MO C-C vs Ctrl MO N-N 73.090 2.507 0.183 No

Ctrl MO C-C vs Ctrl MO N-C 53.577 1.882 0.897 Do Not Test

Ctrl MO N-C vs Ctrl MO N-N 19.513 0.814 1.000 Do Not Test

Note: The multiple comparisons on ranks do not include an adjustment for ties.

**Kruskal-Wallis One Way Analysis of Variance on Ranks**

**For Lgl at Normalized Cell Height at 0.1 as shown in Figure 5 C6**

**Normality Test (Shapiro-Wilk):**  Failed (P < 0.050)

**Group N Missing Median 25% 75%**

Ctrl MO CO 73 0 521.418 373.426 716.705

Ctrl MO PO 47 0 571.986 434.393 779.919

Ctrl MO NO 74 0 573.532 399.496 695.992

Ecad MO CO 108 0 860.529 595.699 1048.479

Ecad MO PO 47 0 723.990 565.800 1001.315

Ecad MO NO 71 0 721.841 544.491 940.994

H = 69.170 with 5 degrees of freedom. (P = <0.001)

The differences in the median values among the treatment groups are greater than would be expected by chance; there is a statistically significant difference (P = <0.001)

To isolate the group or groups that differ from the others use a multiple comparison procedure.

All Pairwise Multiple Comparison Procedures (Dunn's Method) :

**Comparison Diff of Ranks Q P P<0.050**

Ecad MO CO vs Ctrl MO CO 118.625 6.450 <0.001 Yes

Ecad MO CO vs Ctrl MO NO 111.422 6.083 <0.001 Yes

Ecad MO CO vs Ctrl MO PO 96.810 4.564 <0.001 Yes

Ecad MO CO vs Ecad MO NO 33.034 1.781 1.000 No

Ecad MO CO vs Ecad MO PO 21.714 1.024 1.000 Do Not Test

Ecad MO PO vs Ctrl MO CO 96.910 4.269 <0.001 Yes

Ecad MO PO vs Ctrl MO NO 89.707 3.962 0.001 Yes

Ecad MO PO vs Ctrl MO PO 75.096 2.999 0.041 Yes

Ecad MO PO vs Ecad MO NO 11.320 0.496 1.000 Do Not Test

Ecad MO NO vs Ctrl MO CO 85.591 4.230 <0.001 Yes

Ecad MO NO vs Ctrl MO NO 78.388 3.887 0.002 Yes

Ecad MO NO vs Ctrl MO PO 63.776 2.794 0.078 No

Ctrl MO PO vs Ctrl MO CO 21.815 0.961 1.000 No

Ctrl MO PO vs Ctrl MO NO 14.612 0.645 1.000 Do Not Test

Ctrl MO NO vs Ctrl MO CO 7.203 0.360 1.000 Do Not Test

Note: The multiple comparisons on ranks do not include an adjustment for ties.

**Kruskal-Wallis One Way Analysis of Variance on Ranks**

**For Lgl at Normalized Cell Height at 0.3 as shown in Figure 5 C6**

**Normality Test (Shapiro-Wilk):**  Failed (P < 0.050)

**Group N Missing Median 25% 75%**

Ctrl MO CO 73 0 623.740 491.962 863.588

Ctrl MO PO 47 0 624.592 512.134 811.286

Ctrl MO NO 74 0 638.266 482.597 746.292

Ecad MO CO 108 0 1016.728 735.370 1290.409

Ecad MO PO 47 0 833.463 698.015 1218.552

Ecad MO NO 71 0 857.661 660.358 1152.350

H = 88.611 with 5 degrees of freedom. (P = <0.001)

The differences in the median values among the treatment groups are greater than would be expected by chance; there is a statistically significant difference (P = <0.001)

To isolate the group or groups that differ from the others use a multiple comparison procedure.

All Pairwise Multiple Comparison Procedures (Dunn's Method) :

**Comparison Diff of Ranks Q P P<0.050**

Ecad MO CO vs Ctrl MO NO 133.233 7.273 <0.001 Yes

Ecad MO CO vs Ctrl MO PO 123.811 5.837 <0.001 Yes

Ecad MO CO vs Ctrl MO CO 119.079 6.474 <0.001 Yes

Ecad MO CO vs Ecad MO NO 31.956 1.723 1.000 No

Ecad MO CO vs Ecad MO PO 31.460 1.483 1.000 Do Not Test

Ecad MO PO vs Ctrl MO NO 101.773 4.495 <0.001 Yes

Ecad MO PO vs Ctrl MO PO 92.351 3.688 0.003 Yes

Ecad MO PO vs Ctrl MO CO 87.619 3.860 0.002 Yes

Ecad MO PO vs Ecad MO NO 0.496 0.0217 1.000 Do Not Test

Ecad MO NO vs Ctrl MO NO 101.277 5.022 <0.001 Yes

Ecad MO NO vs Ctrl MO PO 91.855 4.024 <0.001 Yes

Ecad MO NO vs Ctrl MO CO 87.123 4.306 <0.001 Yes

Ctrl MO CO vs Ctrl MO NO 14.154 0.707 1.000 No

Ctrl MO CO vs Ctrl MO PO 4.732 0.208 1.000 Do Not Test

Ctrl MO PO vs Ctrl MO NO 9.422 0.416 1.000 Do Not Test

Note: The multiple comparisons on ranks do not include an adjustment for ties.

**Kruskal-Wallis One Way Analysis of Variance on Ranks**

**For Lgl at Normalized Cell Height at 0.5 as shown in Figure 5 C6**

**Normality Test (Shapiro-Wilk):**  Failed (P < 0.050)

**Group N Missing Median 25% 75%**

Ctrl MO CO 73 0 749.979 581.497 964.280

Ctrl MO PO 47 0 682.562 511.236 779.744

Ctrl MO NO 74 0 644.092 499.041 800.063

Ecad MO CO 108 0 1102.294 855.556 1484.716

Ecad MO PO 47 0 871.479 669.953 1205.502

Ecad MO NO 71 0 859.094 694.596 1264.737

H = 100.827 with 5 degrees of freedom. (P = <0.001)

The differences in the median values among the treatment groups are greater than would be expected by chance; there is a statistically significant difference (P = <0.001)

To isolate the group or groups that differ from the others use a multiple comparison procedure.

All Pairwise Multiple Comparison Procedures (Dunn's Method) :

**Comparison Diff of Ranks Q P P<0.050**

Ecad MO CO vs Ctrl MO NO 154.202 8.418 <0.001 Yes

Ecad MO CO vs Ctrl MO PO 149.299 7.038 <0.001 Yes

Ecad MO CO vs Ctrl MO CO 104.260 5.669 <0.001 Yes

Ecad MO CO vs Ecad MO PO 56.331 2.656 0.119 No

Ecad MO CO vs Ecad MO NO 42.339 2.283 0.337 Do Not Test

Ecad MO NO vs Ctrl MO NO 111.863 5.547 <0.001 Yes

Ecad MO NO vs Ctrl MO PO 106.960 4.686 <0.001 Yes

Ecad MO NO vs Ctrl MO CO 61.921 3.060 0.033 Yes

Ecad MO NO vs Ecad MO PO 13.992 0.613 1.000 Do Not Test

Ecad MO PO vs Ctrl MO NO 97.871 4.323 <0.001 Yes

Ecad MO PO vs Ctrl MO PO 92.968 3.713 0.003 Yes

Ecad MO PO vs Ctrl MO CO 47.929 2.111 0.521 No

Ctrl MO CO vs Ctrl MO NO 49.942 2.494 0.189 No

Ctrl MO CO vs Ctrl MO PO 45.039 1.984 0.709 Do Not Test

Ctrl MO PO vs Ctrl MO NO 4.903 0.217 1.000 Do Not Test

Note: The multiple comparisons on ranks do not include an adjustment for ties.

**Kruskal-Wallis One Way Analysis of Variance on Ranks**

**For Lgl at Normalized Cell Height at 0.7 as shown in Figure 5 C6**

**Normality Test (Shapiro-Wilk):**  Failed (P < 0.050)

**Group N Missing Median 25% 75%**

Ctrl MO CO 73 0 810.464 612.192 994.223

Ctrl MO PO 47 0 689.408 527.089 779.744

Ctrl MO NO 74 0 600.907 487.146 803.427

Ecad MO CO 108 0 1046.509 806.419 1465.251

Ecad MO PO 47 0 834.841 669.953 1196.851

Ecad MO NO 71 0 844.694 653.116 1203.574

H = 93.913 with 5 degrees of freedom. (P = <0.001)

The differences in the median values among the treatment groups are greater than would be expected by chance; there is a statistically significant difference (P = <0.001)

To isolate the group or groups that differ from the others use a multiple comparison procedure.

All Pairwise Multiple Comparison Procedures (Dunn's Method) :

**Comparison Diff of Ranks Q P P<0.050**

Ecad MO CO vs Ctrl MO NO 157.186 8.581 <0.001 Yes

Ecad MO CO vs Ctrl MO PO 139.759 6.589 <0.001 Yes

Ecad MO CO vs Ctrl MO CO 82.131 4.465 <0.001 Yes

Ecad MO CO vs Ecad MO PO 60.280 2.842 0.067 No

Ecad MO CO vs Ecad MO NO 43.607 2.351 0.281 Do Not Test

Ecad MO NO vs Ctrl MO NO 113.579 5.632 <0.001 Yes

Ecad MO NO vs Ctrl MO PO 96.152 4.212 <0.001 Yes

Ecad MO NO vs Ctrl MO CO 38.524 1.904 0.854 No

Ecad MO NO vs Ecad MO PO 16.673 0.730 1.000 Do Not Test

Ecad MO PO vs Ctrl MO NO 96.906 4.280 <0.001 Yes

Ecad MO PO vs Ctrl MO PO 79.479 3.174 0.023 Yes

Ecad MO PO vs Ctrl MO CO 21.851 0.963 1.000 Do Not Test

Ctrl MO CO vs Ctrl MO NO 75.055 3.748 0.003 Yes

Ctrl MO CO vs Ctrl MO PO 57.628 2.538 0.167 No

Ctrl MO PO vs Ctrl MO NO 17.428 0.770 1.000 No

Note: The multiple comparisons on ranks do not include an adjustment for ties.

**Kruskal-Wallis One Way Analysis of Variance on Ranks**

**For Lgl at Normalized Cell Height at 0.9 as shown in Figure 5 C6**

**Normality Test (Shapiro-Wilk):**  Failed (P < 0.050)

**Group N Missing Median 25% 75%**

Ctrl MO CO 73 0 716.425 552.911 852.416

Ctrl MO PO 47 0 616.049 481.803 733.746

Ctrl MO NO 74 0 546.594 428.611 725.357

Ecad MO CO 108 0 882.597 642.375 1152.141

Ecad MO PO 47 0 732.931 507.098 971.898

Ecad MO NO 71 0 730.684 556.568 1013.321

H = 55.926 with 5 degrees of freedom. (P = <0.001)

The differences in the median values among the treatment groups are greater than would be expected by chance; there is a statistically significant difference (P = <0.001)

To isolate the group or groups that differ from the others use a multiple comparison procedure.

All Pairwise Multiple Comparison Procedures (Dunn's Method) :

**Comparison Diff of Ranks Q P P<0.050**

Ecad MO CO vs Ctrl MO NO 123.927 6.765 <0.001 Yes

Ecad MO CO vs Ctrl MO PO 104.797 4.940 <0.001 Yes

Ecad MO CO vs Ctrl MO CO 54.433 2.960 0.046 Yes

Ecad MO CO vs Ecad MO PO 49.000 2.310 0.313 No

Ecad MO CO vs Ecad MO NO 35.263 1.901 0.859 Do Not Test

Ecad MO NO vs Ctrl MO NO 88.664 4.397 <0.001 Yes

Ecad MO NO vs Ctrl MO PO 69.534 3.046 0.035 Yes

Ecad MO NO vs Ctrl MO CO 19.170 0.947 1.000 No

Ecad MO NO vs Ecad MO PO 13.736 0.602 1.000 Do Not Test

Ecad MO PO vs Ctrl MO NO 74.928 3.309 0.014 Yes

Ecad MO PO vs Ctrl MO PO 55.798 2.228 0.388 No

Ecad MO PO vs Ctrl MO CO 5.434 0.239 1.000 Do Not Test

Ctrl MO CO vs Ctrl MO NO 69.494 3.470 0.008 Yes

Ctrl MO CO vs Ctrl MO PO 50.364 2.219 0.398 Do Not Test

Ctrl MO PO vs Ctrl MO NO 19.130 0.845 1.000 No

Note: The multiple comparisons on ranks do not include an adjustment for ties.
