# Supplementary material for: Stepwise polarisation of developing bilayered epidermis is mediated by aPKC and E-cadherin in zebrafish
Source: eLife. 2020 Jan 22;9:e49064. doi: 10.7554/eLife.49064 (PMC6975926; doi:10.7554/eLife.49064)
Supplement: Supplementary file 1. [file elife-49064-supp1.pdf]

```
# Supplementary File 1- R Script for Figure 1, Figure 1- figure supplement 4
#
#
# This is a program to calculate various parameters for E-cadherin quantifications-----
#
#
#
# "rawdata" contains data read from the csv file(provided by user). It should contain the following
columns:
#   1) Genotype
#   2) Cell name
#   3) Mean
#   4) Length
#   5) Boundary -- If you are doing cell wise analysis(put same letter in the whole column in case
analysis is cell wise and by boundaries)
#
#
# "stack.all" contains stack wise calculated data which includes:
#   1) Height at a particular stack
#   2) Normalized Height
#   3) Normalized Ecad
#   4) Absolute Ecad at each stack
#
# "cell.all" contains cell wise data. Which includes the following:
#   1) The total Height of the Cell
#   2) Total Ecad in the cell
#
# "genotype.all" contains  genotype wise data which includes the following:
#   1) Mean apical perimeter
#   2) Median apical perimeter
#   3) Mean Total Ecad per cell
#   4) Median Total Ecad per cell
#   5) Mean Height of the cell
#   6) Median Height of the cell
#   7) First quantile of Height of cell
#   8) Third quantile of height of cell
#
# "locfit.model" contains the locfit data and the height at which the levels were predicted.
#
# "apical.perimeter" contains the apical most stack used for apical perimeter analysis
#
#
#
#

#clears memory--------
rm(list = ls())
```

```
#sets a working directory-----------
#This is where all your graphs will be saved

setwd(
  "C:/Users/prata/Desktop/Test for paper/"
)

#libraries required for the program to work.-----------------
# List of libraries required for the program to run
pkg <- c("scales", "locfit","tidyr","purrr","dplyr","ggplot2","broom",
         "data.table", "RCurl","stringr", "grid", "gridExtra", "RColorBrewer")

# Check if the libraries are installed and install libraries not present
new.pkg <- pkg[!(pkg %in% installed.packages())]
if (length(new.pkg)) {
  install.packages(new.pkg)
}
rm("pkg","new.pkg")

#load libraries in the memory
library(scales)
library(locfit)
library(tidyr)
library(purrr)
library(dplyr)
library(ggplot2)
library(data.table)
library(RCurl)
library(stringr)
library(grid)
library(gridExtra)
library(RColorBrewer)

#stops warnings from being displayed
options(warn = -1)



# read raw data------------------
# the file should should have the following components:
# Genotype        Cell name        Area      Mean    Boundary

#Folder and file names where your input file is saved

Folderadd <-
  "C:/Users/prata/Desktop/Test for paper/"
filename <-
  "Figure 1- source data 1"
```

```
format <- ".csv"
fulladdress <- paste(Folderadd, filename, format, sep = "")

rawdata <-
  read.csv(fulladdress,
           header = TRUE,
           sep = ",") %>% na.omit()

# data arranging--------------------

#To have Genotype and Cell name, Boundary order as in the file (Otherwise it comes alphabetically
which may not be desirable)
rawdata$Genotype <-
  factor(rawdata$Genotype, levels = unique(rawdata$Genotype))
rawdata$Cell.name <-
  factor(rawdata$Cell.name, levels = unique(rawdata$Cell.name))
rawdata$Boundary <-
  factor(rawdata$Boundary, levels = unique(rawdata$Boundary))


#group by genotype and cell name calculate absolute ecad for each stack, and nest the data to to
help calculations on groups
by_cellname <- rawdata %>%
  group_by(Genotype, Cell.name) %>%
  mutate(AbsoluteEcad = Mean * Area) %>%
  nest()


#function to find height
rawheight <- function(df) {
  height <- vector("double", nrow(df))
  for (i in seq_along(df$Mean)) {
    height[i] <- (i - 1) * 0.28 #the height of the z stack
  }
  height
}


#function to normalize height
normheightfun <- function(df) {
  nheight <- vector("double", nrow(df))
  maxh <- nrow(df)
  minh <- 1
  for (i in seq_along(df$Mean)) {
    nheight[i] <- ((i) - minh) / (maxh - minh)
  }
  nheight
}
```

```
#function to normalize ecad levels
normecadfun <- function(df) {
  necad <- vector("double", nrow(df))
  maxe <- max(df$Mean)
  mine <- min(df$Mean)
  for (i in seq_along(df$Mean)) {
    necad[i] <- (df$Mean[i] - mine) * 100 / (maxe - mine)
  }
  necad
}

#dataframe which now has height per stack, normalized height, normalized ecad per genotype-cell
name
stack.all <- by_cellname %>%
  mutate(
    Stackheight = data %>% map(rawheight),
    Normalizedheight = data %>% map(normheightfun),
    NormalizedEcad = data %>% map(normecadfun),
    Uniquecellname = paste(Genotype, Cell.name)
  ) %>% unnest()


#to know the total ecad in a cell and height of a cell
cell.all <-  stack.all %>%
  group_by(Genotype, Cell.name) %>%
  summarize(Total_Ecad = sum(AbsoluteEcad),
            Height_of_cell = max(Stackheight))

#to fit linear regression to find the abnormal polarized distribution-----------------

#function for linear regression

#locfit -------------------------------------------
# locfit will fit the data cell wise and predict values at given cell heights

#nest the data in stack.all with normalized height to use in locfit for prediction
locfit.input <- stack.all %>%
  group_by(Genotype, Cell.name) %>%
  nest()

# locfit.input$data[[1]]


#cell heights chosen to interpolate ecad levels
predictvalues <- c(0.1, 0.3, 0.5, 0.7, 0.9)

#To calculate the y-axis limits in locfit graphs
```

```
ylimmax <- max(rawdata$Mean) + 250
ylimmin <- min(rawdata$Mean) - 100


#function to fit locfit
locfit_model <- function(df) {
  fit <- locfit(Mean ~ Normalizedheight, data = df)
  plot(
    fit,
    get.data = TRUE,
    ylim = c(ylimmin, ylimmax),
    xlim = c(0, 1),
    xlab = "Normalized Cell Height",
    ylab = "Ecad Level(in AU)"
  )
  par(new = TRUE)   #To superimpose graphs
  predictions <-  predict(fit, predictvalues)

}

#to plot the locfit graphs
#to start a new graph page and close all old ones,
# so that new graphs don't get superimposed on the old ones
plot.new()
dev.off()
plot.new()




#add locfit data to grouped cells and add cell height(on which prediction is made) to each cell
locfit.model <- locfit.input %>%
  mutate(fitvalues = data %>% map(locfit_model)) %>%
  unnest(fitvalues) %>%
  group_by(Genotype, Cell.name) %>%
  mutate(Cell_height = predictvalues)
locfit.model <- locfit.model %>%  group_by(Genotype, Cell.name)

#to save graph for locfit data
dev.copy(
  svg,
  file = paste0(filename, "_locfitgraphs.svg"),
  width = 4,
  height = 4
)

dev.off ()
```

```
#for creating groups for each cell for ggplot graph of lines ---------------------------------------------------

graph.lines <- stack.all %>% mutate (Uniquecellname = paste(Genotype, Cell.name))


#to find out the apical perimeter of the cell

apical.perimeter <- graph.lines %>%
  group_by(Genotype, Cell.name) %>%
  slice(1) %>% select(Genotype, Cell.name, Length)

cell.all <- cell.all %>% full_join(apical.perimeter, by = c("Genotype", "Cell.name"))

cell.all <- rename(cell.all, "Apical Perimeter" = Length)


# to calculate Genotype wise parameters-------------------------
genotype.peri <-  apical.perimeter %>%   group_by(Genotype) %>%
  summarise(Mean_peri = mean(Length),
            Median_peri = median(Length))


genotype.summary <- cell.all %>% group_by(Genotype) %>%
  summarise(
    Mean_TotalEcad = mean(Total_Ecad),
    Median_TotalEcad = median(Total_Ecad),
    Mean_Heightofcell = mean(Height_of_cell),
    Median_Heightofcell = median(Height_of_cell),
    First_quantile_Height = quantile(Height_of_cell, prob = 0.25),
    Third_quantile_Height = quantile(Height_of_cell, prob = 0.75),
    count = n()
  )

#final dataframe for all calculated parameters by merging the above two data frames
genotype.all <- genotype.peri %>% full_join(genotype.summary, by = "Genotype")

#writing csv-------------------------------------------------------


#write csv with height, normalized height,absolute ecad in each stack
write.csv(
  stack.all,
  file = paste0(filename, "_stackwise_calculations.csv"),
  row.names = FALSE
)

#write csv with cell level parameters
```

```
write.csv(
  cell.all,
  file = paste0(filename, "_cellwise_calculations.csv"),
  row.names = FALSE
)

#write csv with locfit fitdata
write.csv(locfit.model,
          file = paste0(filename, "_locfitdata.csv"),
          row.names = FALSE)

#write csv with genotype level parameters
write.csv(
  genotype.all,
  file = paste0(filename, "_genotypewise_calculations.csv"),
  row.names = FALSE
)

#graphs-------------------------------------------------------------

#to plot boxplots for Total Ecad vs Genotype-------------
p <-
  ggplot(data = cell.all, aes(x = Genotype, y = Total_Ecad, fill = Genotype)) +
  theme(
    plot.title = element_text(hjust = 0.5),
    text = element_text(size = 14),
    legend.position = "none",
    axis.text.x = element_text(angle = 45, hjust = 1)
  ) +
  labs(title = "Total E-cadherin", x = "Genotype", y = "E-cadherin Levels(in AU)") +
  scale_y_continuous(labels = comma) +
  theme(
    plot.title = element_text(hjust = 0.5),
    legend.position = "none",
    text = element_text(size = 14)
  ) +
  geom_boxplot(outlier.shape = NA, alpha = 0.7) +
  geom_point(position = position_jitter(width = .1),
             shape = 21,
             alpha = 0.4)#+
# coord_cartesian(ylim = c(0, 800000))
print(p)
dev.copy(
  svg,
  file = paste0(filename, "_Total Ecad.svg"),
  width = 4,
  height = 4
)
```

```
dev.off ()



#to plot boxplots for Height of cell vs Genotype--------------
q <-
  q <-
  ggplot(data = cell.all, aes(x = Genotype, y = Height_of_cell, fill = Genotype)) +
  theme(
    plot.title = element_text(hjust = 0.5),
    text = element_text(size = 14),
    legend.position = "none",
    axis.text.x = element_text(angle = 45, hjust = 1)
  ) +
  labs(title = "Height of Cells",
       x = "Genotype",
       y = expression(paste("Height(in ", mu, "m)"))) +
  scale_y_continuous(labels = comma) +
  theme(
    plot.title = element_text(hjust = 0.5),
    legend.position = "none",
    text = element_text(size = 14)
  ) +
  geom_boxplot(outlier.shape = NA, alpha = 0.7) +
  geom_point(
    position = position_jitterdodge(
      jitter.height = 0,
      jitter.width = 0.5,
      dodge.width = .1
    ),
    shape = 21,
    alpha = 0.4
  )#+
# coord_cartesian(ylim = c(0, 8))
print(q)
dev.copy(
  svg,
  file = paste0(filename, "_totalcellheight.svg"),
  width = 4,
  height = 4
)
dev.off ()

#to plot boxplots for Height of cell vs locfit data------------------
r <-
  ggplot(data = locfit.model,
         aes(
           x = factor(Cell_height),
```

```
        y = fitvalues,
        fill = Genotype
      )) +
  theme(plot.title = element_text(hjust = 0.5), text = element_text(size =
                                                                      14)) +
  labs(title = "E-cadherin levels at various Cell Heights", x = "Normalized Height", y = "E-cadherin
Levels(in AU)") +
  geom_boxplot(position = position_dodge(width = 0.8), outlier.shape = NA) +
  scale_y_continuous(labels = comma) +
  geom_point(
    alpha = 0.4,
    position = position_jitterdodge(jitter.width = .2, dodge.width = 0.8),
    shape = 21
  )#+
# coord_cartesian(ylim = c(0, 1750))
print(r)
dev.copy(
  svg,
  file = paste0(filename, "_locfit_boxplot.svg"),
  width = 4,
  height = 4
)
dev.off ()

#to plot lineplots for cell height  vs Ecad levels------------------
s <-
  ggplot(
    data = graph.lines,
    aes(
      x = Stackheight,
      y = Mean,
      group = Uniquecellname,
      fill = Genotype,
      color = Genotype
    )
  ) +
  theme(plot.title = element_text(hjust = 0.5), text = element_text(size =
                                                                      14)) +
  labs(title = "Height vs Ecad Levels",
       x = "Height(in " ~ (mu * m),
       y = "E-cadherin Levels(in AU)") +
  scale_y_continuous(labels = comma) +
  geom_point(position = position_jitter(width = .1),
             shape = 21,
             alpha = 0.4) +
  geom_line(alpha = 0.4)#+
# coord_cartesian(ylim = c(0, 1750))
print(s)
```

```
dev.copy(
  svg,
  file = paste0(filename, "_Rawheight.svg"),
  width = 4,
  height = 4
)
dev.off ()

#to plot lineplots for Normalized cell height  vs Ecad levels----------------
u <-
  ggplot(
    data = graph.lines,
    aes(
      x = Normalizedheight,
      y = Mean,
      group = Uniquecellname,
      fill = Genotype,
      color = Genotype
    )
  ) +
  theme(plot.title = element_text(hjust = 0.5), text = element_text(size =
                                                                      14)) +
  labs(title = "Normalized Height vs Ecad Levels", x = "Normalized Height", y = "E-cadherin Levels(in
AU)") +
  geom_point(shape = 21, alpha = 0.4) +
  scale_y_continuous(labels = comma) +
  geom_line(alpha = 0.4) +
  #coord_cartesian(ylim = c(0, 1750))#+
  facet_grid(facets = . ~ Boundary)
print(u)

dev.copy(
  svg,
  file = paste0(filename, "_Normalized_height.svg"),
  width = 4,
  height = 4
)
dev.off ()


#to plot lineplots for Normalized cell height  vs Normalized Ecad levels------------
t <-
  ggplot(
    data = graph.lines,
    aes(
      x = Normalizedheight,
      y = NormalizedEcad,
      group = Uniquecellname,
```

```
      fill = Genotype
      # color = Genotype
    )
  ) +
  theme(
    plot.title = element_text(hjust = 0.5),
    text = element_text(size = 14),
    legend.position = "none",
    axis.text.x = element_text(angle = 45, hjust = 1)
  ) +
  scale_y_continuous(labels = comma) +
  labs(title = "E-Cadherin Distribution", x = "Normalized Height", y = "Normalized E-cadherin Levels")
+
  # stat_smooth(aes(
  #   x = Normalizedheight,
  #   y = NormalizedEcad,
  #   group = Genotype,
  #   fill = Genotype,
  #   color = Genotype
  # ),method='lm')+
  geom_bin2d(bins = 15, alpha = 0.3) +
  geom_point(shape = 21, alpha = 0.15) + facet_grid(. ~ Boundary) #+

# stat_density_2d(aes(colour = Genotype), geom="density_2d", alpha= 0.2)
print(t)

dev.copy(
  svg,
  file = paste0(filename, "_Normalized height Normalized Ecad.svg"),
  width = 4,
  height = 4
)
dev.off ()



#to plot apical perimeter vs genotype------------
b <-
  ggplot(data = apical.perimeter, aes(x = Genotype, y = Length, fill = Genotype)) +
  theme(
    plot.title = element_text(hjust = 0.5),
    text = element_text(size = 14),
    legend.position = "none",
    axis.text.x = element_text(angle = 45, hjust = 1)
  ) +
  labs(title = "Apical Perimeter of Cells",
       x = "Genotype",
       y = "Length(in" ~ (mu * m)) +
```

```
  geom_boxplot(outlier.shape = NA, alpha = 0.7) +
  scale_y_continuous(labels = comma) +
  theme(
    plot.title = element_text(hjust = 0.5),
    legend.position = "none",
    text = element_text(size = 14)
  ) +
  geom_point(position = position_jitter(width = .1),
             shape = 21,
             alpha = 0.4)#+
# coord_cartesian(ylim = c(40, 120))
print(b)
dev.copy(
  svg,
  file = paste0(filename, "_apical perimeter.svg"),
  width = 4,
  height = 4
)
dev.off ()



# multiplot graphs-------------------------------------
# To plot multiple graphs in one page

grid.newpage()
pushViewport(viewport(layout = grid.layout(17, 6)))
vplayout <- function(x, y)
  viewport(layout.pos.row = x, layout.pos.col = y)
print(r, vp = vplayout(1:6, 2:6))
print(p, vp = vplayout(7:11, 1:2))
print(q, vp = vplayout(7:11, 3:4))
print(b, vp = vplayout(7:11, 5:6))
print(t, vp = vplayout(12:17, 2:5))
dev.copy(
  svg,
  file = paste0(filename, "multiplot_all.svg"),
  width = 10,
  height = 10
)
dev.off()
```

```
# Supplementary File 2- R Script for Figure 2, 3, Figure 2-figure supplement 3, 4
#
#
# This is a program to calculate various parameters for E-cadherin quantifications-----
#
#
#
# "rawdata" contains data read from the csv file(provided by user). It should contain the following columns:
#   1) Genotype
#   2) Cell name
#   3) Mean
#   4) Length
#   5) Boundary -- If you are doing cell wise analysis(put same letter in the whole column in case analysis is cell wise and by boundaries)
#
#
# "stack.all" contains stack wise calculated data which includes:
#   1) Height at a particular stack
#   2) Normalized Height
#   3) Normalized Ecad
#   4) Absolute Ecad at each stack
#
# "cell.all" contains cell wise data. Which includes the following:
#   1) The total Height of the Cell
#   2) Total Ecad in the cell
#
# "genotype.all" contains  genotype wise data which includes the following:
#   1) Mean apical perimeter
#   2) Median apical perimeter
#   3) Mean Total Ecad per cell
#   4) Median Total Ecad per cell
#   5) Mean Height of the cell
#   6) Median Height of the cell
#   7) First quantile of Height of cell
#   8) Third quantile of height of cell
#
# "locfit.model" contains the locfit data and the height at which the levels were predicted.
#
# "apical.perimeter" contains the apical most stack used for apical perimeter analysis
#
# "linear.reg.noise"  contains number of cells/percentage cells showing abnormal polarized distribution
#
# "linear.reg.whole" contains linear regression for both siblings and mutants
#
# The program also classifies the cells according to the cell height.-- Tall cells, medium cells, short cells
```

```
#
#
#

#clears memory--------
rm(list = ls())

#sets a working directory-----------
#This is where all your graphs will be saved

setwd(
  "C:/User/Desktop/"
)

#libraries required for the program to work.-----------------
# List of libraries required for the program to run
pkg <- c("scales", "locfit","tidyr","purrr","dplyr","ggplot2","broom",
         "data.table", "RCurl","stringr", "grid", "gridExtra", "RColorBrewer")

# Check if the libraries are installed and install libraries not present
new.pkg <- pkg[!(pkg %in% installed.packages())]
if (length(new.pkg)) {
  install.packages(new.pkg)
}
rm("pkg","new.pkg")

#load libraries in the memory
library(scales)
library(locfit)
library(tidyr)
library(purrr)
library(dplyr)
library(ggplot2)
library(data.table)
library(RCurl)
library(stringr)
library(grid)
library(gridExtra)
library(RColorBrewer)

#stops warnings from being displayed
options(warn = -1)



# read raw data------------------
# the file should should have the following components:
# Genotype        Cell name         Area      Mean    Boundary
```

```
#Folder and file names where your input file is saved

Folderadd <-
  "C:/User/Desktop/"
filename <-
  "aPKC mut"
format <- ".csv"
fulladdress <- paste(Folderadd, filename, format, sep = "")

rawdata <-
  read.csv(fulladdress,
           header = TRUE,
           sep = ",") %>% na.omit()

# data arranging--------------------

#To have Genotype and Cell name, Boundary order as in the file (Otherwise it comes alphabetically
which may not be desirable)
rawdata$Genotype <-
  factor(rawdata$Genotype, levels = unique(rawdata$Genotype))
rawdata$Cell.name <-
  factor(rawdata$Cell.name, levels = unique(rawdata$Cell.name))
rawdata$Boundary <-
  factor(rawdata$Boundary, levels = unique(rawdata$Boundary))


#group by genotype and cell name calculate absolute ecad for each stack, and nest the data to to
help calculations on groups
by_cellname <- rawdata %>%
  group_by(Genotype, Cell.name) %>%
  mutate(AbsoluteEcad = Mean * Area) %>%
  nest()


#function to find height
rawheight <- function(df) {
  height <- vector("double", nrow(df))
  for (i in seq_along(df$Mean)) {
    height[i] <- (i - 1) * 0.28 #the height of the z stack
  }
  height
}


#function to normalize height
normheightfun <- function(df) {
  nheight <- vector("double", nrow(df))
```

```
  maxh <- nrow(df)
  minh <- 1
  for (i in seq_along(df$Mean)) {
    nheight[i] <- ((i) - minh) / (maxh - minh)
  }
  nheight
}

#function to normalize ecad levels
normecadfun <- function(df) {
  necad <- vector("double", nrow(df))
  maxe <- max(df$Mean)
  mine <- min(df$Mean)
  for (i in seq_along(df$Mean)) {
    necad[i] <- (df$Mean[i] - mine) * 100 / (maxe - mine)
  }
  necad
}

#dataframe which now has height per stack, normalized height, normalized ecad per genotype-cell
name
stack.all <- by_cellname %>%
  mutate(
    Stackheight = data %>% map(rawheight),
    Normalizedheight = data %>% map(normheightfun),
    NormalizedEcad = data %>% map(normecadfun),
    Uniquecellname = paste(Genotype, Cell.name)
  ) %>% unnest()


#to know the total ecad in a cell and height of a cell
cell.all <-  stack.all %>%
  group_by(Genotype, Cell.name) %>%
  summarize(Total_Ecad = sum(AbsoluteEcad),
            Height_of_cell = max(Stackheight))

#to fit linear regression to find the abnormal polarized distribution-----------------

#function for linear regression

linearfit.noise <- function(df) {

  # To give specific colors in linear regressions, a column called colour1 can be added to input file.
  # all "colourcodes" lines can then be uncommented.

  # colourcodes <- df %>% distinct(colour1)
  # colourcodes <- as.character(colourcodes[['colour1']])
  # print(colourcodes)
```

```
# select sibling data for linear regression

df.lm <- df %>% filter(str_detect(df$Genotype, "sib"))

# fit linear regression on the sibling data
fit <- lm(NormalizedEcad ~ Normalizedheight, data = df.lm)

# find the upper and lower prediction limits at 99% prediction level
pred <-
  as.data.frame(predict(fit, df, level = 0.99, interval = "prediction"))

# check if the data is within the prediction limits
# TRUE= point is within the predction limit
# FALSE = point lies outside the prediction limit

dat <-
  data.frame(df,
             Distribution = df$NormalizedEcad <= pred$upr &
              df$NormalizedEcad >= pred$lwr)

# Replace TRUE with normal and FALSE with Abnormal

dat <- dat %>%  mutate(Distribution=replace(Distribution, Distribution=="TRUE", "Normal"))
dat <- dat %>%  mutate(Distribution=replace(Distribution, Distribution=="FALSE", "Abnormal"))

# plot graph for the linear regression fit data showing the prediction limits according to the siblings

plot.lm <-
  ggplot(data = dat, aes(
    x = dat$Normalizedheight,
    y = dat$NormalizedEcad
  )) +
  # scale_colour_manual(values = colourcodes) +
  coord_cartesian(ylim = c(0, 100)) +
  theme(
    plot.title = element_text(hjust = 0.5),
    text = element_text(size = 14),
    axis.text.x = element_text(angle = 45, hjust = 1)
  ) +
  labs(title = "E-Cadherin Distribution", x = "Normalized Height", y = "Normalized E-cadherin Levels")
+
  stat_smooth(method = 'lm',
              # color = colourcodes[1],
              se = FALSE
  )  +
  geom_ribbon(
```

```
      data = pred,
      aes(ymin = lwr, ymax = upr),
      # fill = colourcodes[1],
      alpha = 0.2
    ) +
    geom_point(aes(colour = Genotype), alpha = 0.4)
  print(plot.lm)


  # save the plot
  dev.copy(svg,
           file = paste0(
             filename,
             "_",
             df$Genotype[1],
             "_linear_regrs_sibling_pred_interval.svg"
           ))
  dev.off()

  # Calculate abnormal vs normal distribution numbers and percentage
  dat.summary <-
    dat %>% group_by(Genotype, Distribution) %>% distinct(Uniquecellname)  %>%
arrange(Distribution)
  dat.summary <-
    dat.summary %>% ungroup() %>% group_by(Genotype, Uniquecellname) %>% slice(1)
  dat.summary <-
    dat.summary %>% group_by(Genotype, Distribution) %>% summarize(Count =
                                                                     n())
  dat.summary2 <-
    dat.summary %>% ungroup() %>% group_by(Genotype) %>% summarize(total =
                                                                     sum(Count))
  dat.summary3 <- full_join(dat.summary, dat.summary2)
  dat.summary3 <-
    dat.summary3 %>% mutate(Percentage = ((Count / total) * 100))
  dat.summary3
}
# show_col(hue_pal()(4)) #to show color codes

# Group according the boundary types to get values for boudnaries only (eg. Clone-Clone)
linear.reg.input <-  stack.all %>%
  group_by(Boundary) %>%
  nest()

# to get table for all normal vs abnormal distribution according to boundary type
linear.reg.noise <- linear.reg.input %>%
  mutate(lmfit = data %>% map(linearfit.noise)) %>% select(-data)  %>% unnest(lmfit)

write.csv(
```

```
  linear.reg.noise,
  file = paste0(filename, "_abnormal_by_linear_regression.csv"),
  row.names = FALSE
)


# function to check if linear regressions are close by in both mutants and siblings

linearfit.whole <- function(df) {
  # colourcodes <- df %>% distinct(colour1)
  # colourcodes <- as.character(colourcodes[['colour1']])

  # plot regressions for bith siblings and mutants
  plot.lm <-
    ggplot(data = df,
           aes(
             x = df$Normalizedheight,
             y = df$NormalizedEcad,
             colour = Genotype
           )) +
    # scale_colour_manual(values = colourcodes) +
    coord_cartesian(ylim = c(0, 100)) +
    theme(
      plot.title = element_text(hjust = 0.5),
      text = element_text(size = 14),
      axis.text.x = element_text(angle = 45, hjust = 1)
    ) +
    labs(title = "E-Cadherin Distribution", x = "Normalized Height", y = "Normalized E-cadherin Levels")
+
    stat_smooth(method = 'lm', se = FALSE)  +
    geom_point(aes(), alpha = 0.4)
  print(plot.lm)

  # save the plot
  dev.copy(svg, file = paste0(filename, df$Genotype[1], "_linear_regrs_whole.svg"))
  dev.off()

  # fit linear regression in both sibling and mutant

  fit <- lm(NormalizedEcad ~ Normalizedheight, data = df)
  radjsq <- summary(fit)$adj.r.squared
  rsq <- summary(fit)$r.squared
  slope <- coef(summary(fit))["Normalizedheight", "Estimate"]
  intercept <- coef(summary(fit))["(Intercept)", "Estimate"]
  tablefit <- data.frame(radjsq, rsq, intercept, slope)
  tablefit <-
    tablefit %>% rename("Adjusted R Squared" = radjsq, "R Squared" = rsq)
  tablefit
```

```r
}

# Make table according with linear regressions fit according to boundary types

linear.reg.whole <- linear.reg.input %>%
  mutate(lmfit = data %>% map(linearfit.whole)) %>% select(-data)  %>% unnest(lmfit)

#write csv for linear fit for Normalized ecad and normalized height--------------------

write.csv(
  linear.reg.whole,
  file = paste0(filename, "_genotypewise_linearfit_coefficients.csv"),
  row.names = FALSE
)


#locfit ------------------------------------------
# locfit will fit the data cell wise and predict values at given cell heights

#nest the data in stack.all with normalized height to use in locfit for prediction
locfit.input <- stack.all %>%
  group_by(Genotype, Cell.name) %>%
  nest()

# locfit.input$data[[1]]


#cell heights chosen to interpolate ecad levels
predictvalues <- c(0.1, 0.3, 0.5, 0.7, 0.9)

#To calculate the y-axis limits in locfit graphs
ylimmax <- max(rawdata$Mean) + 250
ylimmin <- min(rawdata$Mean) - 100


#function to fit locfit
locfit_model <- function(df) {
  fit <- locfit(Mean ~ Normalizedheight, data = df)
  plot(
    fit,
    get.data = TRUE,
    ylim = c(ylimmin, ylimmax),
    xlim = c(0, 1),
    xlab = "Normalized Cell Height",
    ylab = "Ecad Level(in AU)"
  )
  par(new = TRUE)   #To superimpose graphs
  predictions <-  predict(fit, predictvalues)
```

```
}

#to plot the locfit graphs
#to start a new graph page and close all old ones,
# so that new graphs don't get superimposed on the old ones
plot.new()
dev.off()
plot.new()




#add locfit data to grouped cells and add cell height(on which prediction is made) to each cell
locfit.model <- locfit.input %>%
  mutate(fitvalues = data %>% map(locfit_model)) %>%
  unnest(fitvalues) %>%
  group_by(Genotype, Cell.name) %>%
  mutate(Cell_height = predictvalues)
locfit.model <- locfit.model %>%  group_by(Genotype, Cell.name)

#to save graph for locfit data
dev.copy(
  svg,
  file = paste0(filename, "_locfitgraphs.svg"),
  width = 4,
  height = 4
)

dev.off ()


#for creating groups for each cell for ggplot graph of lines -------------------------------------------------

graph.lines <- stack.all %>% mutate (Uniquecellname = paste(Genotype, Cell.name))


#to find out the apical perimeter of the cell

apical.perimeter <- graph.lines %>%
  group_by(Genotype, Cell.name) %>%
  slice(1) %>% select(Genotype, Cell.name, Length)

cell.all <- cell.all %>% full_join(apical.perimeter, by = c("Genotype", "Cell.name"))

cell.all <- rename(cell.all, "Apical Perimeter" = Length)
```

```
# to calculate Genotype wise parameters-------------------------
genotype.peri <-  apical.perimeter %>%   group_by(Genotype) %>%
  summarise(Mean_peri = mean(Length),
            Median_peri = median(Length))


genotype.summary <- cell.all %>% group_by(Genotype) %>%
  summarise(
    Mean_TotalEcad = mean(Total_Ecad),
    Median_TotalEcad = median(Total_Ecad),
    Mean_Heightofcell = mean(Height_of_cell),
    Median_Heightofcell = median(Height_of_cell),
    First_quantile_Height = quantile(Height_of_cell, prob = 0.25),
    Third_quantile_Height = quantile(Height_of_cell, prob = 0.75),
    count = n()
  )

#final dataframe for all calculated parameters by merging the above two data frames
genotype.all <- genotype.peri %>% full_join(genotype.summary, by = "Genotype")

#filter out tall cells or short cells--------------------------

# This part of the program will not run if the following names are in the name of the file
# (so that the same program can be run on different hieghted cells without really re- filtering the
cells according to the height again)
namecontains <- c("tall_cells", "short_cells","median_cells")

if (!any(str_detect(filename, namecontains))) {


  #filter tall cells
  sibthirdquantile <- genotype.summary %>% filter(str_detect(genotype.summary$Genotype, "sib"))
%>% select(Third_quantile_Height)
  sibfirstquantile <- genotype.summary %>% filter(str_detect(genotype.summary$Genotype, "sib"))
%>% select(First_quantile_Height)

  tall_cells_cellwise <- cell.all %>%
    full_join(genotype.all, by = "Genotype") %>% filter(Height_of_cell >
sibthirdquantile$Third_quantile_Height) %>% select(Genotype, Cell.name, Total_Ecad,
Height_of_cell)

  tall_cells_all <-  stack.all %>%
    semi_join(tall_cells_cellwise, by = c("Genotype", "Cell.name"))


  #write csv with tall cells filtered out
  write.csv(
    tall_cells_cellwise,
```

```
    file = paste0(filename, "_tall_cells_cellwise.csv"),
    row.names = FALSE
  )
  write.csv(
    tall_cells_all,
    file = paste0(filename, "_tall_cells_all.csv"),
    row.names = FALSE
  )

  #filter short cells
  short_cells_cellwise <- cell.all %>%
    full_join(genotype.all, by = "Genotype") %>% filter(Height_of_cell <
sibfirstquantile$First_quantile_Height) %>% select(Genotype, Cell.name, Total_Ecad, Height_of_cell)

  short_cells_all <-  stack.all %>%
    semi_join(short_cells_cellwise, by = c("Genotype", "Cell.name"))
  #write csv with short cells filtered out
  write.csv(
    short_cells_cellwise,
    file = paste0(filename, "_short_cells_cellwise.csv"),
    row.names = FALSE
  )
  write.csv(
    short_cells_all,
    file = paste0(filename, "_short_cells_all.csv"),
    row.names = FALSE
  )


  #filter median heighted cells
  median_cells_cellwise <- cell.all %>%
    full_join(genotype.all, by = "Genotype") %>% filter(Height_of_cell >=
sibfirstquantile$First_quantile_Height & Height_of_cell <= sibthirdquantile$Third_quantile_Height)
%>% select(Genotype, Cell.name, Total_Ecad, Height_of_cell)

  median_cells_all <-  stack.all %>%
    semi_join(median_cells_cellwise, by = c("Genotype", "Cell.name"))

  #write csv with short cells filtered out
  write.csv(
    median_cells_cellwise,
    file = paste0(filename, "_median_cells_cellwise.csv"),
    row.names = FALSE
  )
  write.csv(
    median_cells_all,
    file = paste0(filename, "_median_cells_all.csv"),
    row.names = FALSE
```

```
  )

}

#writing csv-------------------------------------------------------


#write csv with height, normalized height,absolute ecad in each stack
write.csv(
  stack.all,
  file = paste0(filename, "_stackwise_calculations.csv"),
  row.names = FALSE
)

#write csv with cell level parameters
write.csv(
  cell.all,
  file = paste0(filename, "_cellwise_calculations.csv"),
  row.names = FALSE
)

#write csv with locfit fitdata
write.csv(locfit.model,
          file = paste0(filename, "_locfitdata.csv"),
          row.names = FALSE)

#write csv with genotype level parameters
write.csv(
  genotype.all,
  file = paste0(filename, "_genotypewise_calculations.csv"),
  row.names = FALSE
)

#graphs-----------------------------------------------------------

#to plot boxplots for Total Ecad vs Genotype-------------
p <-
  ggplot(data = cell.all, aes(x = Genotype, y = Total_Ecad, fill = Genotype)) +
  theme(
    plot.title = element_text(hjust = 0.5),
    text = element_text(size = 14),
    legend.position = "none",
    axis.text.x = element_text(angle = 45, hjust = 1)
  ) +
  labs(title = "Total E-cadherin", x = "Genotype", y = "E-cadherin Levels(in AU)") +
  scale_y_continuous(labels = comma) +
  theme(
    plot.title = element_text(hjust = 0.5),
```

```
    legend.position = "none",
    text = element_text(size = 14)
  ) +
  geom_boxplot(outlier.shape = NA, alpha = 0.7) +
  geom_point(position = position_jitter(width = .1),
             shape = 21,
             alpha = 0.4)#+
# coord_cartesian(ylim = c(0, 800000))
print(p)
dev.copy(
  svg,
  file = paste0(filename, "_Total Ecad.svg"),
  width = 4,
  height = 4
)
dev.off ()



#to plot boxplots for Height of cell vs Genotype--------------
q <-
  q <-
  ggplot(data = cell.all, aes(x = Genotype, y = Height_of_cell, fill = Genotype)) +
  theme(
    plot.title = element_text(hjust = 0.5),
    text = element_text(size = 14),
    legend.position = "none",
    axis.text.x = element_text(angle = 45, hjust = 1)
  ) +
  labs(title = "Height of Cells",
       x = "Genotype",
       y = expression(paste("Height(in ", mu, "m)"))) +
  scale_y_continuous(labels = comma) +
  theme(
    plot.title = element_text(hjust = 0.5),
    legend.position = "none",
    text = element_text(size = 14)
  ) +
  geom_boxplot(outlier.shape = NA, alpha = 0.7) +
  geom_point(
    position = position_jitterdodge(
      jitter.height = 0,
      jitter.width = 0.5,
      dodge.width = .1
    ),
    shape = 21,
    alpha = 0.4
  )#+
```

```
# coord_cartesian(ylim = c(0, 8))
print(q)
dev.copy(
  svg,
  file = paste0(filename, "_totalcellheight.svg"),
  width = 4,
  height = 4
)
dev.off ()

#to plot boxplots for Height of cell vs locfit data------------------
r <-
  ggplot(data = locfit.model,
         aes(
           x = factor(Cell_height),
           y = fitvalues,
           fill = Genotype
         )) +
  theme(plot.title = element_text(hjust = 0.5), text = element_text(size =
                                                                      14)) +
  labs(title = "E-cadherin levels at various Cell Heights", x = "Normalized Height", y = "E-cadherin
Levels(in AU)") +
  geom_boxplot(position = position_dodge(width = 0.8), outlier.shape = NA) +
  scale_y_continuous(labels = comma) +
  geom_point(
    alpha = 0.4,
    position = position_jitterdodge(jitter.width = .2, dodge.width = 0.8),
    shape = 21
  )#+
# coord_cartesian(ylim = c(0, 1750))
print(r)
dev.copy(
  svg,
  file = paste0(filename, "_locfit_boxplot.svg"),
  width = 4,
  height = 4
)
dev.off ()

#to plot lineplots for cell height  vs Ecad levels------------------
s <-
  ggplot(
    data = graph.lines,
    aes(
      x = Stackheight,
      y = Mean,
      group = Uniquecellname,
      fill = Genotype,
```

```
        color = Genotype
      )
    ) +
    theme(plot.title = element_text(hjust = 0.5), text = element_text(size =
                                                                        14)) +
    labs(title = "Height vs Ecad Levels",
         x = "Height(in " ~ (mu * m),
         y = "E-cadherin Levels(in AU)") +
    scale_y_continuous(labels = comma) +
    geom_point(position = position_jitter(width = .1),
               shape = 21,
               alpha = 0.4) +
    geom_line(alpha = 0.4)#+
  # coord_cartesian(ylim = c(0, 1750))
  print(s)
  dev.copy(
    svg,
    file = paste0(filename, "_Rawheight.svg"),
    width = 4,
    height = 4
  )
  dev.off ()

  #to plot lineplots for Normalized cell height  vs Ecad levels----------------
  u <-
    ggplot(
      data = graph.lines,
      aes(
        x = Normalizedheight,
        y = Mean,
        group = Uniquecellname,
        fill = Genotype,
        color = Genotype
      )
    ) +
    theme(plot.title = element_text(hjust = 0.5), text = element_text(size =
                                                                        14)) +
    labs(title = "Normalized Height vs Ecad Levels", x = "Normalized Height", y = "E-cadherin Levels(in
  AU)") +
    geom_point(shape = 21, alpha = 0.4) +
    scale_y_continuous(labels = comma) +
    geom_line(alpha = 0.4) +
    #coord_cartesian(ylim = c(0, 1750))#+
    facet_grid(facets = . ~ Boundary)
  print(u)

  dev.copy(
    svg,
```

```
  file = paste0(filename, "_Normalized_height.svg"),
  width = 4,
  height = 4
)
dev.off ()


#to plot lineplots for Normalized cell height  vs Normalized Ecad levels------------
t <-
  ggplot(
    data = graph.lines,
    aes(
      x = Normalizedheight,
      y = NormalizedEcad,
      group = Uniquecellname,
      fill = Genotype
      # color = Genotype
    )
  ) +
  theme(
    plot.title = element_text(hjust = 0.5),
    text = element_text(size = 14),
    legend.position = "none",
    axis.text.x = element_text(angle = 45, hjust = 1)
  ) +
  scale_y_continuous(labels = comma) +
  labs(title = "E-Cadherin Distribution", x = "Normalized Height", y = "Normalized E-cadherin Levels")
+
  # stat_smooth(aes(
  #   x = Normalizedheight,
  #   y = NormalizedEcad,
  #   group = Genotype,
  #   fill = Genotype,
  #   color = Genotype
  # ),method='lm')+
  geom_bin2d(bins = 15, alpha = 0.3) +
  geom_point(shape = 21, alpha = 0.15) + facet_grid(. ~ Boundary) #+

# stat_density_2d(aes(colour = Genotype), geom="density_2d", alpha= 0.2)
print(t)

dev.copy(
  svg,
  file = paste0(filename, "_Normalized height Normalized Ecad.svg"),
  width = 4,
  height = 4
)
dev.off ()
```

```
#to plot Percent Abnormal Distribution------------
z <-
  ggplot(linear.reg.noise,
         aes(x = Genotype, y = Percentage, fill = Distribution)) +
  geom_bar(stat = "identity", position = "stack") + scale_fill_brewer(palette = "Dark2") +
  scale_y_continuous(labels = comma) +
  theme(
    plot.title = element_text(hjust = 0.5),
    text = element_text(size = 14),
    axis.text.x = element_text(angle = 45, hjust = 1)
  ) +
  labs(title = "Percent Boundaries showing \nAbnormal Distribution", x = "Genotype", y =
"Percentage")


print(z)
dev.copy(
  svg,
  file = paste0(filename, "_abnormal distribution.svg"),
  width = 4,
  height = 4
)
dev.off ()

#to plot apical perimeter vs genotype------------
b <-
  ggplot(data = apical.perimeter, aes(x = Genotype, y = Length, fill = Genotype)) +
  theme(
    plot.title = element_text(hjust = 0.5),
    text = element_text(size = 14),
    legend.position = "none",
    axis.text.x = element_text(angle = 45, hjust = 1)
  ) +
  labs(title = "Apical Perimeter of Cells",
       x = "Genotype",
       y = "Length(in" ~ (mu * m)) +
  geom_boxplot(outlier.shape = NA, alpha = 0.7) +
  scale_y_continuous(labels = comma) +
  theme(
    plot.title = element_text(hjust = 0.5),
    legend.position = "none",
    text = element_text(size = 14)
  ) +
  geom_point(position = position_jitter(width = .1),
             shape = 21,
             alpha = 0.4)#+
```

```
# coord_cartesian(ylim = c(40, 120))
print(b)
dev.copy(
  svg,
  file = paste0(filename, "_apical perimeter.svg"),
  width = 4,
  height = 4
)
dev.off ()



# multiplot graphs-------------------------------------
# To plot multiple graphs in one page

grid.newpage()
pushViewport(viewport(layout = grid.layout(17, 6)))
vplayout <- function(x, y)
  viewport(layout.pos.row = x, layout.pos.col = y)
print(r, vp = vplayout(1:6, 2:6))
print(p, vp = vplayout(7:11, 1:2))
print(q, vp = vplayout(7:11, 3:4))
print(b, vp = vplayout(7:11, 5:6))
print(t, vp = vplayout(12:17, 1:4))
print(z, vp = vplayout(12:17, 5:6))
dev.copy(
  svg,
  file = paste0(filename, "multiplot_all.svg"),
  width = 10,
  height = 10
)
dev.off()
```

```
# Supplementary File 2- R Script for Figure 4, 5, Figure 4-figure supplement 2
#
#
# This is a program to calculate various parameters for E-cadherin quantifications-----
#
#
#
# "rawdata" contains data read from the csv file(provided by user). It should contain the following columns:
#   1) Genotype
#   2) Cell name
#   3) Mean
#   4) Length
#   5) Boundary -- If you are doing cell wise analysis(put same letter in the whole column in case analysis is cell wise and by boundaries)
#
#
# "stack.all" contains stack wise calculated data which includes:
#   1) Height at a particular stack
#   2) Normalized Height
#   3) Normalized Ecad
#   4) Absolute Ecad at each stack

#clears memory--------
rm(list = ls())

#sets a working directory-----------
#This is where all your graphs will be saved

setwd(
  "D:/Prateek/TIFR/MS Lab/Ecad Gradient/locfit analysis/R files version control/Ecad_project/"
)

#libraries required for the program to work.-----------------
# List of libraries required for the program to run
pkg <- c("scales", "locfit","tidyr","purrr","dplyr","ggplot2","broom",
         "data.table", "RCurl","stringr", "grid", "gridExtra", "RColorBrewer")

# Check if the libraries are installed and install libraries not present
new.pkg <- pkg[!(pkg %in% installed.packages())]
if (length(new.pkg)) {
  install.packages(new.pkg)
}
rm("pkg","new.pkg")

#load libraries in the memory
library(scales)
library(locfit)
```

```
library(tidyr)
library(purrr)
library(dplyr)
library(ggplot2)
library(data.table)
library(RCurl)
library(stringr)
library(grid)
library(gridExtra)
library(RColorBrewer)

#stops warnings from being displayed
options(warn = -1)



# read raw data------------------
# the file should should have the following components:
# Genotype        Cell name          Area      Mean    Boundary

#Folder and file names where your input file is saved

Folderadd <-
  "D:/Prateek/TIFR/MS Lab/Ecad Gradient/locfit analysis/R files version control/Ecad_project/"
filename <-
  "Autonomous peri Lgl 05052019 14122018_outliers removed"
format <- ".csv"
fulladdress <- paste(Folderadd, filename, format, sep = "")

rawdata <-
  read.csv(fulladdress,
           header = TRUE,
           sep = ",") %>% na.omit()

# data arranging--------------------

#To have Genotype and Cell name, Boundary order as in the file (Otherwise it comes alphabetically
which may not be desirable)
rawdata$Genotype <-
  factor(rawdata$Genotype, levels = unique(rawdata$Genotype))
rawdata$Cell.name <-
  factor(rawdata$Cell.name, levels = unique(rawdata$Cell.name))
rawdata$Boundary <-
  factor(rawdata$Boundary, levels = unique(rawdata$Boundary))


#group by genotype and cell name calculate absolute ecad for each stack, and nest the data to to
help calculations on groups
```

```
by_cellname <- rawdata %>%
  group_by(Genotype, Cell.name) %>%
  mutate(AbsoluteEcad = Mean * Area) %>%
  nest()


#function to find height
rawheight <- function(df) {
  height <- vector("double", nrow(df))
  for (i in seq_along(df$Mean)) {
    height[i] <- (i - 1) * 0.28 #the height of the z slice
  }
  height
}


#function to normalize height
normheightfun <- function(df) {
  nheight <- vector("double", nrow(df))
  maxh <- nrow(df)
  minh <- 1
  for (i in seq_along(df$Mean)) {
    nheight[i] <- ((i) - minh) / (maxh - minh)
  }
  nheight
}

#function to normalize ecad levels
normecadfun <- function(df) {
  necad <- vector("double", nrow(df))
  maxe <- max(df$Mean)
  mine <- min(df$Mean)
  for (i in seq_along(df$Mean)) {
    necad[i] <- (df$Mean[i] - mine) * 100 / (maxe - mine)
  }
  necad
}

#dataframe which now has height per stack, normalized height, normalized ecad per genotype-cell
name
stack.all <- by_cellname %>%
  mutate(
    Stackheight = data %>% map(rawheight),
    Normalizedheight = data %>% map(normheightfun),
    NormalizedEcad = data %>% map(normecadfun),
    Uniquecellname = paste(Genotype, Cell.name)
  ) %>% unnest()
```

```
#locfit ---------------------------------------------
# locfit will fit the data cell wise and predict values at given cell heights

#nest the data in stack.all with normalized height to use in locfit for prediction
locfit.input <- stack.all %>%
  group_by(Genotype, Cell.name) %>%
  nest()

# locfit.input$data[[1]]


#cell heights chosen to interpolate ecad levels
predictvalues <- c(0.1, 0.3, 0.5, 0.7, 0.9)

#To calculate the y-axis limits in locfit graphs
ylimmax <- max(rawdata$Mean) + 250
ylimmin <- min(rawdata$Mean) - 100


#function to fit locfit
locfit_model <- function(df) {
  fit <- locfit(Mean ~ Normalizedheight, data = df)
  plot(
    fit,
    get.data = TRUE,
    ylim = c(ylimmin, ylimmax),
    xlim = c(0, 1),
    xlab = "Normalized Cell Height",
    ylab = "Ecad Level(in AU)"
  )
  par(new = TRUE)   #To superimpose graphs
  predictions <-  predict(fit, predictvalues)

}

#to plot the locfit graphs
#to start a new graph page and close all old ones,
# so that new graphs don't get superimposed on the old ones
plot.new()
dev.off()
plot.new()




#add locfit data to grouped cells and add cell height(on which prediction is made) to each cell
locfit.model <- locfit.input %>%
```

```
  mutate(fitvalues = data %>% map(locfit_model)) %>%
  unnest(fitvalues) %>%
  group_by(Genotype, Cell.name) %>%
  mutate(Cell_height = predictvalues)
locfit.model <- locfit.model %>%  group_by(Genotype, Cell.name)

#to save graph for locfit data
dev.copy(
  svg,
  file = paste0(filename, "_locfitgraphs.svg"),
  width = 4,
  height = 4
)

dev.off ()


#for creating groups for each cell for ggplot graph of lines -------------------------------------------------

graph.lines <- stack.all %>% mutate (Uniquecellname = paste(Genotype, Cell.name))




#writing csv--------------------------------------------------------


#write csv with height, normalized height,absolute ecad in each stack
write.csv(
  stack.all,
  file = paste0(filename, "_stackwise_calculations.csv"),
  row.names = FALSE
)


#write csv with locfit fitdata
write.csv(locfit.model,
          file = paste0(filename, "_locfitdata.csv"),
          row.names = FALSE)

#graphs-----------------------------------------------------------

#to plot boxplots for Height of cell vs locfit data------------------
r <-
  ggplot(data = locfit.model,
         aes(
           x = factor(Cell_height),
           y = fitvalues,
```

```
            fill = Genotype
          )) +
  theme(plot.title = element_text(hjust = 0.5), text = element_text(size =
                                                                      14)) +
  labs(title = "E-cadherin levels at various Cell Heights", x = "Normalized Height", y = "E-cadherin
Levels(in AU)") +
  geom_boxplot(position = position_dodge(width = 0.8), outlier.shape = NA) +
  scale_y_continuous(labels = comma) +
  geom_point(
    alpha = 0.4,
    position = position_jitterdodge(jitter.width = .2, dodge.width = 0.8),
    shape = 21
  )#+
# coord_cartesian(ylim = c(0, 1750))
print(r)
dev.copy(
  svg,
  file = paste0(filename, "_locfit_boxplot.svg"),
  width = 4,
  height = 4
)
dev.off ()

#to plot lineplots for cell height  vs Ecad levels------------------
s <-
  ggplot(
    data = graph.lines,
    aes(
      x = Stackheight,
      y = Mean,
      group = Uniquecellname,
      fill = Genotype,
      color = Genotype
    )
  ) +
  theme(plot.title = element_text(hjust = 0.5), text = element_text(size =
                                                                      14)) +
  labs(title = "Height vs Ecad Levels",
       x = "Height(in " ~ (mu * m),
       y = "E-cadherin Levels(in AU)") +
  scale_y_continuous(labels = comma) +
  geom_point(position = position_jitter(width = .1),
             shape = 21,
             alpha = 0.4) +
  geom_line(alpha = 0.4)#+
# coord_cartesian(ylim = c(0, 1750))
print(s)
dev.copy(
```

```
  svg,
  file = paste0(filename, "_Rawheight.svg"),
  width = 4,
  height = 4
)
dev.off ()

#to plot lineplots for Normalized cell height  vs Ecad levels----------------
u <-
  ggplot(
    data = graph.lines,
    aes(
      x = Normalizedheight,
      y = Mean,
      group = Uniquecellname,
      fill = Genotype,
      color = Genotype
    )
  ) +
  theme(plot.title = element_text(hjust = 0.5), text = element_text(size =
                                                                      14)) +
  labs(title = "Normalized Height vs Ecad Levels", x = "Normalized Height", y = "E-cadherin Levels(in
AU)") +
  geom_point(shape = 21, alpha = 0.4) +
  scale_y_continuous(labels = comma) +
  geom_line(alpha = 0.4) +
  #coord_cartesian(ylim = c(0, 1750))#+
  facet_grid(facets = . ~ Boundary)
print(u)

dev.copy(
  svg,
  file = paste0(filename, "_Normalized_height.svg"),
  width = 4,
  height = 4
)
dev.off ()


# multiplot graphs-------------------------------------
# To plot multiple graphs in one page

grid.newpage()
pushViewport(viewport(layout = grid.layout(17, 6)))
vplayout <- function(x, y)
  viewport(layout.pos.row = x, layout.pos.col = y)
print(r, vp = vplayout(1:6, 2:6))
# print(p, vp = vplayout(7:11, 1:2))
```

```
# print(q, vp = vplayout(7:11, 3:4))
# print(b, vp = vplayout(7:11, 5:6))
# print(t, vp = vplayout(12:17, 1:4))
# print(z, vp = vplayout(12:17, 5:6))
dev.copy(
  svg,
  file = paste0(filename, "multiplot_all.svg"),
  width = 10,
  height = 10
)
dev.off()
```
